# Pesticide exposure and multidomain symptom burden in Parkinson’s disease: a longitudinal triangulation study

**DOI:** 10.1038/s41531-026-01510-9

**Published:** 2026-08-05

**Authors:** Zhengjie Yang, Shijv Shen, Saskia Preissner, Robert Preissner

**Affiliations:** 1 Institute of Physiology, Charité–Universitätsmedizin Berlin, Philippstr. 12, 10115 Berlin, Germany; 2 Department of Oral and Maxillofacial Surgery, Charité–Universitätsmedizin Berlin, Augustenburger Platz 1, 13353 Berlin, Germany; 3 Department of Congenital Heart Disease and Pediatric Cardiology, University Hospital of Schleswig-Holstein, Campus Kiel, Kiel, Germany

**Keywords:** Diseases, Medical research, Neurology, Neuroscience, Risk factors

## Abstract

Although pesticide exposure has been linked to worse motor symptoms in Parkinson’s disease (PD), its associations with non-motor burden and with post-diagnostic progression rate remain unclear. We analyzed two large longitudinal cohorts, PPMI Online (*n* = 7190) and Fox Insight (*n* = 2298), using linear mixed-effects models and Cox regression, with supportive triangulation in an EHR cohort. In PPMI Online, pesticide exposure was associated with higher MDS-UPDRS Part II scores (β = 1.97, 95% CI 1.34–2.60), lower PDAQ-15 scores (β = −2.40, 95% CI −3.23 to −1.57), and greater anxiety, daytime sleepiness, and sleep disturbance. Motor and cognition-related estimates were directionally similar in Fox Insight. Time interaction estimates showed no consistent exposure-related differences. Primary Cox models indicated earlier motor milestones in both cohorts and earlier sleepiness and anxiety milestones in the PPMI Online cohort. Co-exposure to more pesticide classes was associated with worse motor function in both cohorts. TriNetX also showed elevated risks across five ICD-coded symptom domains (RRs 1.44–1.87), although these findings should be interpreted cautiously because the outcomes were defined differently. Further longitudinal studies using harmonised exposure assessments and repeated multidomain measures are needed to clarify the relationship between pesticide exposure and symptom trajectories after PD diagnosis.

## Introduction

Parkinson’s disease (PD) is one of the fastest-growing neurodegenerative disorders worldwide in terms of disease burden^1^. In 2021, an estimated 11.8 million people were living with PD globally, accounting for ~7.47 million disability-adjusted life years (DALYs). Compared with 1990, these figures represent increases of about 274 and 162%, respectively^2^. Notably, even after adjustment for population age structure, the age-standardised prevalence and DALY rates of PD continued to increase^1,2^. This pattern suggests that the rising burden of PD cannot be fully explained by population aging alone and that environmental exposures and lifestyle factors may also contribute substantially^1,3,4^.

Although the diagnosis of PD is primarily based on core motor features, such as bradykinesia, rigidity, resting tremor, and postural instability, non-motor symptoms also substantially affect the quality of life of both patients and caregivers, contributing greatly to the overall disease burden^5,6^. These symptoms include cognitive impairment, sleep disturbances, mood changes, autonomic dysfunction, and pain. Some non-motor symptoms may appear more than a decade before the onset of motor symptoms and continue to worsen over the course of the disease, yet effective treatments remain limited^5^. A systematic review of 70 studies found that, among the five core symptom domains affecting health-related quality of life (HRQOL), three were non-motor domains, and mood disorders were the most important determinant of HRQOL in the early stage of PD^7^. Therefore, identifying modifiable factors that influence the development and progression of non-motor symptoms is important for a more complete understanding of the long-term burden of PD.

The development of PD is influenced by both genetic and non-genetic factors^8^. Known monogenic forms account for only a subset of cases, and in populations without strong genetic determinants, the heritability of PD is estimated to be only 20–30%^9–11^. This pattern suggests that non-genetic factors, including environmental exposures, play an important role. Among the many candidate environmental factors, pesticide exposure is the most consistently supported environmental risk factor for PD^12–16^. Both residential and occupational pesticide exposure have been associated with an increased risk of PD, and the excess risk often exceeds 40%^17^. Evidence also supports a dose–response relationship^18^. In addition, more frequent contact, closer proximity to exposure, and longer exposure duration may be associated with an earlier age at onset, whereas the use of protective equipment may partly reduce this risk^19–21^.

Beyond factors related to disease onset, increasing attention has been directed toward determinants of PD progression, because patients often live with the disease for many years or even decades after diagnosis, and progression rates vary substantially across individuals^22,23^. In sporadic PD, environmental factors may explain the development of certain severe subtypes better than genetic factors. In the Oxford Discovery and Tracking Parkinson’s cohorts, the subtype characterised by more severe motor symptoms and poorer psychological well-being paradoxically showed a lower common PD genetic risk score and a more pronounced pro-inflammatory profile early in the disease course. This finding suggests that the development of this phenotype may be more strongly driven by exogenous factors^24^. In addition, factors that influence disease onset do not necessarily affect disease progression in the same direction. Although smoking has been relatively consistently associated with a lower risk of developing PD, prospective cohort studies have shown that it is associated with faster motor and cognitive decline^25,26^.

As another environmental factor supported by substantial evidence, pesticide exposure has also attracted increasing attention for its potential influence on symptom progression after PD onset^27–30^. Existing longitudinal studies suggest that pesticide exposure may be associated not only with more severe motor symptoms in patients with idiopathic PD, but also with an earlier onset of severe motor impairment, cognitive decline, and depressive symptoms^28^. However, the available evidence is still largely limited to motor symptoms and a small number of non-motor outcomes, and studies examining the progression of a broader range of non-motor symptoms, such as sleep disturbances and anxiety, remain scarce. Meanwhile, the roles of specific exposure characteristics, including combined exposure to multiple pesticides, exposure duration, and the use of personal protective measures, have not been systematically evaluated in relation to disease progression.

Fox Insight and the Parkinson’s Progression Markers Initiative Online Study (PPMI Online) provide an important foundation for addressing these questions. Both are longitudinal cohorts of patients with PD that repeatedly assess motor and non-motor symptoms during follow-up and collect information on occupational pesticide exposure and related exposure characteristics through validated questionnaires^31,32^. At the same time, both cohorts rely on self-reported exposure and recruit participants through online platforms. To examine whether the overall pattern of findings was also seen in a different type of data source, we further conducted a triangulation analysis in the TriNetX Global Collaborative Network, a large federated electronic healthcare record (EHR) database covering 171 healthcare organizations.

**Fig. 1:**
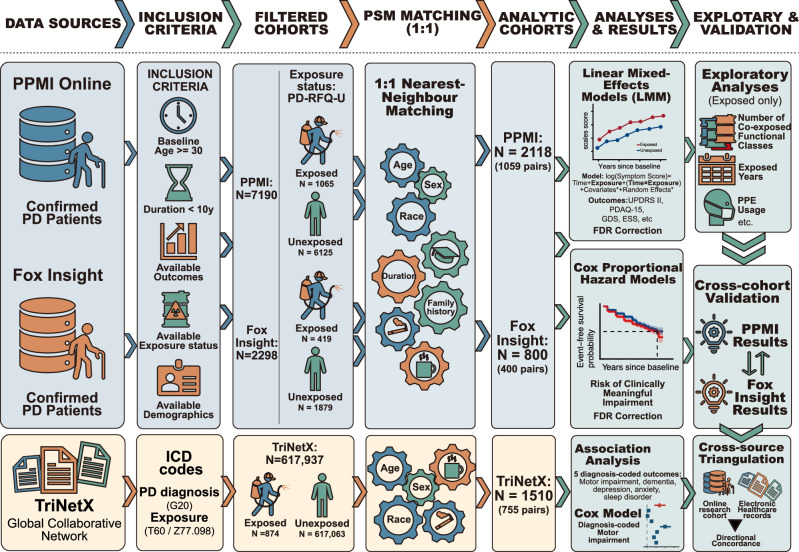
Study design and analytical workflow. The study was conducted on three data sources: two online longitudinal PD cohorts (PPMI Online and Fox Insight) and one electronic health record database (TriNetX Global Collaborative Network). Upper panel: PD patients from PPMI Online and Fox Insight were included if they met all of the inclusion criteria. After screening, 7190 (PPMI Online) and 2298 (Fox Insight) patients were retained. Pesticide exposure status was determined using the information from the PD risk factor questionnaire–updated (PD-RFQ-U). Propensity score matching (PSM) was performed using 1:1 nearest-neighbour matching, with covariates including age, sex, race, education, disease duration, family history of PD, smoking history, and coffee consumption history, yielding matched analytic cohorts of 2118 (PPMI Online) and 800 (Fox Insight) patients. Primary analyses comprised linear mixed-effects models (LMM) assessing symptom severity and rate of progression across six outcome scales (MDS-UPDRS Part II, PDAQ-15, GDS, ESS, PDSS-2, PAS), and Cox models assessing the risk of reaching clinically meaningful impairment thresholds. Exploratory analyses were conducted within the exposed group to examine the effects of exposure details. Lower panel: For directional triangulation, PD patients (ICD-10: G20) were identified in the TriNetX Global Collaborative Network. Pesticide exposure was defined by diagnosis codes. After 1:1 PSM on age, sex, race, nicotine dependence and alcohol dependence, 755 matched pairs were retained. Association analyses compared risks of five diagnosis-coded outcomes, and a Cox-model assessed the motor function milestone. Results from the two online cohorts and the EHR database were then assessed for directional concordance across data sources. * Covariates in LMM models included baseline age, sex, and disease duration at baseline. Patient ID was included as a random effect. Both random intercepts and random slopes were fitted. PD Parkinson’s disease, MDS-UPDRS Part II MDS-Unified Parkinson’s Disease Rating Scale Part II, PDAQ-15 Penn Parkinson’s Daily Activities Questionnaire-15, GDS geriatric depression scale, ESS Epworth sleepiness scale, PAS Parkinson anxiety scale; PDSS-2 Parkinson’s disease sleep scale-2.

Using this multi-source design, this study had four aims: (1) apply linear mixed-effects models to evaluate the associations between occupational pesticide exposure and the longitudinal burden and progression of motor symptoms and multiple non-motor symptom domains, including cognition, depression, anxiety, daytime sleepiness, and sleep disturbance; (2) apply Cox proportional hazards models to examine the associations between pesticide exposure and the risk of key milestone events; (3) to explore the modifying roles of exposure characteristics, including the number of pesticide classes involved in combined exposure, cumulative exposure duration, specific pesticide categories, and the use of personal protective equipment (PPE); and (4) to assess the directional concordance of the main findings in an independent EHR database with different exposure definitions and outcome measures. By integrating longitudinal symptom trajectories, clinical threshold events, and cross-source triangulation, this study sought to provide a more comprehensive understanding of the role of pesticide exposure in PD progression (Fig. 1).

## Results

### Propensity score matching

In the PPMI Online cohort, 7190 patients with PD met the inclusion criteria, including 1065 in the exposed group and 6125 in the unexposed group. After 1:1 nearest-neighbour matching, 1,059 matched pairs (*n* = 2118) were retained. All covariates had absolute standardised mean differences below 0.1, indicating good balance between the two groups with respect to baseline age, sex, disease duration, years of education, family history of PD, smoking history, coffee consumption history, race and ethnicity (Table 1).

**Table 1:** Baseline characteristics of PD patients with and without occupational pesticide exposure before and after propensity score matching in PPMI Online and Fox Insight cohorts

	Before matching	After matching						
**Characteristic**	PD-pesticide	PD+ pesticide	*p*	SMD	PD- pesticide	PD+ pesticide	*p*	SMD
**PPMI online**								
Sample size	6125	1065	-	-	1059	1059	-	-
Baseline age	67.53 (8.85)	67.23 (8.75)	0.306	0.034	67.21 (8.93)	67.23 (8.73)	0.956	0.002
Sex			<0.001	0.536			0.958	0.005
Male	3315 (54.1)	837 (78.6)			833 (78.7)	831 (78.5)		
Female	2810 (45.9)	228 (21.4)			226 (21.3)	228 (21.5)		
Disease duration	2.75 (2.44)	2.80 (2.43)	0.526	0.021	2.86 (2.46)	2.81 (2.43)	0.628	0.021
Education years	16.35 (3.37)	15.99 (3.12)	0.001	0.109	15.83 (3.15)	16.00 (3.13)	0.2	0.056
Family history			0.376	0.047			0.656	0.04
No	3619 (59.1)	650 (61.0)			647 (61.1)	645 (60.9)		
Yes	1629 (26.6)	262 (24.6)			248 (23.4)	262 (24.7)		
unknown	877 (14.3)	153 (14.4)			164 (15.5)	152 (14.4)		
Smoke			<0.001	0.181			0.527	0.049
No	3243 (52.9)	468 (43.9)			491 (46.4)	468 (44.2)		
Yes	1558 (25.4)	328 (30.8)			321 (30.3)	325 (30.7)		
unknown	1324 (21.6)	269 (25.3)			247 (23.3)	266 (25.1)		
Coffee			0.086	0.074			0.839	0.026
No	820 (13.4)	142 (13.3)			133 (12.6)	141 (13.3)		
Yes	3093 (50.5)	574 (53.9)			580 (54.8)	569 (53.7)		
unknown	2212 (36.1)	349 (32.8)			346 (32.7)	349 (33.0)		
Race			0.001	0.13			0.602	0.083
Asian	103 (1.7)	11 (1.0)			7 (0.7)	11 (1.0)		
Black	28 (0.5)	5 (0.5)			3 (0.3)	5 (0.5)		
White	5792 (94.6)	998 (93.7)			1004 (94.8)	998 (94.2)		
Multiracial	69 (1.1)	25 (2.3)			20 (1.9)	19 (1.8)		
Other	9 (0.1)	6 (0.6)			2 (0.2)	6 (0.6)		
unknown	124 (2.0)	20 (1.9)			23 (2.2)	20 (1.9)		
Ethnicity			<0.001	0.187			0.969	0.022
Ashkjew	560 (9.1)	52 (4.9)			55 (5.2)	52 (4.9)		
Hispanic	148 (2.4)	18 (1.7)			16 (1.5)	18 (1.7)		
Other	5241 (85.6)	951 (89.3)			946 (89.3)	945 (89.2)		
unknown	176 (2.9)	44 (4.1)			42 (4.0)	44 (4.2)		
**Fox Insight**								
Sample size	1879	419			400	400		
Baseline age	64.12 (8.99)	64.09 (9.57)	0.945	0.004	63.84 (9.65)	64.10 (9.62)	0.71	0.026
Sex			<0.001	0.411			0.762	0.027
Male	905 (48.2)	285 (68.0)			274 (68.5)	269 (67.2)		
Female	974 (51.8)	134 (32.0)			126 (31.5)	131 (32.8)		
Disease duration	3.17 (2.57)	3.33 (2.54)	0.228	0.065	3.19 (2.54)	3.28 (2.54)	0.62	0.035
Education level			0.353	0.096			1	0.005
Low	13 (0.7)	2 (0.5)			2 (0.5)	2 (0.5)		
Medium	593 (31.6)	149 (35.6)			143 (35.8)	142 (35.5)		
High	1268 (67.5)	266 (63.5)			254 (63.5)	255 (63.7)		
Unknown	5 (0.3)	2 (0.5)			1 (0.2)	1 (0.2)		
Family history			0.545	0.059			0.463	0.088
No	1140 (60.7)	242 (57.8)			238 (59.5)	232 (58.0)		
Yes	521 (27.7)	125 (29.8)			123 (30.8)	118 (29.5)		
unknown	218 (11.6)	52 (12.4)			39 (9.8)	50 (12.5)		
Smoke			<0.001	0.303			1	<0.001
No	1297 (69.0)	260 (62.1)			259 (64.8)	259 (64.8)		
Yes	570 (30.3)	135 (32.2)			135 (33.8)	135 (33.8)		
unknown	12 (0.6)	24 (5.7)			6 (1.5)	6 (1.5)		
Coffee			0.698	0.024			0.934	0.012
No	1410 (75.0)	310 (74.0)			305 (76.2)	307 (76.8)		
Yes	469 (25.0)	109 (26.0)			95 (23.8)	93 (23.2)		
Race			0.81	0.078			0.915	0.086
Asian	20 (1.1)	3 (0.7)			5 (1.2)	3 (0.8)		
Black	6 (0.3)	1 (0.2)			2 (0.5)	1 (0.2)		
White	1818 (96.8)	403 (96.2)			385 (96.2)	387 (96.8)		
Multiracial	16 (0.9)	6 (1.4)			4 (1.0)	5 (1.2)		
Other	6 (0.3)	2 (0.5)			1 (0.2)	2 (0.5)		
unknown	13 (0.7)	4 (1.0)			3 (0.8)	2 (0.5)		
Ethnicity			0.536	0.039			0.406	0.071
Hispanic	87 (4.6)	23 (5.5)			16 (4.0)	22 (5.5)		
other	1792 (95.4)	396 (94.5)			384 (96.0)	378 (94.5)		

Continuous variables are presented as mean (standard deviation); categorical variables are presented as *n* (%). Propensity scores were estimated using logistic regression with the following covariates identified via directed acyclic graph: baseline age, sex, race, ethnicity, education, disease duration at baseline, family history of PD, history of regular smoking, and history of regular coffee consumption. Matching was performed using 1:1 nearest-neighbour matching without replacement, with a caliper of 0.2 standard deviations of the logit of the propensity score. Balance was assessed using the standardised mean difference (SMD), with |SMD|<0.1 considered acceptable. *p* values for continuous variables were derived from two-sample *t*-tests; *p* values for categorical variables were derived from chi-squared tests.

*SMD* standardised mean difference, *PD−* pesticide, patients without occupational pesticide exposure, *PD+* pesticide, patients with occupational pesticide exposure.

In the Fox Insight cohort, 2298 patients with PD met the inclusion criteria, including 419 in the exposed group and 1879 in the unexposed group. After matching, 400 pairs (*n* = 800) were retained. The absolute standardised mean differences for all covariates were below 0.1, indicating good covariate balance (Table 1). For the analysis subset corresponding to each outcome scale, covariate balance was re-evaluated and confirmed before the longitudinal analyses were performed (Supplementary Table 1).

In the TriNetX database, 1:1 PSM on age, sex, race, ethnicity, smoking history, and history of alcohol dependence yielded 755 matched pairs, with all standardised mean differences below 0.1 (Supplementary Table 9).

### Longitudinal progression of motor and non-motor symptom severity

**Fig. 2:**
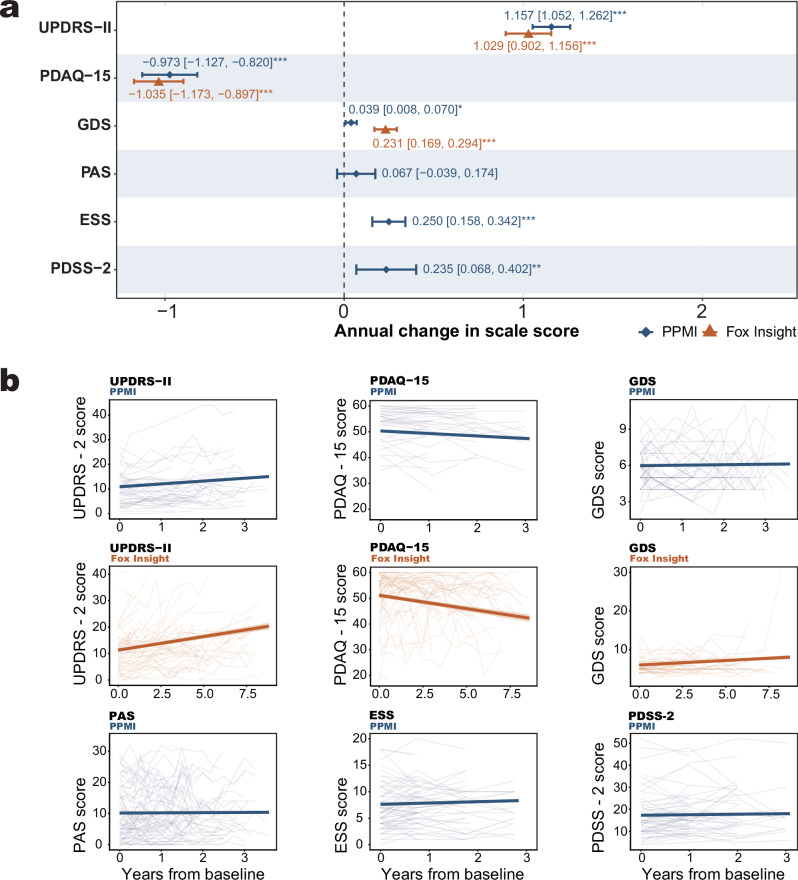
Longitudinal trajectories of motor and non-motor symptom progression in PD patients from the PPMI Online and Fox Insight cohorts. **a** Forest plot showing the annual rate of change (β coefficients with 95% confidence intervals) in motor and non-motor scale scores estimated from linear mixed-effects models, adjusted for baseline age, sex, and baseline disease duration. Blue diamonds represent estimates from the PPMI Online cohort, and orange triangles represent estimates from the Fox Insight cohort. Positive values indicate worsening over time for scales on which higher scores reflect greater impairment (MDS-UPDRS Part II, GDS, PAS, ESS, PDSS-2), whereas negative values indicate worsening for PDAQ-15, on which lower scores reflect greater impairment in cognitive-related daily function. MDS-UPDRS Part II and PDAQ-15 showed significant and consistent progression across both cohorts. GDS progression was significant in both cohorts, but with a markedly larger effect size in Fox Insight. PAS showed no significant change over time, while ESS and PDSS-2 (available only in PPMI Online) showed modest but significant worsening. Statistical significance: *p < 0.05; **p < 0.01; ***p < 0.001. **b** Spaghetti plots showing individual patient trajectories (thin lines) and group-level fitted trajectories (thick solid lines) for each scale over follow-up time. The top row shows MDS-UPDRS Part II, PDAQ-15, and GDS trajectories in PPMI Online (blue); the middle row shows the corresponding trajectories in Fox Insight (orange); the bottom row shows PAS, ESS, and PDSS-2 trajectories in PPMI Online (blue), which were not assessed in Fox Insight. The longer follow-up duration in Fox Insight (up to ~9 years) compared with PPMI Online (up to ~3.5 years) is reflected in the different x-axis ranges. Individual trajectories illustrate substantial between-patient heterogeneity around the group-level mean progression. MDS-UPDRS Part II movement disorder society–unified Parkinson’s disease rating scale part II, PDAQ-15 Penn Parkinson’s Daily Activities Questionnaire-15, GDS geriatric depression scale, PAS Parkinson anxiety scale, ESS Epworth sleepiness scale, PDSS-2 Parkinson’s disease sleep scale-2, CI confidence interval.

Figure 2 illustrates the progression of motor and non-motor symptoms among patients with PD in the two cohorts. After adjusting for baseline age, sex, and disease duration, both motor and non-motor symptoms progressed significantly over the follow-up period in both cohorts. For motor symptoms, the MDS-UPDRS Part II score increased by 1.16 points per year in the PPMI Online cohort (95% CI, 1.05–1.26; *p* < 0.001; Fig. 2) and by 1.03 points per year in the Fox Insight cohort (95% CI, 0.90–1.16; *p* < 0.001; Fig. 2).

For non-motor symptoms, cognition-related daily functioning also declined progressively over time in both cohorts. The PDAQ-15 score decreased by 0.97 points per year in the PPMI Online cohort (95% CI, −1.13 to −0.82; *p* < 0.001) and by 1.04 points per year in the Fox Insight cohort (95% CI, −1.17 to −0.90; *p* < 0.001). Depression scores, measured by the GDS, increased only slightly in the PPMI Online cohort (0.04 points per year, *p* = 0.014) but rose more rapidly in the Fox Insight cohort (0.23 points per year, *p* < 0.001). The three additional non-motor outcomes assessed only in the PPMI Online cohort also generally worsened over time. Daytime sleepiness, measured by the ESS, increased by 0.25 points per year (95% CI, 0.16 to 0.34; *p* < 0.001), and sleep disturbance, measured by the PDSS-2, increased by 0.24 points per year (95% CI, 0.07 to 0.40; *p* = 0.006). Anxiety, measured by the PAS, also showed a worsening trend over time, although the annual rate of change was not statistically significant (β = 0.07, *p* = 0.215). Full model estimates for overall longitudinal change across scales are provided in Supplementary Table 2.

### Association of pesticide exposure with symptom severity and progression rates

**Fig. 3:**
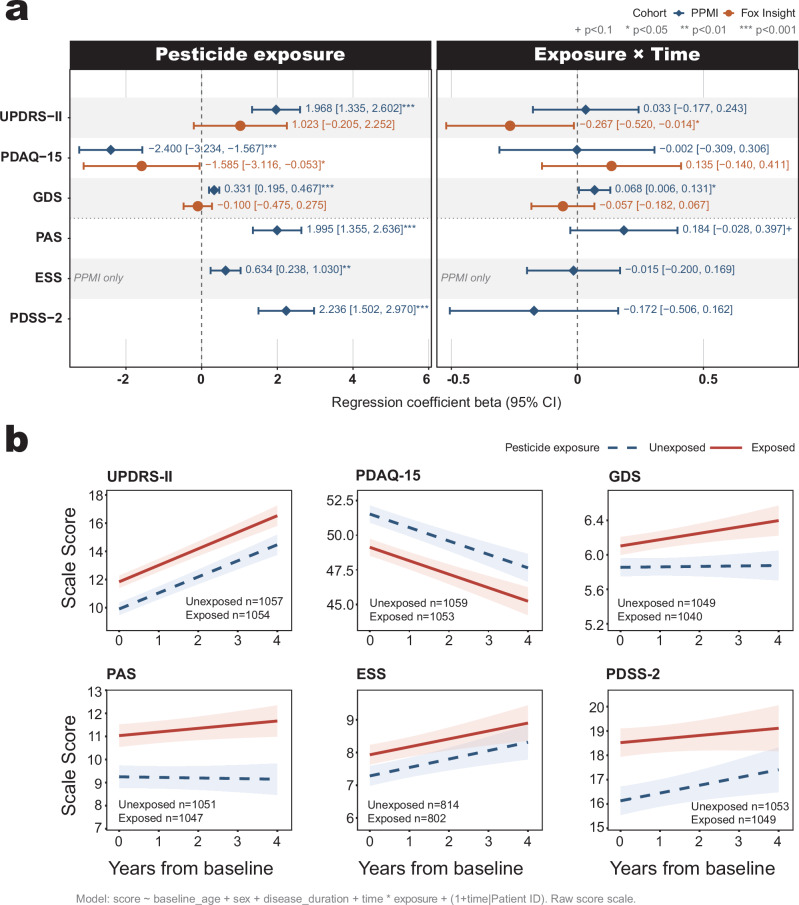
Associations between pesticide exposure and motor/non-motor symptom severity and progression in PD. **a** Forest plots showing regression coefficients (β with 95% CI) from linear mixed-effects models for the main effect of pesticide exposure (left panel) and the exposure × time interaction (right panel), across motor and non-motor scales in the PPMI Online (blue diamonds) and Fox Insight (orange circles) cohorts. Models were adjusted for baseline age, sex, and baseline disease duration, with random intercepts and slopes per patient (score ~ baseline_age + sex + disease_duration + time × exposure + (1 + time|Patient ID)). PAS, ESS, and PDSS-2 were assessed in PPMI Online only. In PPMI Online, pesticide-exposed patients showed significantly greater severity on MDS-UPDRS Part II (+ 1.97 points), PDAQ-15 (−2.40 points, with lower scores indicating worse function), GDS (+0.33), PAS (+2.00), ESS (+0.63), and PDSS-2 (+2.24). In Fox Insight, the effect directions for the shared outcomes were broadly similar for MDS-UPDRS Part II and PDAQ-15, but the associations were not consistently statistically robust after multiple-testing correction. In contrast, exposure × time interaction terms were largely non-significant across both cohorts and scales, indicating that pesticide exposure was predominantly associated with severity at the early stage rather than with accelerated progression. Significance levels: ⁺*p* < 0.1; *p < 0.05; **p < 0.01; ***p < 0.001. **b** Model-predicted mean trajectories (solid red lines for exposed, dashed blue lines for unexposed) with 95% confidence bands over four years of follow-up in the PPMI Online cohort, derived from the mixed-effects models in panel (**a**). Across all six scales, the exposed and unexposed groups exhibited parallel trajectories with a persistent vertical offset, visually confirming that pesticide exposure shifts the baseline severity upward (or downward for PDAQ-15) without substantially altering the slope of progression. Sample sizes for each scale are indicated within the panels.

**Fig. 4:**
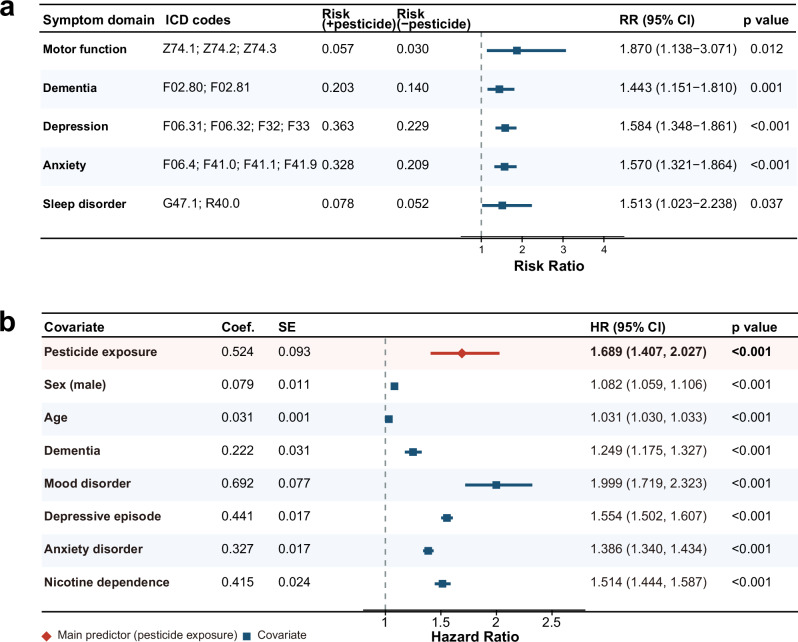
Cross-source triangulation of pesticide exposure and PD symptom burden in the TriNetX database. **a** Risk ratios (with 95% CI) from propensity score-matched analyses (*n* = 755 per group) comparing PD patients with ICD-10-coded pesticide exposure (T60 or Z77.098) to unexposed PD patients across five symptom domains. All five domains showed significantly elevated risk in the exposed group, directionally consistent with the primary findings from PPMI Online and Fox Insight. **b** Hazard ratios (with 95% CI) from a Cox proportional hazards model for the motor function milestone event (Z74.0-Z74.3). Pesticide exposure (red diamond) was the main predictor; covariates (blue squares) included sex, age at index, and five pre-diagnosis comorbidities. Pesticide exposure was independently associated with a 69% increased risk of reaching the motor function milestone (HR = 1.689, *p* < 0.001).

We further incorporated time, pesticide exposure, and their interaction term into the linear mixed-effects models to examine the associations of pesticide exposure with symptom levels at the earliest observed assessment and with the subsequent rate of change in patients with PD. The results are shown in Fig. 3. Full coefficient estimates for these models are provided in Supplementary Table 3. Pesticide exposure was associated with worse motor and non-motor symptom levels, whereas no consistent evidence was observed across the two cohorts that it affected the rate of symptom progression. To examine whether this overall pattern was also seen in a data source with different exposure definitions and outcome measures, we analyzed diagnosis-coded outcomes in the TriNetX EHR database. The TriNetX results showed directionally consistent associations between ICD-coded pesticide exposure and all five symptom domains (Fig. 4a).

In the PPMI Online cohort, the pesticide-exposed group had mean MDS-UPDRS Part II scores that were 1.97 points higher than those of the unexposed group (95% CI, 1.34–2.60; *p* < 0.001; *q* < 0.001), which was equivalent to nearly two years of natural progression in the unexposed group (annual progression rate, 1.14 points; Fig. 3a). A similar direction of association was observed in the Fox Insight cohort (β = 1.02, 95% CI, −0.21 to 2.25), although it did not reach statistical significance (*p* = 0.102; *q* = 0.154).

The exposed group also showed poorer cognition-related daily functioning in both cohorts. Compared with the unexposed group, the pesticide-exposed group had mean PDAQ-15 scores that were 2.40 points lower in the PPMI Online cohort (95% CI, −3.23 to −1.57; *p* < 0.001; *q* < 0.001) and 1.59 points lower in the Fox Insight cohort (95% CI, −3.12 to −0.05; *p* = 0.043; *q* = 0.128). For both scales, the exposure-by-time interaction term was not statistically significant and did not show a consistent direction across the two cohorts, suggesting that pesticide exposure was associated mainly with worse symptom levels at early observed assessment, rather than with a faster rate of progression in motor symptoms or cognition-related impairment.

The association between pesticide exposure and non-motor symptoms was particularly evident in the PPMI Online cohort. The exposed group had significantly higher mean scores than the unexposed group on the PAS, the PDSS-2, and the ESS. Specifically, PAS scores were 2.00 points higher (95% CI, 1.36-2.64; *p* < 0.001; *q* < 0.001), PDSS-2 scores were 2.24 points higher (95% CI, 1.50–2.97; *p* < 0.001; *q* < 0.001), and ESS scores were 0.63 points higher (95% CI, 0.24–1.03; *p* = 0.002; *q* = 0.002). The exposure-by-time interaction terms for all three scales were not statistically significant, indicating similar rates of progression between the exposed and unexposed groups for these symptoms (Fig. 3).

For depression, measured by the GDS, the two cohorts showed inconsistent results. In the PPMI Online cohort, the exposed group had a mean GDS score that was 0.33 points higher than that of the unexposed group (95% CI, 0.20-0.47; *p* < 0.001; *q* < 0.001), and the exposure-by-time interaction term was positive (β = 0.07, 95% CI, 0.01–0.13; *p* = 0.033), but this association was no longer statistically significant after correction for multiple testing (*q* = 0.191), and no consistent finding was observed in the Fox Insight cohort (Supplementary Fig. 2).

Evidence for an association between pesticide exposure and faster post-enrolment progression was limited and inconsistent. In the primary mixed-effects models, the 95% confidence intervals for seven of the nine exposure-by-time estimates included zero. The two exceptions occurred in different domains and pointed in opposite directions: PPMI Online showed a small positive GDS slope difference (0.07 points/year, 95% CI 0.01 to 0.13), while Fox Insight showed a negative MDS-UPDRS Part II slope difference (−0.27 points/year, 95% CI −0.52 to −0.01). Neither estimate was consistently reproduced across the sensitivity analyses. The full-cohort IPTW analysis retained all eligible participants and increased precision relative to the matched analysis; every exposure-by-time confidence interval included zero, including those for PPMI Online GDS (0.04 points/year, 95% CI −0.01 to 0.09) and Fox Insight MDS-UPDRS Part II (−0.10 points/year, 95% CI −0.26 to 0.06).

In the TriNetX triangulation analysis, PD patients with pesticide-related codes had higher risks of motor impairment (RR = 1.87, 95% CI 1.14–3.07), dementia (RR = 1.44, 95% CI 1.15–1.81), depression (RR = 1.58, 95% CI 1.35–1.86), anxiety (RR = 1.57, 95% CI 1.32–1.86), and sleep disorders (RR = 1.51, 95% CI 1.02–2.24) compared with those without such codes (all *p* < 0.05; Fig. 4a). Across all five symptom domains, the direction of associations was consistent with the pattern observed in the two online cohorts, in which pesticide exposure was associated with greater motor and non-motor symptom burden. Detailed association results for the five diagnosis-coded outcomes are provided in Supplementary Table 10.

### Cox proportional hazards regression

**Fig. 5:**
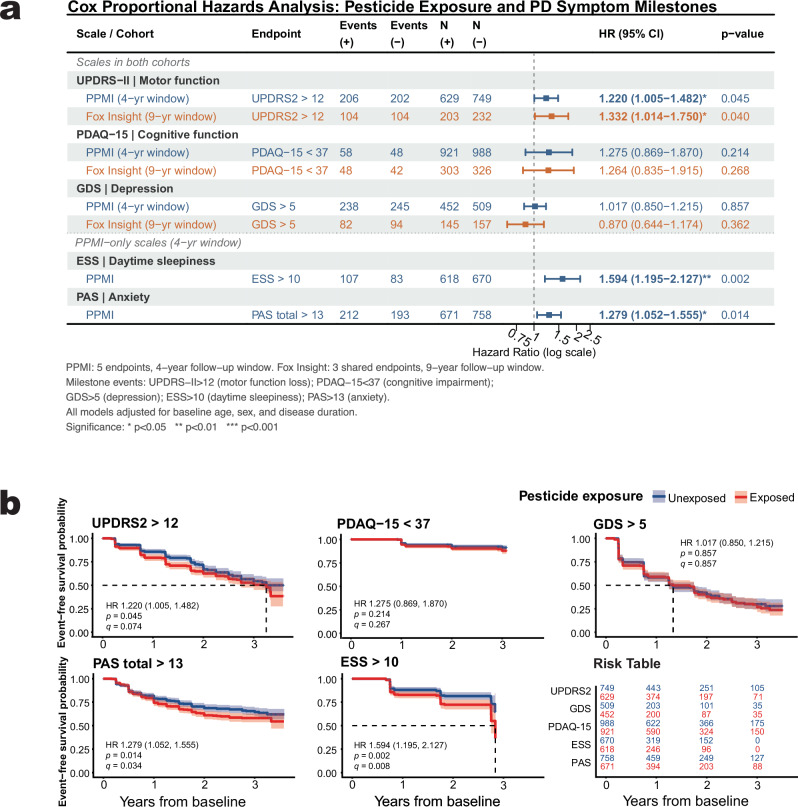
Cox proportional hazards analysis of pesticide exposure and risk of reaching PD symptom milestones. **a** Summary table and forest plot of hazard ratios (HRs) with 95% CI from Cox models evaluating the association between pesticide exposure and the risk of reaching clinically meaningful milestones across motor and non-motor domains. Models were adjusted for baseline age, sex, and baseline disease duration. The PPMI Online cohort was analyzed with a 4-year follow-up window for all five endpoints, whereas the Fox Insight cohort was analyzed with a 9-year follow-up window for the three shared endpoints (MDS-UPDRS Part II, PDAQ-15, and GDS); ESS and PAS were assessed in PPMI Online only. Pesticide exposure was associated with an increased risk of reaching the MDS-UPDRS Part II motor function milestone in both cohorts (PPMI Online: HR = 1.22, 95% CI 1.005–1.482, *p* = 0.045; Fox Insight: HR = 1.33, 95% CI 1.014–1.750, *p* = 0.040). Among non-motor endpoints, exposure was associated with significantly elevated risk of exceeding the ESS daytime sleepiness threshold (HR = 1.59, 95% CI 1.195–2.127, *p* = 0.002) and the PAS anxiety threshold (HR = 1.28, 95% CI 1.052–1.555, *p* = 0.014) in PPMI online. No significant associations were observed for PDAQ-15 or GDS milestones in either cohort. Significance levels: *p < 0.05; **p < 0.01; ***p < 0.001. **b** Kaplan–Meier event-free survival curves for the five milestone endpoints in the PPMI Online cohort, stratified by pesticide exposure status (blue = unexposed, red = exposed). Shaded bands represent 95% CI. HRs, unadjusted *p* values, and FDR-adjusted *q* values from the Cox models are annotated within each panel. The risk table (bottom right) shows the number of patients at risk at each time point for each scale, stratified by exposure status. Exposed patients consistently showed earlier and more frequent milestone events for MDS-UPDRS Part II, ESS, and PAS, while curves for PDAQ-15 and GDS largely overlapped between groups.

We further applied Cox regression models to examine the associations between pesticide exposure and the risk of reaching prespecified symptom milestone events during follow-up. The results are shown in Fig. 5 and Supplementary Table 4. After correction for multiple testing, patients in the pesticide-exposed group in the PPMI Online cohort had a 59% higher risk of developing clinically significant daytime sleepiness (ESS >10) than those in the unexposed group (HR = 1.59, 95% CI, 1.19–2.13; *q* = 0.008), as well as a 28% higher risk of developing clinically significant anxiety (PAS >13) (HR = 1.28, 95% CI, 1.05–1.55; *q* = 0.034).

For the motor milestone, moderate motor impairment (MDS-UPDRS Part II >12) showed a consistent trend toward increased risk in both cohorts. The hazard ratio was 1.22 in the PPMI Online cohort (95% CI, 1.00–1.48; *p* = 0.045) and 1.33 in the Fox Insight cohort (95% CI, 1.01–1.75; *p* = 0.040), although both associations remained borderline after multiple-testing correction (PPMI Online: *q* = 0.075; Fox Insight: *q* = 0.119). No significant associations were observed between pesticide exposure and the milestone events of decline in cognition-related daily functioning (PDAQ-15 <37) or clinically significant depression (GDS >5) in either cohort. Kaplan–Meier curves for the three shared Fox Insight endpoints are shown in Supplementary Fig. 3.

In the TriNetX triangulation analysis, pesticide exposure was also independently associated with the diagnosis-coded motor function milestone (HR = 1.69, 95% CI 1.41–2.03, *p* < 0.001) after adjustment for age, sex, and baseline comorbidities (Fig. 4b). This finding was directionally concordant with the trend observed in both online cohorts, where the motor milestone showed consistent but borderline-significant elevations in risk (Supplementary Table 11).

### Exploratory analysis of exposure characteristics

**Fig. 6:**
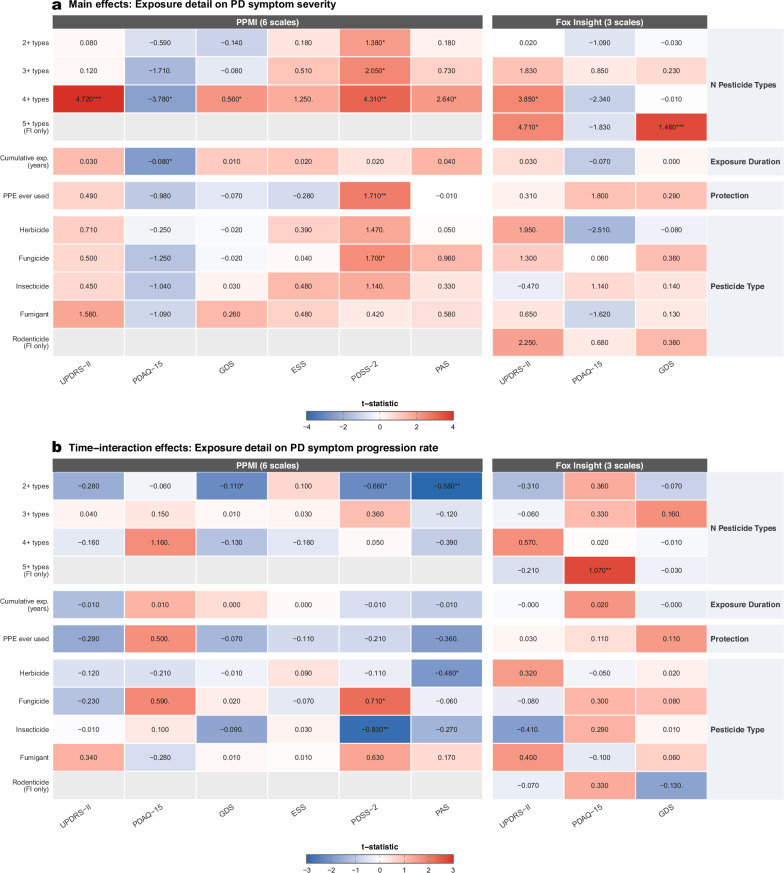
Exploratory analysis of pesticide exposure details on PD symptom burden and progression rate. **a** Main effects of exposure details on symptom severity. Heatmaps displaying regression coefficients (β) from linear mixed-effects models evaluating the associations between specific aspects of pesticide exposure (rows) and motor/non-motor scale scores (columns) in the PPMI Online cohort (left, six scales) and the Fox Insight cohort (right, three shared scales). Analyses were restricted to pesticide-exposed patients only, and examined five categories of exposure details: number of co-exposed pesticide types; cumulative exposure duration (in years); PPE usage; and specific pesticide functional classes. All models were adjusted for baseline age, sex, and baseline disease duration. A notable and cross-cohort consistent finding is the dose–response relationship between the number of co-exposed pesticide types and MDS-UPDRS Part II severity: compared with exposure to a single type, exposure to 4+ types was associated with a 4.72-point increase in PPMI Online (*p* < 0.001) and a 3.85-point increase in Fox Insight (*p* < 0.05), with a further increase to 4.71 points for 5+ types in Fox Insight. In contrast, cumulative exposure duration in years showed minimal and mostly non-significant associations. Associations with specific pesticide functional classes were generally weaker and less consistent across cohorts. **b** Time-interaction effects of exposure details on symptom progression rate. Heatmaps displaying the exposure-detail × time interaction coefficients from the same models. In contrast to the pronounced main effects observed in panel (**a**), the time-interaction terms were predominantly small and non-significant across both cohorts and all exposure categories. Scattered nominally significant interactions did not follow a consistent pattern across scales or cohorts and should be interpreted with caution in the context of multiple comparisons. Heatmap conventions: Cell colour represents the t-statistic of each coefficient (blue = protective or lower score; red = deleterious or higher score; colour scale capped at ±5 in panel (**a**) and ±3 in panel (**b**) for display purposes). Numerical values within cells show the raw regression coefficient (β). Grey cells indicate terms not available for the given cohort-scale combination. Significance levels: ⁺*p* < 0.1; *p < 0.05; **p < 0.01; ***p < 0.001.

Having established the overall association between pesticide exposure and symptom burden across the two online cohorts and the TriNetX database, we next examined whether specific exposure characteristics were associated with the magnitude of symptom burden. Using linear mixed-effects models within the exposed group of the two online cohorts, we evaluated the effects of the number of pesticide classes involved in combined exposure, cumulative exposure duration, specific pesticide types, and the use of PPE (Fig. 6).

The number of pesticide classes involved in combined exposure showed a consistent and significant dose–response relationship with motor impairment in both cohorts (Fig. 6a). Using exposure to one pesticide class as the reference, exposure to four pesticide classes was associated with a 4.72-point higher mean MDS-UPDRS Part II score in the PPMI Online cohort (95% CI, 2.40–7.03; *p* < 0.001) and a 3.85-point higher mean score in the Fox Insight cohort (95% CI, 0.77–6.93; *p* = 0.014). In the Fox Insight cohort, exposure to five pesticide classes was associated with an even larger increase of 4.71 points (95% CI, 0.95–8.47; *p* = 0.014). These findings indicate that combined exposure to multiple pesticide classes was associated with more severe motor impairment, and this pattern was replicated in two independent cohorts.

In the PPMI Online cohort, the number of pesticide classes involved in combined exposure also showed a similar increasing trend for PDSS-2 scores (two classes, +1.38, *p* = 0.043; three classes, +2.05, *p* = 0.014; four classes, +4.31, *p* = 0.002), although this scale was not available in Fox Insight and therefore could not be assessed for cross-cohort replication. In addition, exposure to a greater number of pesticide classes (four or five classes) was associated with poorer cognition-related daily functioning and more severe depressive symptoms in both cohorts, although some of these associations in the Fox Insight cohort were directionally consistent but did not reach statistical significance. Most interaction terms between the number of pesticide classes and time were not statistically significant (Fig. 6b), suggesting that combined exposure to multiple pesticide classes was primarily associated with average symptom severity rather than the rate of progression. Full model estimates for these dose–response analyses are provided in Supplementary Table 5.

In addition to the number of pesticide classes involved in combined exposure, we examined the relationships of cumulative exposure duration, PPE use, and specific pesticide types with symptom severity and progression rate within the exposed group. In these analyses, some individual exposure characteristics showed significant associations with specific scales in only one cohort. For example, cumulative exposure duration was negatively associated with the mean PDAQ-15 score in the PPMI Online cohort (β = −0.08 per year, *p* = 0.012), and fungicide exposure was positively associated with both the mean PDSS-2 score (β = 1.70, *p* = 0.020) and the rate of PDSS-2 progression (β = 0.71 per year, *p* = 0.026) in the same cohort. However, these associations were not consistently replicated in the other cohort, or the relevant scales were available only in one cohort. Therefore, these findings should be regarded as exploratory, and the full results are provided in the Supplementary Note 1 and Supplementary Tables 6 and 7.

### Sensitivity analyses

The associations with symptom level at the first observed assessment were broadly consistent across treatment, design, missing-data and prevalent-entry analyses (Supplementary Tables 12–16 and 20 and Supplementary Fig. 4). Adjustment for baseline or time-varying anti-parkinsonian medication, broad drug classes or DBS, and exclusion of participants with a DBS record produced little change in the estimates; for example, the PPMI Online MDS-UPDRS Part II coefficient was 1.97 (95% CI 1.34–2.60) in the primary model and 1.97 (1.34–2.61) after time-varying medication adjustment (Supplementary Table 12 panel A). Baseline treatment indicators were broadly similar between groups, although dopamine-agonist use showed modest residual imbalance in Fox Insight (SMD = 0.13, Supplementary Table 12 panel B). Matching retained 99.4% of exposed PPMI Online and 95.5% of exposed Fox Insight participants, and mainly reduced the surplus unexposed group (Supplementary Table 15). Full-cohort IPTW achieved measured covariate balance and yielded directionally concordant estimates, including MDS-UPDRS Part II coefficients of 2.41 (1.94–2.88) in PPMI Online and 1.55 (0.70–2.40) in Fox Insight (Supplementary Table 13). Complete-case, multiple-imputation, and less than 5-year disease-duration analyses preserved the principal PPMI Online severity pattern, with wider intervals in some reduced samples (Supplementary Tables 14 and 16). Across these specifications, exposure-by-time estimates remained small and inconsistent; the nominal positive PPMI Online GDS and negative Fox Insight MDS-UPDRS Part II interactions were not uniformly reproduced (Supplementary Tables 19 and 21).

The milestone estimates were also generally similar in direction across the parallel Cox sensitivity analyses, although they were not uniformly statistically significant in smaller or rematched analyses (Supplementary Table 22 and Supplementary Fig. 5). Adjustment for the corresponding baseline scale score attenuated the PPMI Online motor milestone from HR 1.22 (95% CI 1.00–1.48) to 1.09 (0.89–1.32), the anxiety milestone from 1.28 (1.05–1.55) to 1.17 (0.96–1.42), and the Fox Insight motor milestone from 1.33 (1.01–1.75) to 1.11 (0.84–1.47); the daytime-sleepiness milestone remained elevated at 1.40 (1.05–1.87) (Supplementary Table 17). Cox models using disease duration since diagnosis as the time scale yielded estimates similar to the primary models among enroled participants, but cannot address selection before cohort entry. Mean follow-up was similar by exposure status in both cohorts (Supplementary Table 18). Baseline-only participation did not differ materially in PPMI Online and was nominally more frequent among exposed Fox Insight participants (adjusted OR 1.70, 95% CI 1.00–2.88; *p* = 0.049), without a corresponding reduction in mean follow-up (Supplementary Table 18).

## Discussion

This study used two independent longitudinal PD cohorts, PPMI online and Fox Insight, together with a triangulation analysis in the TriNetX EHR database, to assess the associations of pesticide exposure with motor and non-motor symptom severity, rates of longitudinal progression, and the risk of milestone events. Several main findings emerged. First, in PPMI Online, occupational pesticide exposure was associated at the first observed assessment with worse motor function, poorer cognition-related daily functioning, and greater depressive symptoms, anxiety, daytime sleepiness, and nocturnal sleep disturbance. Fox Insight showed adverse-direction estimates for motor function and cognition-related daily functioning, while the depression association observed in PPMI Online was not reproduced; anxiety and sleep scales were unavailable in that cohort. The clearest cross-cohort concordance concerned motor function and cognition-related daily functioning. Second, exposure-by-time estimates showed no consistent pattern of faster post-baseline change across domains or cohorts. Third, the primary Cox models showed higher hazards of daytime-sleepiness and anxiety milestones in PPMI Online and nominally elevated motor milestone hazards in both cohorts. Fourth, TriNetX showed elevated risks across five diagnosis-coded domains among patients with ICD-coded pesticide exposure. These results provide broad domain-level triangulation. Differences in exposure ascertainment and diagnosis-coded outcome definitions preclude direct validation of the repeated symptom-scale findings. Fifth, exploratory analyses found worse motor function among participants reporting exposure to four or more pesticide classes in both cohorts. In contrast, the effects of other exposure characteristics, including cumulative exposure duration, early-life exposure, use of PPE, and specific pesticide types, were limited and were not consistently validated across cohorts. Taken together, the primary analyses suggested that pesticide exposure was more consistently associated with greater symptom burden than with a faster rate of subsequent progression. A broad set of sensitivity analyses generally supported this interpretation. Symptom-level associations were essentially unchanged after adjustment for available treatment indicators and remained directionally consistent under full-cohort weighting, alternative missing-data approaches, and shorter-disease-duration restriction. Exposure-by-time estimates remained generally close to the null, with isolated exceptions not consistently reproduced, while milestone estimates were directionally stable across alternative specifications but less precise in reduced samples and attenuated for motor and anxiety outcomes after baseline-severity adjustment.

A substantial body of literature has supported an association between pesticide exposure and the risk of developing PD, but longitudinal studies directly evaluating whether such exposure affects symptom progression after diagnosis remain limited. In the California PEG cohort, Li et al. used GIS-linked pesticide application records and identified ten specific pesticides associated with accelerated motor, cognitive, and depressive milestone events^28^. Using the same cohort, Paul et al. reported that the interaction between organophosphate exposure and the slow-metabolizer PON1 genotype was associated with faster progression across multiple symptom domains^33^. In the largest longitudinal analysis to date, Lüth et al. analyzed 5139 participants from PPMI Online and Fox Insight and found that pesticide exposure was significantly associated with more severe motor symptoms^29^. Santos-Lobato and Schuh further reported, in the PPMI cohort, that frequent household fungicide use was associated with an increased risk of accelerated cognitive impairment^30^.

The findings of the present study are broadly consistent with those of the studies discussed above, as all suggest that pesticide exposure is associated with worse symptom outcomes in PD. A key strength of our study is that, by including both the main effect of exposure and the exposure-by-time interaction in the linear mixed-effects models, we were able to distinguish differences in symptom levels from differences in progression rate. Our results suggest that pesticide exposure was associated mainly with worse symptom levels from the earliest observed assessments, rather than with faster longitudinal deterioration after that point. This finding does not conflict with the survival results reported in the PEG cohort, because earlier occurrence of milestone events in survival models may reflect worse baseline status rather than a steeper rate of decline^28^. In our study, the increased risks of ESS- and PAS-defined milestone events observed in the Cox models were consistent with the pattern seen in the linear mixed-effects models, in which the main effect of exposure was significant, whereas the interaction term was not. In other words, if the exposed group started at a worse symptom level but progressed at a similar rate, they would be expected to cross clinically relevant thresholds earlier, which is consistent with the Cox results.

In this study, symptom burden at cohort entry, post-enrolment change, and milestone timing describe different parts of the observed disease course. The exposure main effect estimates the adjusted difference in scale score at the first assessment. It represents the clinical state in which participants entered observation and may reflect symptoms accumulated before enrolment, treatment trajectories and therapeutic response, healthcare access and utilisation, disease subtype, functional reserve, comorbidities, reporting behaviour, and selection into an online cohort. The exposure-by-time interaction estimates the average difference in linear change during the observed follow-up period. It cannot reconstruct changes before enrolment or fully capture nonlinear and stage-dependent trajectories. Milestone timing depends on both the starting distance from a clinical threshold and subsequent change, so participants entering closer to a threshold may reach it earlier despite similar later slopes. Although we attempted to separate the effects of baseline severity and progression rate on milestone occurrence timing by adjusting for baseline scale scores, this approach could not disentangle causal cascade effects because baseline symptoms may already reflect the consequences of early exposure and disease evolution. The exposure main effects are thus best interpreted as differences in observed symptom burden at cohort entry. The available follow-up did not reveal a consistent difference in subsequent linear progression and cannot establish when the entry-level differences emerged. Studies recruiting participants close to diagnosis, with repeated treatment, healthcare-use, and multidomain assessments, are needed to trace the origin and evolution of these differences.

Concordance between PPMI Online and Fox Insight varied by outcome. Among the three scales assessed longitudinally in both cohorts, estimates for motor burden and cognition-related daily functioning were directionally concordant, indicating worse symptom burden among exposed participants in both cohorts. By contrast, the association with greater depressive symptoms observed in PPMI Online was not reproduced in Fox Insight. PPMI Online additionally assessed anxiety, daytime sleepiness, and nocturnal sleep disturbance; therefore, its larger number of detected associations partly reflects its coverage of six longitudinal scales, compared with three in Fox Insight. The cohorts also differed in sample size and follow-up structure. The matched analyses included 2,118 participants from PPMI Online and 800 from Fox Insight. In the matched MDS-UPDRS Part II samples, participants contributed, on average, 5.3 assessments over 1.7 years in PPMI Online and 7.6 assessments over 4.1 years in Fox Insight. Thus, PPMI Online provided denser sampling, whereas Fox Insight provided a longer observation window. In Fox Insight, full-cohort IPTW yielded directionally concordant and more precise estimates for motor function and cognition-related daily functioning, suggesting some loss of precision from 1:1 matching. Although binary exposure status was harmonised across cohorts, the underlying assessment differed: the PD-RFQ-U was administered up to twice and covered nine age periods in PPMI Online, compared with one administration and six age periods in Fox Insight. Participants in the eligible PPMI Online cohort were older at entry than those in Fox Insight (mean age, 67.5 versus 64.1 years) and had slightly shorter-disease duration (2.8 versus 3.2 years). Follow-up duration was similar across exposure status in both cohorts, although baseline-only participation was nominally more frequent among exposed Fox Insight participants. These differences in outcome coverage, sampling density, exposure assessment, and cohort composition support partial, outcome-specific cross-cohort replication, with the clearest concordance for motor burden and cognition-related daily functioning.

The PAS, ESS and PDSS-2 were available only in PPMI Online, so the anxiety, daytime-sleepiness and nocturnal sleep-disturbance findings lack replication in a second longitudinal cohort using the same instruments. Their stability across internal sensitivity analyses does not substitute for external replication. TriNetX provides only domain-level directional triangulation: its anxiety and somnolence outcomes were diagnosis-coded, it did not capture repeated scale trajectories or nocturnal sleep disturbance, and its exposure definition identified a small clinically recorded subgroup rather than the broader questionnaire-defined occupationally exposed population. The TriNetX estimates should therefore not be compared directly with the PAS, ESS or PDSS-2 estimates. Anxiety and daytime sleepiness receive limited cross-source support at the broad domain level, whereas the nocturnal sleep-disturbance result remains specific to PPMI Online.

Even after adjustment for baseline age and baseline disease duration, the exposed group showed greater mean severity across multiple scales at the start of follow-up. One possible interpretation is that, by the time of enrolment, exposed patients had already accumulated more severe motor and non-motor symptoms than unexposed patients. The “threshold theory” proposes that different neural systems have different thresholds for symptom emergence because of differences in functional reserve^34^. Motor symptoms, for example, may not appear until substantial synaptic loss has occurred, partly because redundant non-dopaminergic inputs can compensate for reduced dopaminergic signalling^35,36^. However, pesticide neurotoxicity may simultaneously impair these compensatory pathways and thereby produce synchronous injury across multiple systems^37,38^. If this were the case, with fewer compensatory inputs, the exposed group might exhibit more severe motor symptoms even at a similar degree of dopaminergic loss than unexposed patients. Systems with less built-in redundancy, such as autonomic and sleep-regulating circuits, may be particularly vulnerable^34^. Pesticide-induced damage to these systems may therefore be translated more directly into overt symptoms. This hypothesis may help explain why, in our data, the baseline offsets for anxiety (PAS: +2.00) and sleep quality (PDSS-2: +2.24) were comparable to or larger than the offset for motor symptoms (MDS-UPDRS Part II: +1.97). However, this interpretation is speculative and cannot be directly tested with our data. Another possibility is that, once the α-synuclein-driven neurodegenerative process has begun, its subsequent rate of progression may be determined primarily by endogenous pathology rather than by prior toxic exposure^39^. Animal data are broadly consistent with this idea: acute MPTP exposure produces a stable rather than progressive Parkinsonian state, and Lewy body-like pathology appears only with continuous administration^40,41^. By analogy, intermittent occupational pesticide exposure might reduce functional reserve without necessarily altering the rate of subsequent neurodegeneration, though this remains a hypothesis.

The “body-first” PD hypothesis^42,43^ offers another lens through which to view our findings. In this model, α-synuclein pathology may originate in the enteric nervous system and ascend through the vagus nerve to the brainstem^44^, so that patients may accumulate widespread non-motor damage before motor symptoms prompt diagnosis^45^. Animal studies have shown that rotenone and paraquat can induce α-synuclein pathology in the enteric nervous system before it appears centrally^46^. The broad non-motor burden we observed in the exposed group, spanning sleep, anxiety, daytime sleepiness, and cognition, is consistent with what would be expected under a body-first phenotype. However, our study did not measure α-synuclein pathology or autonomic biomarkers, so we cannot determine whether the exposed patients actually followed a body-first trajectory. This interpretation should be regarded as hypothesis-generating.

We also found that greater symptom severity at an earlier stage did not predict a faster rate of progression. This finding is consistent with results from several data-driven PD subtype studies^47^. In the PPMI cohort, Zhang et al. clearly showed that the rate of disease progression does not necessarily correspond to baseline severity. Although Subtype II had greater baseline impairment than Subtype I, its motor progression was actually slower. Similarly, in the Oxford Discovery and Tracking Parkinson’s cohorts, Lawton et al. reported that the more severe subtype, characterised by worse motor symptoms, poorer psychological status, and sleep problems, showed only moderate motor progression^24^. In contrast, the fastest progression was observed in another distinct subtype rather than in the subtype with the greatest baseline severity.

The broad non-motor burden observed in the exposed group is also consistent with the multi-pathway nature of pesticide neurotoxicity^48^. Experimental studies have suggested several pathways through which pesticides could affect non-motor function. Organophosphates can impair cholinergic transmission, which is relevant to cognition^49^ and produce lasting serotonergic changes linked to depressive behaviour^50,51^. Paraquat can cause loss of noradrenergic neurons in the locus coeruleus, a key nucleus involved in regulating arousal and anxiety^16,52–54^. These pathways broadly correspond to the non-motor domains in which we observed greater burden in the exposed group.

At the epidemiological level, pesticide exposure has been linked to non-motor outcomes in general populations, including dream-enactment behaviour in the Agricultural Health Study (OR = 1.75)^55^ and depression^56,57^. However, this evidence has come mainly from non-PD populations. Our finding that pesticide-exposed PD patients had higher risks of reaching anxiety and daytime sleepiness milestones extends this evidence to post-diagnostic PD cohorts, though causality cannot be established from observational data.

This study identified a cross-cohort dose–response relationship between the number of pesticide classes involved in combined exposure and motor functional impairment. Participants exposed to four pesticide classes had MDS-UPDRS Part II scores approximately four to five points higher than those exposed to only one class. This finding suggests that, beyond a binary classification of exposure, the diversity of pesticides encountered may also be relevant to motor symptom burden after PD onset. Existing experimental studies provide biological plausibility for this interpretation. In animal models, combined exposure to paraquat and maneb caused greater nigrostriatal damage than either agent alone^58^. More recent mechanistic studies have further suggested that this stronger toxicity may be related to redox imbalance and mitochondrial dysfunction^59^. In addition, Paul et al. integrated epidemiological association analyses of 288 chemicals with toxicity screening in iPSC-derived dopaminergic neurons, identified 53 PD-associated pesticides, and found that pesticide combinations used in agricultural practice were more toxic to human dopaminergic neurons than any single pesticide alone^60^. Real-world pesticide exposure often involves a “cocktail effect” of low-dose pesticide mixtures, which also poses challenges for epidemiological research^61^. In particular, studies focused on specific pesticide compounds may yield inconsistent results because participants are often co-exposed to many different pesticides over the course of their occupational lives^62^. The number of pesticide classes is a crude proxy and cannot be equated with any specific chemical combination. Nonetheless, the dose–response pattern we observed is broadly consistent with experimental evidence that mixed exposures can produce stronger neurotoxicity. Future studies should aim to characterise co-exposure patterns and their potential combined effects in greater detail.

A major strength of this study is the use of two longitudinal online cohorts with repeated motor and non-motor assessments, complemented by an EHR-based triangulation analysis. The online cohorts enabled us to distinguish the symptom level observed when participants entered follow-up from their subsequent annual change and to evaluate clinically meaningful threshold events. The EHR analysis involved different exposure and outcome definitions and should therefore be viewed as directional triangulation rather than direct replication. A second strength is the comprehensive robustness assessment across complementary analytic specifications, which reduced concern that the central descriptive pattern was driven by any single modelling or design choice, although these analyses cannot eliminate unmeasured confounding or selection. Finally, the study considered several symptom domains and exploratory exposure characteristics. The number of reported pesticide classes provides a measure of exposure complexity, but not of absorbed dose or a specific chemical mixture; its graded association with motor burden should therefore be interpreted as exploratory.

This study has several limitations. First, occupational pesticide exposure was assessed retrospectively by self-report and could not be independently verified. The primary contrast classified participants according to any occupational exposure reported across predefined age periods; it did not weight exposure by duration, pesticide class, or intensity. The age-period items supported exploratory analyses of cumulative duration, broad functional classes, and personal protective equipment use, but the questionnaire did not capture application frequency, task-specific intensity, concentration, route, individual products or active ingredients, exact timing relative to PD onset, or the consistency and adequacy of protective practices. The binary exposure variable consequently grouped participants with substantially different exposure profiles and could not quantify absorbed dose or agent-specific effects. Long recall intervals may have introduced additional error, and recall after PD diagnosis may vary with current symptom burden, cognitive status, or awareness of suspected pesticide–PD associations. Such differential recall could shift the estimates in either direction. The association with the number of reported pesticide classes reflects the breadth of reported exposure and does not establish a dose–response relation with absorbed pesticide dose.

Second, both online cohorts enroled participants with prevalent PD at varying stages of disease duration. To enter either cohort, participants had to have survived, remained sufficiently able to participate, and completed online enrolment. Cohort entry was therefore delayed relative to diagnosis and may have been affected by survivor and participation selection. Exposed participants with rapidly progressive disease may consequently have been under-represented, potentially attenuating associations with post-enrolment progression, whereas participation related to symptom burden could have amplified differences observed at cohort entry. Restricting the analyses to participants with less than 5 years of disease duration and applying delayed-entry Cox models yielded similar patterns among those who enroled. However, neither approach could ascertain milestones occurring before enrolment or account for individuals who died, developed severe disability, or never entered the cohorts.

Third, outcome availability, questionnaire structure, and follow-up patterns differed between the cohorts. PAS, ESS and PDSS-2 were assessed only in PPMI Online, precluding same-instrument longitudinal replication of the anxiety and sleep-related findings in Fox Insight. Because TriNetX defines exposure and outcomes using clinical codes, it provides directional triangulation rather than direct validation of the scale-based associations observed in the online cohorts. Both online cohorts recruited participants who were willing and able to complete web-based questionnaires, which may limit generalisability to individuals with advanced or rapidly fatal disease, those living in institutional settings, and those with limited digital access.

Fourth, residual confounding cannot be excluded. Agricultural or rural lifestyle, occupation-specific co-exposures, socioeconomic position, access to healthcare, PD subtype, and genetic susceptibility were not fully captured. Treatment-related factors are an additional source of uncertainty. Anti-parkinsonian medication was omitted from the primary models because treatment initiation and intensification are partly driven by prior symptom burden; treatment may therefore be both a consequence of earlier disease severity and a determinant of subsequently measured symptoms. Additional adjustment for available indicators of overall medication use, individual drug classes, and DBS status did not materially change the estimates. However, neither cohort recorded the dose, dosing frequency, or units required to calculate levodopa-equivalent daily dose (LEDD). Consequently, residual treatment-related confounding by treatment intensity may remain despite adjustment for overall medication use, individual drug classes, and DBS status. Binary treatment indicators also could not capture adherence, treatment escalation, timing relative to outcome assessment, motor state at assessment, access to specialist care, or therapeutic response. The estimates should therefore be interpreted as associations with symptom burden under usual care rather than with untreated disease severity.

Fifth, 1:1 PSM narrowed the primary comparison by selecting unexposed participants whose measured covariate distributions resembled those of the exposed group. Nearly all exposed participants were retained, whereas most eligible unexposed participants were excluded. The matched estimates are therefore most naturally interpreted as applying to exposed participants within the region of adequate common support, rather than as population-average associations for the entire eligible PD cohort. Retained and excluded unexposed participants were similar in age and disease duration and showed only small differences in baseline motor severity, although they differed in sex and, to a lesser extent, education and smoking history. Average-treatment-effect weighting of the full eligible cohorts yielded directionally concordant estimates, suggesting that the overall pattern was not restricted to the subset of unexposed participants selected through matching. Nevertheless, the matched and weighted analyses target different populations, and neither can address unmeasured confounding or selection into the online cohorts.

Occupational pesticide exposure was associated with greater symptom levels at entry into observation, most clearly in PPMI Online, with partial support for motor and cognition-related outcomes in Fox Insight. We found no consistent evidence of faster post-enrolment change. The PPMI-only anxiety and sleep-scale findings require confirmation in independent longitudinal cohorts using comparable exposure assessment and repeated non-motor measures. These observational findings support further investigation of environmental contributions to heterogeneity in PD symptom burden but do not establish causality or untreated disease progression.

## Methods

### Study overview

This study used three complementary data sources to examine the associations between occupational pesticide exposure and motor and non-motor symptom burden in PD. We used PPMI Online and Fox Insight as the primary longitudinal cohorts to analyze symptom trajectories, milestone events, and exposure-detail variables. We also conducted a triangulation analysis in the TriNetX Global Collaborative Network, a large federated EHR database, to examine whether the overall pattern of associations was directionally consistent in a data source with different exposure definitions and outcome measures. Because the TriNetX analysis used diagnosis-coded exposure definitions and outcomes, we treated it as a cross-source triangulation component rather than as a direct replication of the longitudinal analyses.

### Study populations (PPMI Online and Fox Insight)

For the longitudinal analyses, we used Fox Insight and PPMI Online, two online longitudinal observational studies sponsored by The Michael J. Fox Foundation that enrol individuals with PD as well as non-PD controls^31,32^. These cohorts prospectively collect self-reported patient outcomes through regular online questionnaires, with follow-up assessments conducted approximately every 3 to 6 months. For Fox Insight, the informed consent process and study protocol were approved by the WCG Institutional Review Board (IRB#: 120160179; legacy IRB#: 14–236; sponsor protocol number: 1; study title: Fox Insight), and all participants provided informed consent online through the Fox Insight website^32^. The PPMI study was conducted in accordance with the Declaration of Helsinki and Good Clinical Practice guidelines, with approval from the local ethics committees at each participating centre, and all participants provided written informed consent before enrolment^31^. As a secondary analysis of publicly available de-identified data, the present study was approved by the Ethics Committee of Charité–Universitätsmedizin Berlin (Ethikkommission der Charité–Universitätsmedizin Berlin).

For motor symptoms, both cohorts performed longitudinal assessments using the Movement Disorder Society–Unified Parkinson’s Disease Rating Scale Part II (MDS-UPDRS Part II). For non-motor symptoms, PPMI Online included longitudinal assessments of cognition-related daily functioning using the Penn Parkinson’s Daily Activities Questionnaire-15 (PDAQ-15), depression using the Geriatric Depression Scale-15 (GDS-15), anxiety using the Parkinson Anxiety Scale (PAS), and sleep using the Epworth Sleepiness Scale (ESS) and the Parkinson’s Disease Sleep Scale-2 (PDSS-2). Fox Insight included longitudinal assessments of cognition-related daily functioning using the PDAQ-15 and depression using the GDS-15.

Participants in both cohorts completed the Parkinson’s Disease Risk Factor Questionnaire–Updated (PD-RFQ-U), which collected information on environmental exposures and lifestyle factors, including a detailed history of occupational pesticide exposure. In Fox Insight, the questionnaire was administered once at enrolment. In PPMI Online, it was administered at up to two follow-up visits. For participants with two completed records, we selected the version with more complete responses for analysis; when both records showed the same level of completeness, we retained the more recent record.

From these two cohorts, we identified patients with PD and defined the first symptom assessment as the start of longitudinal follow-up. Participants were eligible if they met all of the following criteria: (1) age at diagnosis older than 30 years and disease duration of less than 10 years at the start of follow-up; (2) at least one study outcome scale with a calculable baseline total score at the first visit; (3) clearly recorded pesticide exposure status; and (4) clearly recorded or derivable age at diagnosis, sex, and disease duration at baseline. After screening, 7190 patients from PPMI Online and 2298 patients from Fox Insight were included in the study. Their mean baseline ages were 67.49 ± 8.84 years and 64.11 ± 9.10 years, respectively, and their mean baseline disease durations were 2.76 ± 2.44 years and 3.20 ± 2.56 years, respectively.

These criteria were selected to define a clinically comparable early-to-mid-stage PD population while retaining a plausible adult occupational-exposure window. Excluding participants diagnosed at 30 years of age or younger reduced the inclusion of very young-onset disease, whereas restricting baseline disease duration to less than 10 years limited heterogeneity in disease stage at cohort entry. Requiring an ascertainable exposure status, the demographic information needed to construct the analytic cohort, and at least one calculable baseline outcome ensured that each participant contributed interpretable exposure and outcome information. The implications of these restrictions for prevalent-case selection and generalisability are considered in the Discussion.

### Determination of pesticide exposure

Information on pesticide exposure and related exposure characteristics was obtained from the PD-RFQ-U. The overall questionnaire content was largely consistent across the two cohorts, although several items differed slightly in design. In PPMI Online, the use of PPE was recorded separately for each age period, whereas in Fox Insight, it was assessed as a summary item. In addition, exposure history was categorised into nine age periods in PPMI Online and six age periods in Fox Insight. The original questionnaires used in the two cohorts are publicly available on the PPMI and Fox Insight websites, respectively.

Occupational pesticide exposure was ascertained from age-period-specific items in the PD-RFQ-U. Participants who reported occupational pesticide exposure during at least one age period were classified as exposed. Those who reported no exposure across all ascertainable age periods were classified as unexposed, whereas participants whose exposure status could not be determined were excluded. The questionnaire covered nine age periods in PPMI Online and six in Fox Insight and additionally assessed cumulative exposure duration, broad functional classes of pesticides, and personal protective equipment use. The age-period-based classification was compared with the lifetime-summary item and showed high concordance. In PPMI Online, no participant who answered “yes” to the lifetime-summary item was classified as unexposed. However, 39 of 1065 exposed participants were identified through a positive age-period response despite not having a positive lifetime-summary response. Exploratory variables describing exposure characteristics were derived from the same age-period-specific items.

Regarding more detailed exposure characteristics, both cohorts collected information on exposure duration, the functional classes of pesticides encountered, specific pesticide types, and PPE use. Both cohorts included herbicides, fungicides, insecticides, and fumigants, whereas Fox Insight additionally recorded rodenticide exposure. Based on these questionnaire items, we derived the following exploratory exposure variables: (1) cumulative exposure duration, defined as the sum of exposure years across all age periods; (2) the number of pesticide functional classes involved in combined exposure, ranging from 0 to 4 in PPMI Online and from 0 to 5 in Fox Insight; (3) binary indicators for exposure to each pesticide functional class; and (4) PPE use.

### Determination of PD-related outcomes

The longitudinal outcomes in this study were classified into two categories: continuous scale scores and milestone events, which were analyzed using linear mixed-effects models (LMMs) and Cox regression models, respectively.

For continuous outcomes, motor symptoms were assessed using the MDS–UPDRS Part II (score range, 0–52), with higher scores indicating worse motor function. The non-motor symptom scales included the PDAQ-15 (0–60; higher scores indicate better cognitive function), GDS-15 (0–15; higher scores indicate more severe depressive symptoms), ESS (0–24; higher scores indicate greater daytime sleepiness), PAS (0–48; higher scores indicate more severe anxiety symptoms), and PDSS-2 (0–60; higher scores indicate more severe sleep disturbance). In the Fox Insight cohort, only three scales were collected longitudinally: MDS–UPDRS Part II, PDAQ-15 and GDS-15.

For milestone events, we defined one clinically meaningful threshold of functional impairment for each of the five symptom domains based on published validation studies: moderate motor impairment (MDS–UPDRS Part II >12)^63^, dementia-level cognitive impairment (PDAQ-15 <37)^64^, clinically significant depression (GDS >5)^65^, excessive daytime sleepiness (ESS >10)^66^, and anxiety disorder (PAS >13)^67^. For each milestone, participants were included in the corresponding risk set only if they had not reached the relevant threshold at baseline.

For item-level missing data, we allowed up to two missing items for the MDS–UPDRS Part II (13 items) and prorated the total score accordingly^68^. For the GDS-15, we allowed up to five missing items and similarly prorated the total score^69^. For the PDAQ-15, PAS and PDSS-2, total scores were prorated when at least 80% of items had been completed; otherwise, the total score for that visit was considered missing^70,71^.

### Determination of covariates and propensity score matching

To reduce confounding, we first constructed a directed acyclic graph (DAG) to define the hypothesised causal framework linking pesticide exposure to the progression of motor and non-motor symptoms in PD (Supplementary Fig. 1). Based on this framework, candidate covariates were classified as confounders, mediators, or instrumental variables, and only confounders were included in the adjusted analyses. The confounders selected for the propensity score model were baseline age, sex, disease duration, race and ethnicity, family history of PD, years of education, smoking history, and coffee consumption history^8,72–75^. The detailed selection process is presented in Supplementary Fig. 1.

Smoking history and coffee consumption history were both obtained from the PD-RFQ-U. Regular smoking was defined as the use of any tobacco product at a frequency of at least one cigarette per day, or an equivalent amount, for 6 months or longer at any time during life. Regular coffee consumption was defined as the intake of caffeinated coffee at least once per week for 6 months or longer at any time during life.

We then estimated propensity scores using logistic regression, with pesticide exposure status as the dependent variable and all of the above confounders as independent variables. Participants were matched 1:1 by nearest-neighbour matching without replacement, using a caliper width equal to 0.2 times the standard deviation of the logit of the propensity score. Matching was performed using the MatchIt package in R. Covariate balance after matching was assessed using standardised mean differences (SMDs), and an absolute SMD of less than 0.1 was considered acceptable. After matching, 2118 patients remained in the PPMI Online cohort, and 800 remained in the Fox Insight cohort.

Because caliper-based nearest-neighbour matching does not produce exact matches on every covariate, the primary outcome models additionally included baseline age, sex and disease duration, which are strong prognostic factors and were also included in the propensity-score model. This post-matching adjustment was used to account for any residual imbalance and improve precision rather than to redefine the matched comparison. Missingness and explicit non-response categories for all propensity-score covariates were quantified in the full eligible cohorts (Supplementary Table 14). The primary propensity-score model retained an explicit missing or non-response category where applicable to avoid substantial complete-case exclusion. We assessed this choice by repeating propensity-score estimation, matching, and outcome modelling in complete cases and after multiple imputation by chained equations, as described under sensitivity and diagnostic analyses.

For each outcome scale, the analysis cohort was defined as the intersection of the matched skeleton cohort and the participants with a valid baseline total score for that scale. Before conducting the longitudinal analysis for each outcome, we recalculated SMDs within the corresponding analysis subset to confirm that covariate balance remained adequate (Supplementary Table 1). For subsets that did not meet the balance criterion, we repeated the propensity score matching procedure and conducted sensitivity analyses in the rematched cohort to assess the robustness of the findings.

In the exploratory analyses of exposure-detail variables, we further constructed nested subcohorts within the exposed group. Because the proportion of missing data increased with the level of detail in these variables, participants without the relevant information were excluded from the corresponding analyses. Before each subcohort analysis, we assessed differences between included and excluded participants in baseline covariates and baseline score distributions using standardised mean differences, with an absolute SMD of less than 0.1 considered acceptable.

### Statistical analysis

The time scale for the longitudinal models was defined as the follow-up time in years since the first symptom assessment. In the LMMs, the dependent variable was the total score for each scale. Fixed effects included time, exposure status, baseline age, sex, and disease duration at baseline. An interaction term between exposure status and time was added to assess the association of exposure with the rate of progression. Random effects were specified at the participant level, with both random intercepts and random slopes for time to capture between-individual heterogeneity in baseline symptom severity and progression rate.

For the five prespecified milestone events, we fitted multivariable Cox proportional hazards models in the matched cohorts, restricted to participants who had not reached the corresponding threshold at baseline. Time zero was defined as the date of the baseline assessment, and event time was defined as the time in years to the first visit at which the corresponding threshold was reached. Participants who did not experience the milestone event were censored at their last valid assessment. Pesticide exposure status was included as the primary predictor, and baseline age, sex, and disease duration were further adjusted for to account for residual confounding that might remain after propensity score matching.

In addition, we performed exploratory analyses of exposure-detail variables, including cumulative exposure duration, the number of pesticide functional classes involved in combined exposure, exposure to each specific pesticide functional class, and the use of personal protective equipment. These analyses were conducted within the exposed group of the matched cohort using LMMs. Specifically, the binary exposure status variable in the primary model was replaced with the corresponding exposure-detail variable, while the remaining model structure was kept unchanged.

The primary analyses involved repeated applications of LMMs across six outcome scales and Cox regression models across five milestone events, yielding six and five core hypothesis tests, respectively. We therefore applied the Benjamini–Hochberg procedure separately to control the false discovery rate (FDR) for the LMM and Cox analyses, and *q* values of less than 0.05 were considered statistically significant. *P* values for covariates and exploratory analyses were not adjusted for multiple comparisons, and a two-sided *p* value of less than 0.05 was considered statistically significant.

All analyses were performed in R, version 4.5.2^76^. The main packages used were lme4 for LMMs^77^; survival and survminer for Cox regression and Kaplan–Meier curve plotting^78,79^; MatchIt for propensity score matching^80^; cobalt for covariate balance diagnostics^81^; and ggplot2 for visualisation^82^. Except for the FDR correction procedures described above, a two-sided *p* value of less than 0.05 was considered statistically significant. Results from the LMMs are presented as regression coefficients with 95% confidence intervals, and results from the Cox models are presented as hazard ratios (HRs) with 95% confidence intervals.

### Sensitivity and diagnostic analyses

We conducted sensitivity and diagnostic analyses to evaluate whether the primary findings were robust to treatment-related factors, the matched-cohort design, missing covariate data, prevalent-cohort entry, milestone definition and differential follow-up. Unless otherwise specified, each analysis altered only the factor under evaluation and otherwise retained the propensity-score–matched cohorts and outcome-model specifications used in the primary analyses.

First, because anti-parkinsonian medication was treated as a downstream mediator in the causal framework and was therefore omitted from the primary model, we assessed whether treatment status could account for the observed differences in symptom severity. We refitted the mixed-effects models with additional adjustment for either baseline or time-varying use of any anti-parkinsonian medication. Visit-level medication status was derived from the overall current-medication indicator and the brand-level medication checkboxes. Medication records were linked to symptom assessments by visit identifier in PPMI Online and to the nearest preceding medication record within one year in Fox Insight; no future medication information was used. Levodopa-equivalent daily dose could not be calculated because neither cohort recorded medication dose, dosing frequency, or units. Additional model specifications included adjustment for time-varying use of levodopa, dopamine agonists, monoamine oxidase B inhibitors, catechol-*O*-methyltransferase inhibitors, amantadine, and anticholinergics and, separately, for ever having undergone deep brain stimulation (DBS). We also repeated the analyses after excluding participants with any DBS record. DBS status was obtained from the PPMI Online treatment items and the Fox Insight procedure questionnaire.

Second, to assess the influence of 1:1 matching while retaining the full eligible cohorts, we re-estimated the outcome models using stabilised inverse-probability-of-treatment weights (IPTW) targeting the average-treatment effect and derived from the same propensity-score model used for matching. Weights were trimmed at the 1st and 99th percentiles. Weighting diagnostics included the weight distribution, effective sample sizes, and post-weighting standardised mean differences. Continuous outcomes were analyzed using weighted mixed-effects models, whereas milestone outcomes were analyzed using weighted Cox models with robust standard errors.

Third, to evaluate the explicit missing or non-response categories used for propensity-score covariates in the primary analysis, we repeated propensity-score estimation, matching, and outcome modelling in complete cases and after multiple imputation by chained equations. The complete-case analysis excluded participants without the covariate information required for the corresponding propensity-score specification. For the multiple-imputation analysis, we generated 20 imputed datasets; pesticide exposure status was not imputed. Propensity scores were re-estimated, and matching and outcome modelling were performed separately within each imputed dataset. Fixed-effect coefficients from the mixed-effects models and log hazard ratios from the Cox models were then pooled using Rubin’s rules.

Fourth, to assess the representativeness of the matched cohorts, we quantified the proportion of eligible participants retained after matching separately by cohort and exposure status. We also compared retained and excluded unexposed participants using standardised mean differences for demographic characteristics, lifestyle factors, disease duration, and baseline motor severity.

Fifth, to examine sensitivity to prevalent-cohort entry and left truncation, we repeated the primary mixed-effects and Cox analyses after restricting each cohort to participants with less than 5 years of disease duration at baseline. For milestone outcomes, we additionally fitted delayed-entry Cox models using time since PD diagnosis as the analysis time scale. Participants entered the risk set at their disease duration at the baseline assessment and were followed until milestone occurrence or censoring.

Sixth, because earlier milestone attainment could reflect greater symptom burden at baseline rather than more rapid post-baseline worsening, we refitted each Cox-model with additional adjustment for the corresponding baseline scale score. Because the baseline score may partly reflect an earlier effect of pesticide exposure and therefore lie on the causal pathway, these models were treated as explanatory sensitivity analyses rather than as the primary causal specification.

Seventh, to assess differential follow-up, we compared the number of completed assessments, mean follow-up duration, the proportion of participants followed for at least 2 years, and the proportion contributing only a baseline assessment between exposure groups in the matched MDS-UPDRS Part II cohort. Differences in follow-up duration were evaluated using a linear model, and baseline-only participation was evaluated using a logistic model, with adjustment for age, sex, disease duration at baseline and baseline MDS-UPDRS Part II score.

For all refitted mixed-effects models, we extracted the exposure main effect and the exposure-by-time interaction with 95% confidence intervals to distinguish differences in symptom level from differences in progression rate. Group-specific annual progression slopes were derived from the primary models: the slope in the unexposed group corresponded to the time coefficient, the slope in the exposed group corresponded to the sum of the time and exposure-by-time coefficients, and their difference corresponded to the exposure-by-time interaction. Confidence intervals for the exposed-group slopes were derived from the model covariance matrix.

For milestone outcomes, treatment-related covariates were entered as baseline status. The parallel Cox-model sensitivity analyses included adjustment for baseline use of any anti-parkinsonian medication; full-cohort IPTW with robust standard errors; complete-case and multiply imputed analyses with propensity-score rematching; adjustment for baseline use of levodopa, dopamine agonists, and monoamine oxidase B inhibitors; adjustment for DBS status; exclusion of participants with any DBS record; and restriction to participants with less than 5 years of disease duration at baseline. These analyses were complemented by the baseline score-adjusted and delayed-entry models described above. Results are presented in Supplementary Tables 12–22 and Supplementary Figs. 4 and 5.

### Cross-source triangulation in an EHR database: data source and study population

To examine whether the overall pattern of findings was also seen in a different type of data source, we conducted an independent triangulation analysis using the TriNetX Global Collaborative Network. TriNetX is a federated, de-identified EHR research network, and the network used in this analysis included 171 healthcare organizations. The analysis was performed on the TriNetX platform on April 16, 2026.

The study population consisted of patients with a recorded diagnosis of PD (ICD-10-CM: G20). The date of the first recorded PD diagnosis was defined as the index event, and patients whose index event occurred more than 20 years before the analysis date were excluded. The exposed cohort was defined as patients with PD who also had records of toxic effects of pesticides (T60) or contact with and suspected exposure to pesticides (Z77.098). The control cohort included patients with PD who had neither of these exposure-related codes.

Because the ICD coding system does not provide a code specific to occupational pesticide exposure, we used this combination of codes to identify, as closely as possible, clinical records suggestive of pesticide exposure. In contrast to the longitudinal cohort analyses, which defined exposure on the basis of self-reported lifetime occupational pesticide exposure in the PD-RFQ-U questionnaire, the TriNetX analysis was more likely to capture pesticide toxic events or related exposures documented in the healthcare system. It therefore likely represents a narrower and potentially more severely exposed subgroup.

### Determination of diagnosis-coded outcomes in the EHR database

For each outcome, follow-up began on the index date and continued until the first occurrence of the corresponding outcome or loss to follow-up, whichever occurred first. Outcomes were defined as the first occurrence, after the index event, of any of the following diagnoses: motor impairment (Z74.0–Z74.3, including reduced mobility and the need for care or supervision), dementia (F02.80 and F02.81), depression (F06.31, F06.32, F32, and F33), anxiety (F06.4, F41.0, F41.1, and F41.9), and sleep disturbance (G47.1 and R40.0). The full ICD-10 code definitions are listed in Supplementary Table 8.

### Propensity score matching and statistical analysis in the EHR database

Propensity scores were estimated using logistic regression. Participants were matched 1:1 by nearest-neighbour matching without replacement, using a caliper width equal to 0.2 standard deviations of the logit of the propensity score. Matching covariates included age, sex, race, ethnicity, personal history of nicotine dependence (Z87.891), and alcohol dependence (F10.2). After matching, 755 patients remained in each group. Covariate balance was assessed using standardised mean differences, with an absolute standardised mean difference of less than 0.1 considered acceptable.

We used the TriNetX Compare Outcomes module to compare the cumulative risks of the five diagnosis-coded outcomes between the matched exposed and unexposed groups. Associations were summarised using risk differences, risk ratios, and odds ratios with corresponding 95% confidence intervals. A two-sided *p* value of less than 0.05 was considered statistically significant. Because this analysis was intended to provide directional triangulation rather than confirmatory hypothesis testing, no adjustment was made for multiple comparisons across the five outcomes.

We additionally conducted an exploratory time-to-event analysis for the motor function-related outcome using a Cox proportional hazards model. The outcome was the first occurrence of any of the motor impairment codes Z74.0–Z74.3. This model was fitted in the full unmatched analysis cohort, comprising 755 exposed and 493,163 unexposed patients. Pesticide exposure was entered as the primary predictor, with adjustment for sex, age at the index event, and baseline diagnoses of dementia (F02.80), mood disorder (F06.3), depressive episode (F32), anxiety disorder (F41) and nicotine dependence (F17).

All TriNetX analyses were performed within the federated platform, which returned aggregate statistical results. The investigators did not directly access or download patient-level raw data. Because the EHR outcomes were defined using diagnosis codes and did not fully correspond to the threshold-based outcomes in the longitudinal cohorts, the TriNetX component was treated as complementary real-world evidence for directional triangulation rather than as an external validation. Detailed ICD-10 definitions, baseline characteristics, and complete association estimates are provided in Supplementary Tables 8–11.

### Ethics statement

The data used in this study were obtained from two publicly available research resources established by the Michael J. Fox Foundation, PPMI Online and Fox Insight, together with de-identified electronic health record data from the TriNetX Global Collaborative Network. The Fox Insight study protocol and informed consent procedures were approved by the WCG Institutional Review Board (IRB# 120160179; Legacy IRB# 14-236; Sponsor Protocol Number: 1; Study Title: Fox Insight), and all participants provided electronic informed consent through the Fox Insight website. The PPMI Online study was conducted in accordance with the Declaration of Helsinki and Good Clinical Practice guidelines, with approval from the local ethics committees at all participating centres, and all participants provided written informed consent before enrolment. For the TriNetX analysis, the study used retrospective, de-identified electronic health record data and involved no direct contact with participants. It therefore met the exemption criteria under the Health Insurance Portability and Accountability Act (HIPAA) and did not require informed consent. Qualified experts had previously evaluated the TriNetX dataset to confirm compliance with HIPAA de-identification standards under §164.514(a) and §164.514(b)(1), and the most recent de-identification assessment was completed in December 2020. As a secondary analysis of publicly available, de-identified data, the present study was approved by the Ethics Committee of Charité–Universitätsmedizin Berlin.

Clinical trial registration: Not applicable. This study was a retrospective observational cohort study based on secondary analysis of previously collected de-identified data from PPMI Online, Fox Insight, and TriNetX. No participants were prospectively assigned by the investigators to any health-related intervention, treatment, exposure or management strategy.

## Supplementary information

Supplementary information

## Data Availability

The data used in this study were downloaded from the Fox Insight database on March 2, 2026, and from the Parkinson’s Progression Markers Initiative database (RRID: SCR_006431) on July 14, 2025. The PPMI Online data analyzed in this study were accessed as part of the PPMI sub-study through the same PPMI data portal. Updated information on both studies is available on the Fox Insight and PPMI websites. Researchers may apply for data access by signing a Data Use Agreement. The TriNetX analyses were conducted within the federated TriNetX Global Collaborative Network; patient-level data were not downloaded or directly accessible to the investigators. Aggregate results supporting these analyses are provided in the Article and Supplementary Information. This study did not generate any new raw data.

## Code availability

The R code used for data processing and statistical analyses of the PPMI Online and Fox Insight cohorts is available from the corresponding author upon reasonable request. The TriNetX analyses were performed using the platform’s built-in analytical tools.
