# Supplementary material for: Pesticide exposure and multidomain symptom burden in Parkinson’s disease: a longitudinal triangulation study
Source: NPJ Parkinsons Dis. 2026 Aug 5;12:185. doi: 10.1038/s41531-026-01510-9 (PMC13443583; doi:10.1038/s41531-026-01510-9)
Supplement: Supplementary file 1 [file 41531_2026_1510_MOESM1_ESM.pdf]

Supplementary Information:

Pesticide Exposure and Multidomain Symptom Burden in Parkinson's Disease: A Longitudinal Triangulation Study

Zhengjie Yang [1], Shijv Shen [1], Saskia Preissner [2], Robert Preissner [1,3,*]

1 Institute of Physiology, Charité–Universitätsmedizin Berlin, Philippstr. 12, 10115 Berlin, Germany

2 Department of Oral and Maxillofacial Surgery, Charité–Universitätsmedizin Berlin, Augustenburger Platz 1, 13353 Berlin, Germany

3 Department of Congenital Heart Disease and Pediatric Cardiology, University Hospital of Schleswig-Holstein, Campus Kiel, Germany

* Correspondence: robert.preissner@charite.de

Table of Contents

Supplementary Figure 1. Directed acyclic graph (DAG) illustrating the hypothesized causal framework linking pesticide exposure to PD symptom progression. 4
Supplementary Figure 2. Model-predicted trajectories of motor and non-motor symptom progression by pesticide exposure status in the Fox Insight cohort. 6
Supplementary Figure 3. Kaplan–Meier event-free survival curves for clinical milestone events by pesticide exposure status in the Fox Insight cohort. 7
Supplementary Figure 4. Robustness of the exposure–severity and exposure–progression associations across all sensitivity analyses. 8
Supplementary Figure 5. Milestone (Cox) hazard ratios for pesticide exposure across all sensitivity analyses. 9
Supplementary Table 1. Covariate Balance Between Exposed and Unexposed Groups Within Each Scale-Specific Analysis Subset After Propensity Score Matching. 10
Supplementary Table 2. Linear Mixed-Effects Model Estimates for Overall Symptom Progression Across Scales in the PPMI Online and Fox Insight Cohorts. 16
Supplementary Table 3. Linear Mixed-Effects Model Estimates for the Association of Pesticide Exposure With Symptom Severity and Progression Rate Across Scales in the PPMI Online and Fox Insight Cohorts. 18
Supplementary Table 4. Cox proportional hazards model results for pesticide exposure and PD-related endpoints (PPMI and FoxInsight). 20
Supplementary Table 5. Linear Mixed-Effects Model Estimates for the Dose–Response Association Between Number of Co-Exposed Pesticide Functional Classes and Symptom Severity and Progression Rate in the PPMI Online and Fox Insight Cohorts. 22
Supplementary Table 6. Linear Mixed-Effects Model Estimates for the Association of Cumulative Exposure Duration and Personal Protective Equipment Use With Symptom Severity and Progression Rate in the PPMI Online and Fox Insight Cohorts. 25
Supplementary Table 7. Linear Mixed-Effects Model Estimates for the Association of Specific Pesticide Functional Classes With Symptom Severity and Progression Rate in the PPMI Online and Fox Insight Cohorts. 28
Supplementary Table 8. ICD-10 Outcome Definitions for the TriNetX Triangulation Analysis. 32
Supplementary Table 9. Baseline characteristics of PD patients with and without ICD-coded pesticide exposure before and after propensity score matching in TriNetX analysis. 33
Supplementary Table 10. Measure of Association Analysis for Five Symptom Domains After Propensity Score Matching. 34
Supplementary Table 11. Cox Proportional Hazards Model for the Motor Function Milestone Event. 35
Supplementary Table 12. Pesticide-exposure associations before and after adjustment for anti-parkinsonian medication. 36
Supplementary Table 13. Inverse-probability-of-treatment-weighting (IPTW) analysis of the full cohorts versus the matched analysis. 38
Supplementary Table 14. Missing-data sensitivity analyses. 40
Supplementary Table 15. Representativeness of the matched cohort (matched vs. excluded participants). 42

Supplementary Table 16. Sensitivity to left truncation: patients with less than five years' baseline disease duration. 44
Supplementary Table 17. Milestone (Cox) sensitivity: adjustment for baseline severity and delayed-entry models. 46
Supplementary Table 18. Follow-up and attrition by exposure group (matched UPDRS-II cohort). 47
Supplementary Table 19. Exposure×time (progression) term across all sensitivity analyses. 48
Supplementary Table 20. Drug-class and deep-brain-stimulation (DBS) sensitivity analyses. 49
Supplementary Table 21. Annual progression slope within each exposure group and the slope difference (exposure×time interaction). 51
Supplementary Table 22. Milestone (Cox) hazard ratios for pesticide exposure across all sensitivity analyses. 52
Supplementary Note 1. Additional Results for Other Exposure Characteristics 53

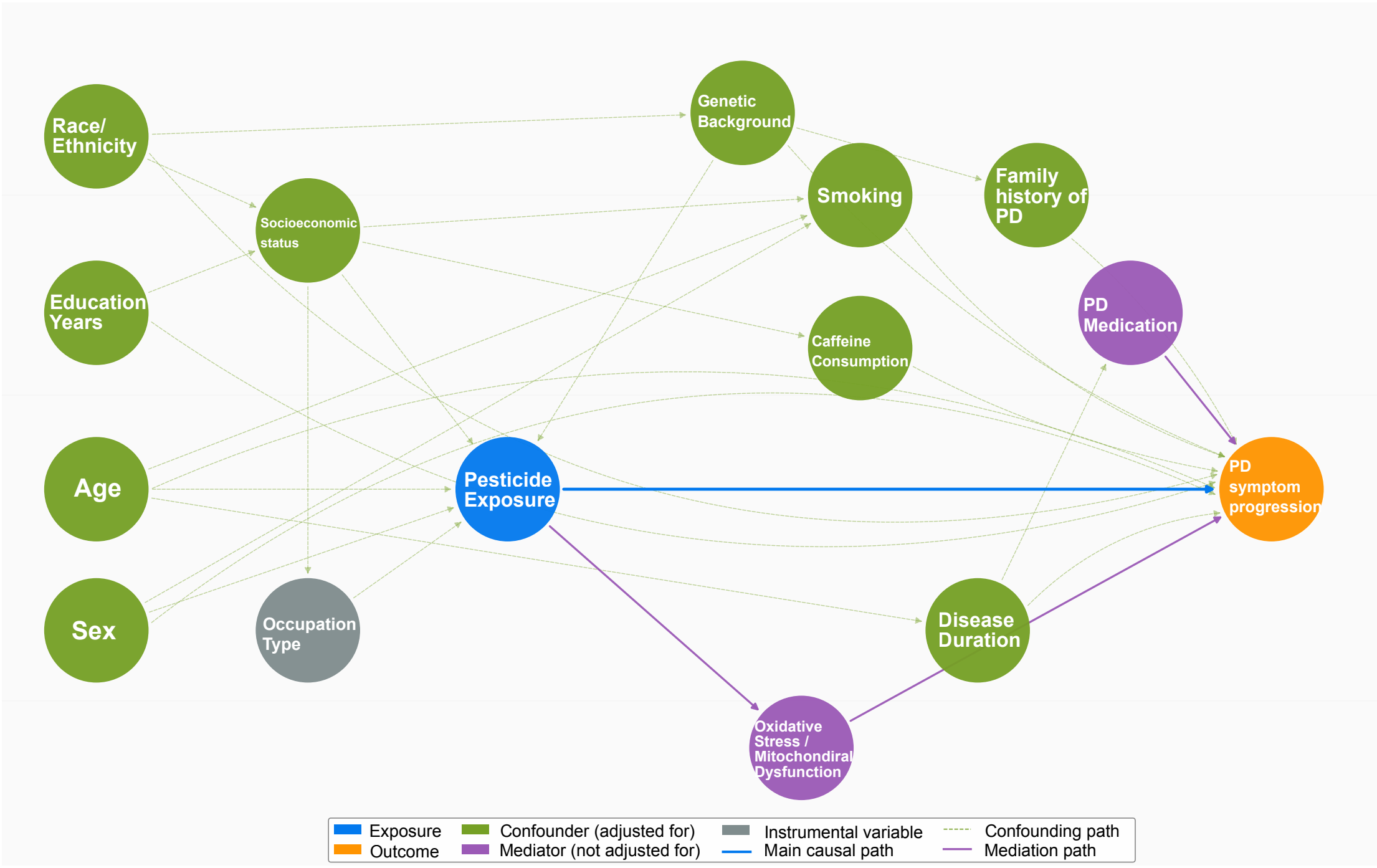

Supplementary Figure 1. Directed acyclic graph (DAG) illustrating the hypothesized causal framework linking pesticide exposure to PD symptom progression. The DAG depicts the assumed relationships among the exposure (blue: pesticide exposure), the outcome (orange: PD symptom progression), confounders (green: adjusted for in the propensity score model), mediators (purple: not adjusted for), and an upstream determinant of exposure (grey: occupation type). The main causal path from pesticide exposure to PD symptom progression is shown as a solid blue arrow. Confounding paths (dashed green lines) represent non-causal associations that may bias the exposure–outcome relationship if unadjusted. Confounders selected for adjustment included baseline age, sex, race/ethnicity, education years, smoking history, caffeine consumption history, family history of PD, and disease duration at baseline. Socioeconomic status and genetic background were identified as confounders but could not be directly measured in the available data; their potential influence was partially captured through proxy variables (education years, race/ethnicity, and family history of PD). Mediators not adjusted for included oxidative stress/mitochondrial dysfunction (a hypothesized biological mechanism on the causal pathway) and PD medication (which may be influenced by both exposure status and disease severity). Occupation type was treated as an upstream determinant of occupational pesticide exposure rather than as an adjustment variable, because detailed occupation categories were not harmonized across cohorts and may also capture socioeconomic and co-exposure pathways. This DAG informed the selection of covariates for propensity score matching and multivariable adjustment in the primary analyses.

The propensity score model included the following confounders: (1) baseline age and sex, as both may influence the likelihood of occupational pesticide exposure and the rate of disease progression; (2) race and ethnicity, as upstream determinants of socioeconomic position and genetic background; (3) years of education, which may affect occupational choice and independently influence non-motor symptom progression through cognitive reserve; (4) baseline disease duration, which is related to both cumulative symptom burden and the duration of the potential exposure window; (5) family history of PD, used as a proxy for genetic susceptibility; (6) regular smoking history,

which has been associated with disease progression and is also shaped by upstream factors such as socioeconomic position and age; and (7) regular coffee consumption, because caffeinated beverage intake has been associated with motor severity in PD and shares upstream socioeconomic and lifestyle determinants with pesticide exposure. Variables on the causal pathway between exposure and outcome, including biological mediators such as oxidative stress and mitochondrial dysfunction, as well as PD medication use, were excluded to avoid overadjustment.

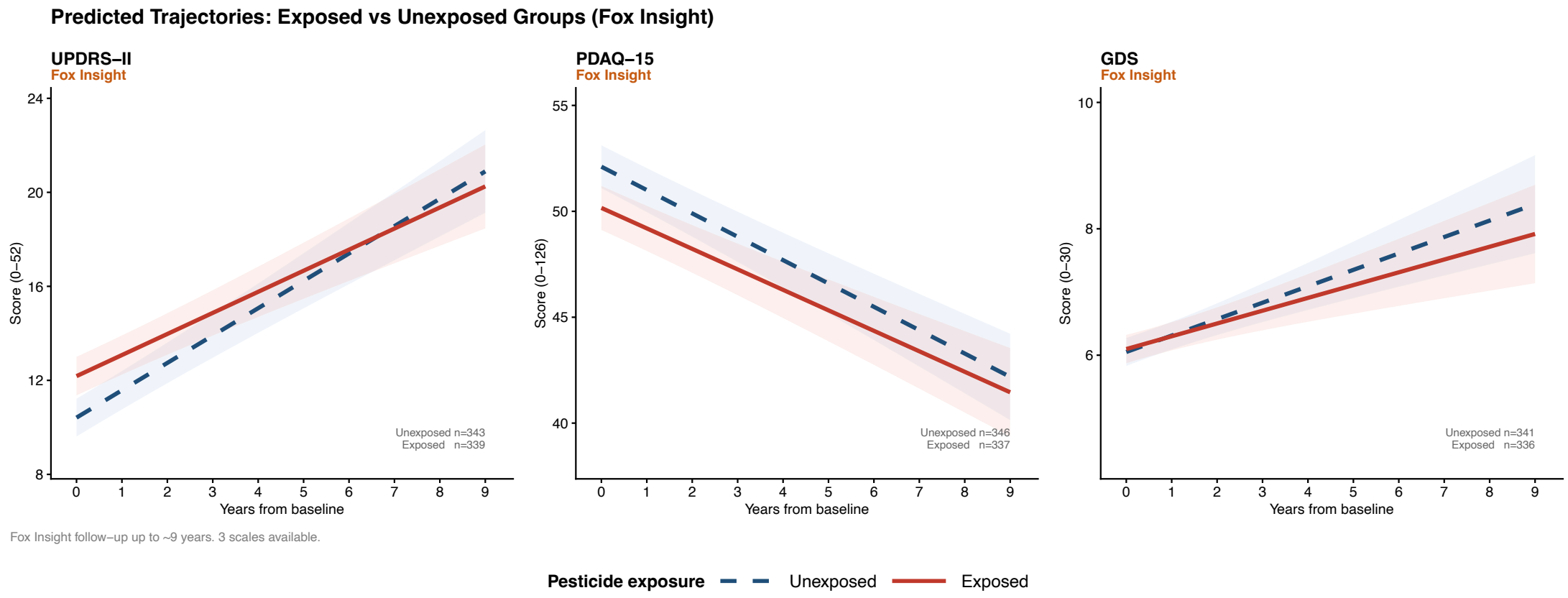

Supplementary Figure 2. Model-predicted trajectories of motor and non-motor symptom progression by pesticide exposure status in the Fox Insight cohort. Predicted mean trajectories for the pesticide-exposed (solid red lines) and unexposed (dashed blue lines) groups with 95% confidence bands, derived from linear mixed-effects models adjusted for baseline age, sex, and disease duration at baseline, with random intercepts and random slopes for time per patient. Three scales were available in Fox Insight with follow-up extending to approximately 9 years: UPDRS-II (higher scores indicate worse motor function), PDAQ-15 (higher scores indicate better cognition-related daily functioning), and GDS (higher scores indicate more severe depressive symptoms). Sample sizes for each scale are indicated within the panels. UPDRS II, Movement Disorder Society–Unified Parkinson's Disease Rating Scale Part II; PDAQ-15, Penn Parkinson's Daily Activities Questionnaire-15; GDS, Geriatric Depression Scale.

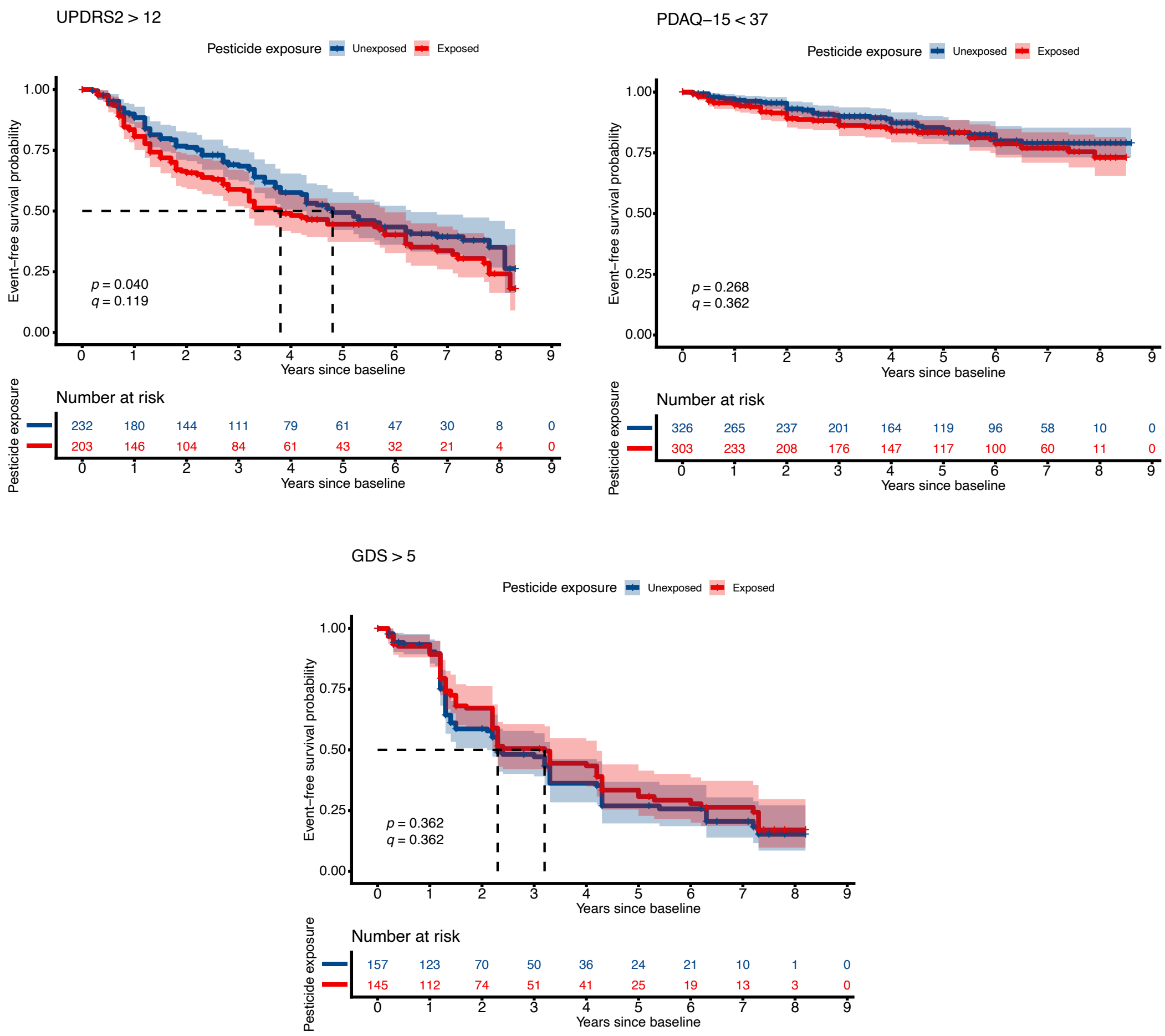

Supplementary Figure 3. Kaplan–Meier event-free survival curves for clinical milestone events by pesticide exposure status in the Fox Insight cohort. Kaplan–Meier estimates of event-free survival probability for three pre-specified milestone events in the propensity score–matched Fox Insight cohort, stratified by pesticide exposure status (blue: unexposed; red: exposed). Shaded areas represent 95% confidence bands. Milestone thresholds were defined as: UPDRS2 > 12, moderate motor impairment; PDAQ-15 < 37, dementia-level cognitive impairment; GDS > 5, clinically significant depression. Participants who had already reached the corresponding threshold at baseline were excluded from each analysis. Dashed horizontal lines indicate 50% event-free survival probability; dashed vertical lines mark the corresponding median event-free survival time where applicable. Number-at-risk tables are shown below each panel. Nominal p-values are from log-rank tests; q-values reflect Benjamini–Hochberg false discovery rate correction across the three milestone events in this cohort. The motor milestone (UPDRS2 > 12) showed a consistent trend toward earlier event occurrence in the exposed group (p = 0.040; q = 0.119), whereas the cognitive (PDAQ-15 < 37; p = 0.268) and depression (GDS > 5; p = 0.362) milestones did not reach statistical significance. UPDRS II, Movement Disorder Society–Unified Parkinson's Disease Rating Scale Part II; PDAQ-15, Penn Parkinson's Daily Activities Questionnaire-15; GDS, Geriatric Depression Scale.

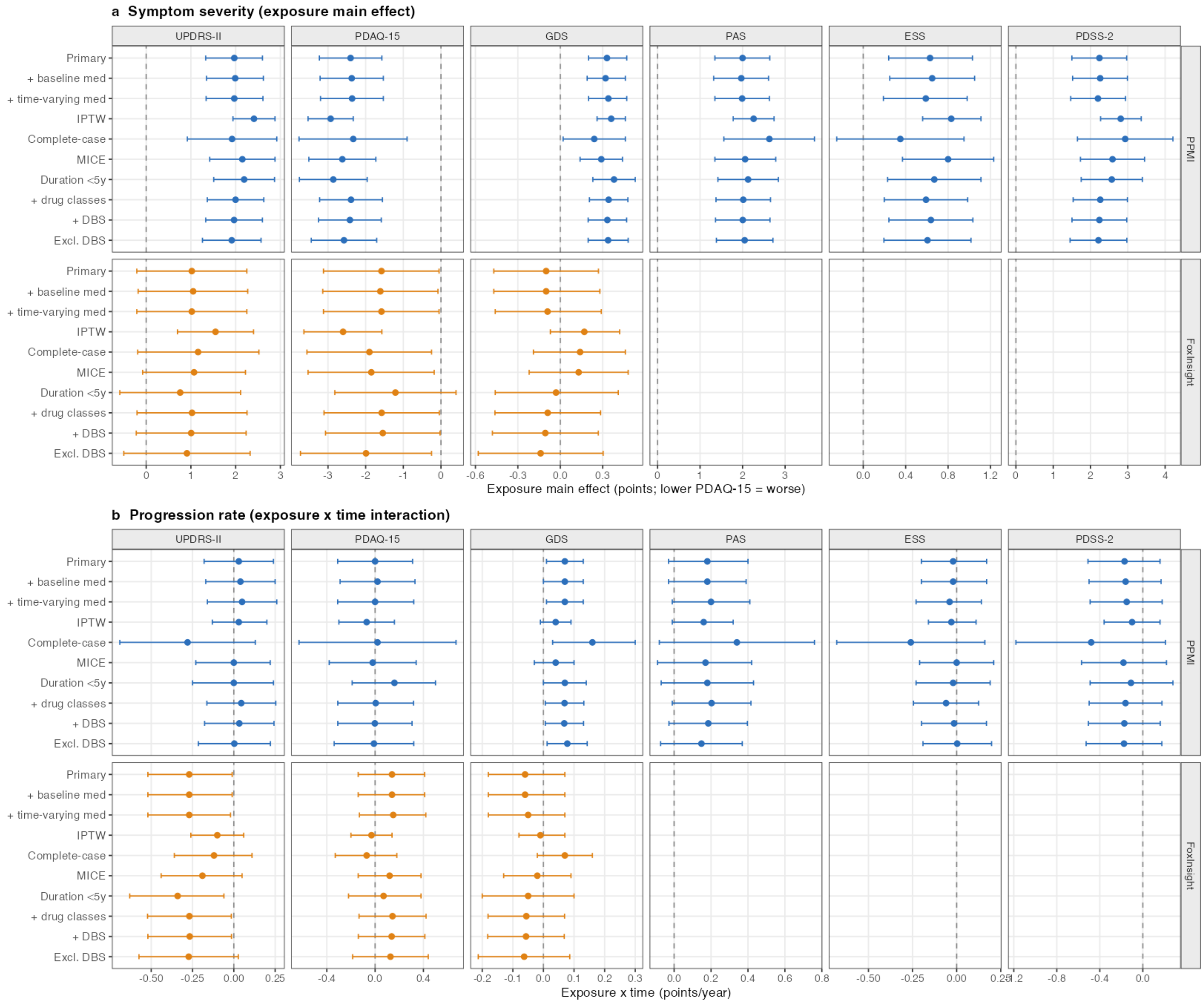

Supplementary Figure 4. Robustness of the exposure–severity and exposure–progression associations across all sensitivity analyses. Two-panel forest plot summarising, for the continuous outcomes, both components of the linear mixed-effects models under every sensitivity analysis, conducted to display at a glance the robustness of the severity finding and the consistency of the (near-null) progression finding. (a) The exposure main effect (the association of pesticide exposure with symptom severity) and (b) the exposure × time interaction (the association with progression rate), each as the coefficient (raw scale points; for PDAQ-15 lower scores indicate worse function) with its 95% CI from the corresponding linear mixed-effects model (primary LMM specification, with the additional covariate, weighting scheme, or restriction indicated by the row). Rows are the primary analysis; baseline- and time-varying-medication adjustment; full-cohort IPTW; complete-case analysis; multiple imputation; the less-than-five-year disease-duration subset; and adjustment for drug classes, adjustment for DBS, and exclusion of DBS participants. Panels are arranged by outcome scale (columns) and cohort (rows within each panel: PPMI Online in blue, Fox Insight in orange); the dashed vertical line marks the null. Across analyses the severity estimates (panel a) are stable and remain on the same side of the null in PPMI Online and directionally consistent in Fox Insight, whereas the interaction estimates (panel b) are small and centred near zero, with only two isolated nominal exceptions (a positive PPMI Online GDS slope and a negative Fox Insight UPDRS-II slope), visually confirming robustness of the severity association and the absence of a consistent progression difference.

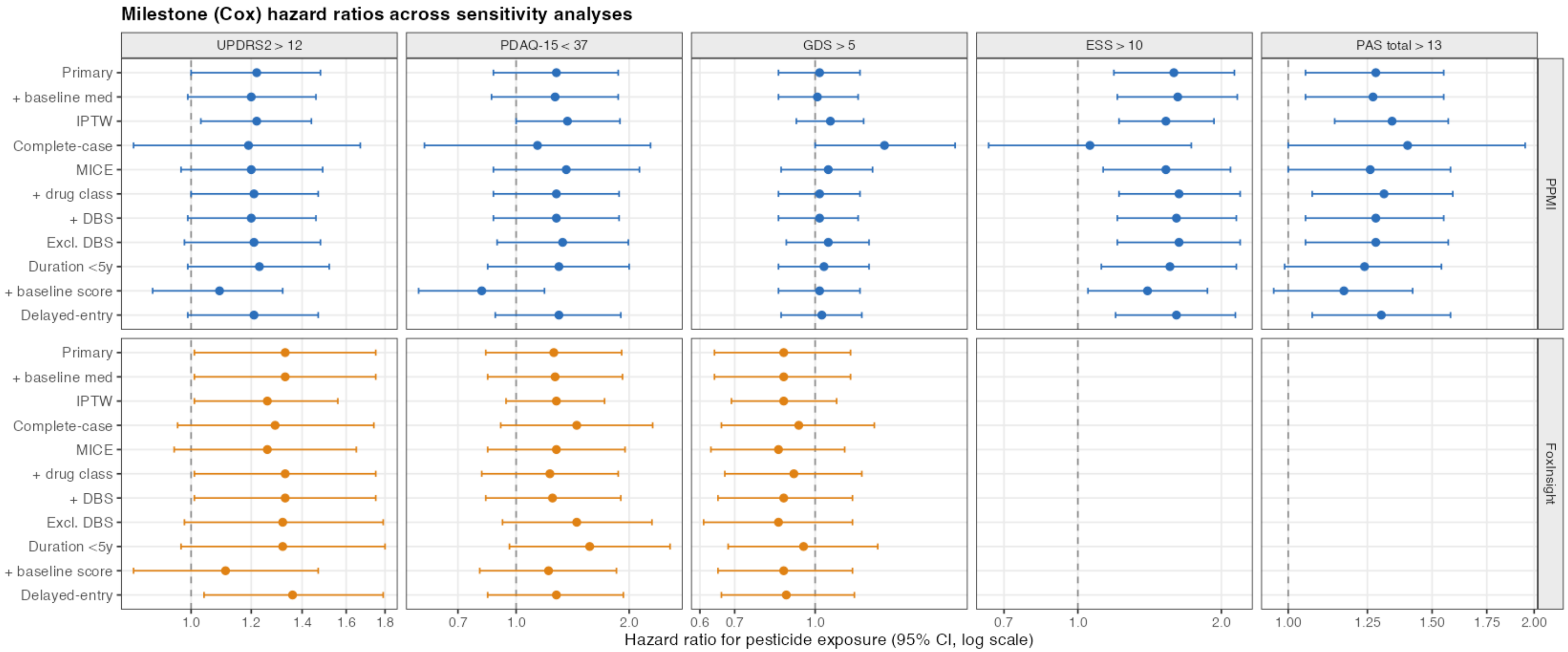

Supplementary Figure 5. Milestone (Cox) hazard ratios for pesticide exposure across all sensitivity analyses. Forest plot summarising the exposure hazard ratio for each clinically meaningful milestone event under every sensitivity analysis, conducted to display the robustness of the milestone (time-to-event) results in parallel with the continuous-outcome figure (Supplementary Figure 4). Each point shows the exposure hazard ratio with its 95% CI (log scale) from the corresponding Cox proportional hazards model (primary milestone specification, with the additional covariate, weighting scheme, or restriction indicated by the row). Rows are the primary analysis; baseline-medication, full-cohort IPTW, complete-case, multiple-imputation, drug-class, DBS-adjustment, DBS-exclusion, and less-than-five-year-duration analyses; and the baseline-score-adjusted and delayed-entry models (Supplementary Table 17). Panels are arranged by milestone endpoint (columns) and cohort (rows: PPMI Online in blue, Fox Insight in orange); the dashed vertical line marks the null (hazard ratio = 1). The hazard ratios are stable across analyses; the wider complete-case intervals reflect the smaller complete-case sample, and the attenuation under baseline-score adjustment for the motor and anxiety milestones is consistent with a contribution of greater baseline symptom severity rather than clearly faster progression. The cognitive (PDAQ-15) and depression (GDS) milestones were not consistently elevated.

Supplementary Table 1. Covariate Balance Between Exposed and Unexposed Groups Within Each Scale-Specific Analysis Subset After Propensity Score Matching.

Cohort	Scale	Covariate	Level	-pesticide	+pesticide	*p* value	SMD
PPMI	UPDRS II	Baseline age	mean (SD)	67.26 (8.70)	67.19 (8.93)	0.849	0.008
		Sex	female	226 (21.4)	226 (21.4)	1.000	0.001
			male	828 (78.6)	831 (78.6)		
		Disease duration	mean (SD)	2.81 (2.43)	2.86 (2.46)	0.688	0.018
		Education years	mean (SD)	16.00 (3.14)	15.83 (3.15)	0.194	0.057
		Family history	no	644 (61.1)	645 (61.0)	0.592	0.045
			yes	261 (24.8)	248 (23.5)		
			unknown	149 (14.1)	164 (15.5)		
		Smoking	no	467 (44.3)	491 (46.5)	0.552	0.047
			yes	324 (30.7)	320 (30.3)		
			unknown	263 (25.0)	246 (23.3)		
		Coffee	no	141 (13.4)	133 (12.6)	0.837	0.026
			yes	568 (53.9)	580 (54.9)		
			unknown	345 (32.7)	344 (32.5)		
		Race	asian	11 (1.0)	7 (0.7)	0.598	0.083
			black	5 (0.5)	3 (0.3)		
			multiracial	19 (1.8)	20 (1.9)		
			other	6 (0.6)	2 (0.2)		
			unknown	19 (1.8)	22 (2.1)		
			white	994 (94.3)	1003 (94.9)		
		Ethnicity	ashkjew	52 (4.9)	55 (5.2)	0.957	0.024
			hispanic	18 (1.7)	16 (1.5)		
			other	940 (89.2)	945 (89.4)		
			unknown	44 (4.2)	41 (3.9)		
	PDSS-2	Baseline age	mean (SD)	67.27 (8.70)	67.16 (8.93)	0.777	0.012
		Sex	female	225 (21.4)	225 (21.4)	1.000	0.002
			male	824 (78.6)	828 (78.6)		
		Disease duration	mean (SD)	2.81 (2.43)	2.85 (2.46)	0.655	0.019
		Education years	mean (SD)	16.02 (3.12)	15.83 (3.15)	0.174	0.059
		Family history	no	642 (61.2)	644 (61.2)	0.544	0.048
			yes	262 (25.0)	248 (23.6)		
			unknown	145 (13.8)	161 (15.3)		
		Smoking	no	467 (44.5)	488 (46.3)	0.651	0.040
			yes	323 (30.8)	320 (30.4)		
			unknown	259 (24.7)	245 (23.3)		

	Coffee	no	141 (13.4)	133 (12.6)	0.839	0.026
		yes	567 (54.1)	579 (55.0)		
		unknown	341 (32.5)	341 (32.4)		
	Race	asian	11 (1.0)	7 (0.7)	0.707	0.075
		black	5 (0.5)	3 (0.3)		
		multiracial	19 (1.8)	20 (1.9)		
		other	5 (0.5)	2 (0.2)		
		unknown	19 (1.8)	22 (2.1)		
		white	990 (94.4)	999 (94.9)		
	Ethnicity	ashkjew	52 (5.0)	54 (5.1)	0.980	0.019
		hispanic	17 (1.6)	16 (1.5)		
		other	936 (89.2)	942 (89.5)		
		unknown	44 (4.2)	41 (3.9)		
PDAQ-15	Baseline age	mean (SD)	67.23 (8.72)	67.21 (8.93)	0.943	0.003
	Sex	female	225 (21.4)	226 (21.3)	1.000	0.001
		male	828 (78.6)	833 (78.7)		
	Disease duration	mean (SD)	2.81 (2.43)	2.86 (2.46)	0.655	0.019
	Education years	mean (SD)	16.00 (3.14)	15.83 (3.15)	0.202	0.056
	Family history	no	642 (61.0)	647 (61.1)	0.619	0.043
		yes	261 (24.8)	248 (23.4)		
		unknown	150 (14.2)	164 (15.5)		
	Smoking	no	466 (44.3)	491 (46.4)	0.547	0.048
		yes	323 (30.7)	321 (30.3)		
		unknown	264 (25.1)	247 (23.3)		
	Coffee	no	141 (13.4)	133 (12.6)	0.846	0.025
		yes	569 (54.0)	580 (54.8)		
		unknown	343 (32.6)	346 (32.7)		
	Race	asian	11 (1.0)	7 (0.7)	0.541	0.088
		black	5 (0.5)	3 (0.3)		
		multiracial	19 (1.8)	20 (1.9)		
		other	6 (0.6)	2 (0.2)		
		unknown	18 (1.7)	23 (2.2)		
		white	994 (94.4)	1004 (94.8)		
	Ethnicity	ashkjew	52 (4.9)	55 (5.2)	0.975	0.020
		hispanic	18 (1.7)	16 (1.5)		
		other	940 (89.3)	946 (89.3)		
		unknown	43 (4.1)	42 (4.0)		
GDS	Baseline age	mean (SD)	67.23 (8.69)	67.12 (8.91)	0.772	0.013
	Sex	female	221 (21.2)	224 (21.4)	0.996	0.003
		male	819 (78.8)	825 (78.6)		
	Disease duration	mean (SD)	2.81 (2.43)	2.86 (2.47)	0.646	0.020
	Education years	mean (SD)	16.02 (3.11)	15.83 (3.16)	0.164	0.061

	Family history	no	638 (61.3)	642 (61.2)	0.590	0.045
		yes	258 (24.8)	247 (23.5)		
		unknown	144 (13.8)	160 (15.3)		
	Smoking	no	464 (44.6)	487 (46.4)	0.596	0.045
		yes	318 (30.6)	320 (30.5)		
		unknown	258 (24.8)	242 (23.1)		
	Coffee	no	140 (13.5)	133 (12.7)	0.865	0.024
		yes	565 (54.3)	577 (55.0)		
		unknown	335 (32.2)	339 (32.3)		
	Race	asian	11 (1.1)	7 (0.7)	0.691	0.077
		black	5 (0.5)	3 (0.3)		
		multiracial	18 (1.7)	20 (1.9)		
		other	5 (0.5)	2 (0.2)		
		unknown	18 (1.7)	21 (2.0)		
		white	983 (94.5)	996 (94.9)		
	Ethnicity	ashkjew	52 (5.0)	54 (5.1)	0.984	0.018
		hispanic	16 (1.5)	16 (1.5)		
		other	929 (89.3)	939 (89.5)		
		unknown	43 (4.1)	40 (3.8)		
PAS	Baseline age	mean (SD)	67.25 (8.71)	67.14 (8.92)	0.766	0.013
	Sex	female	223 (21.3)	224 (21.3)	1.000	<0.001
		male	824 (78.7)	827 (78.7)		
	Disease duration	mean (SD)	2.80 (2.43)	2.86 (2.47)	0.603	0.023
	Education years	mean (SD)	16.00 (3.15)	15.83 (3.16)	0.194	0.057
	Family history	no	640 (61.1)	643 (61.2)	0.572	0.046
		yes	261 (24.9)	247 (23.5)		
		unknown	146 (13.9)	161 (15.3)		
	Smoking	no	465 (44.4)	488 (46.4)	0.551	0.048
		yes	321 (30.7)	320 (30.4)		
		unknown	261 (24.9)	243 (23.1)		
	Coffee	no	141 (13.5)	133 (12.7)	0.839	0.026
		yes	568 (54.3)	580 (55.2)		
		unknown	338 (32.3)	338 (32.2)		
	Race	asian	11 (1.1)	7 (0.7)	0.595	0.084
		black	5 (0.5)	3 (0.3)		
		multiracial	19 (1.8)	20 (1.9)		
		other	6 (0.6)	2 (0.2)		
		unknown	18 (1.7)	21 (2.0)		
		white	988 (94.4)	998 (95.0)		
	Ethnicity	ashkjew	52 (5.0)	54 (5.1)	0.979	0.019
		hispanic	17 (1.6)	16 (1.5)		
		other	935 (89.3)	941 (89.5)		
		unknown	43 (4.1)	40 (3.8)		

	ESS	Baseline age	mean (SD)	67.27 (8.49)	67.35 (8.74)	0.856	0.009
		Sex	female	166 (20.7)	179 (22.0)	0.567	0.032
			male	636 (79.3)	635 (78.0)		
		Disease duration	mean (SD)	2.82 (2.42)	2.79 (2.41)	0.788	0.013
		Education years	mean (SD)	16.27 (2.94)	15.96 (3.11)	0.040	0.102
		Family history	no	496 (61.8)	502 (61.7)	0.618	0.049
			yes	198 (24.7)	190 (23.3)		
			unknown	108 (13.5)	122 (15.0)		
		Smoking	no	468 (58.4)	491 (60.3)	0.035	0.129
			yes	323 (40.3)	321 (39.4)		
			unknown	11 (1.4)	2 (0.2)		
		Coffee	no	95 (11.8)	102 (12.5)	0.909	0.022
			yes	398 (49.6)	403 (49.5)		
			unknown	309 (38.5)	309 (38.0)		
		Race	asian	5 (0.6)	2 (0.2)	0.858	0.069
			black	3 (0.4)	2 (0.2)		
			multiracial	13 (1.6)	13 (1.6)		
			other	1 (0.1)	2 (0.2)		
			unknown	14 (1.7)	13 (1.6)		
			white	766 (95.5)	782 (96.1)		
		Ethnicity	ashkjew	37 (4.6)	42 (5.2)	0.961	0.027
			hispanic	12 (1.5)	13 (1.6)		
			other	717 (89.4)	723 (88.8)		
			unknown	36 (4.5)	36 (4.4)		
FoxInsight	UPDRS II	Baseline age	mean (SD)	64.30 (9.38)	64.40 (9.72)	0.897	0.010
		Sex	male	229 (67.6)	239 (69.7)	0.606	0.046
			female	110 (32.4)	104 (30.3)		
		Disease duration	mean (SD)	3.36 (2.56)	3.21 (2.60)	0.435	0.060
		Education level	high	207 (61.1)	214 (62.4)	0.988	0.027
			low	2 (0.6)	2 (0.6)		
			medium	129 (38.1)	126 (36.7)		
			unknown	1 (0.3)	1 (0.3)		
		Family history	no	194 (57.2)	204 (59.5)	0.713	0.063
			yes	102 (30.1)	102 (29.7)		
			unknown	43 (12.7)	37 (10.8)		
		Smoking	no	213 (62.8)	225 (65.6)	0.428	0.100
			yes	121 (35.7)	116 (33.8)		
			unknown	5 (1.5)	2 (0.6)		
		Coffee	yes	264 (77.9)	264 (77.0)	0.848	0.022
			no	75 (22.1)	79 (23.0)		
		Race	asian	3 (0.9)	5 (1.5)	0.869	0.105
			black	1 (0.3)	2 (0.6)		

		multiracial	5 (1.5)	3 (0.9)		
		other	2 (0.6)	1 (0.3)		
		unknown	2 (0.6)	3 (0.9)		
		white	326 (96.2)	329 (95.9)		
	Ethnicity	hispanic	19 (5.6)	14 (4.1)	0.454	0.071
		other	320 (94.4)	329 (95.9)		
PDAQ-15	Baseline age	mean (SD)	64.31 (9.41)	64.29 (9.77)	0.983	0.002
	Sex	male	227 (67.4)	241 (69.7)	0.573	0.049
		female	110 (32.6)	105 (30.3)		
	Disease duration	mean (SD)	3.35 (2.55)	3.21 (2.60)	0.468	0.056
	Education level	high	205 (60.8)	215 (62.1)	0.989	0.027
		low	2 (0.6)	2 (0.6)		
		medium	129 (38.3)	128 (37.0)		
		unknown	1 (0.3)	1 (0.3)		
	Family history	no	194 (57.6)	206 (59.5)	0.692	0.066
		yes	100 (29.7)	103 (29.8)		
		unknown	43 (12.8)	37 (10.7)		
	Smoking	no	211 (62.6)	228 (65.9)	0.381	0.106
		yes	121 (35.9)	116 (33.5)		
		unknown	5 (1.5)	2 (0.6)		
	Coffee	yes	262 (77.7)	267 (77.2)	0.929	0.014
		no	75 (22.3)	79 (22.8)		
	Race	asian	3 (0.9)	5 (1.4)	0.870	0.104
		black	1 (0.3)	2 (0.6)		
		multiracial	5 (1.5)	3 (0.9)		
		other	2 (0.6)	1 (0.3)		
		unknown	2 (0.6)	3 (0.9)		
		white	324 (96.1)	332 (96.0)		
	Ethnicity	hispanic	18 (5.3)	14 (4.0)	0.536	0.061
		other	319 (94.7)	332 (96.0)		
GDS	Baseline age	mean (SD)	64.31 (9.42)	64.45 (9.70)	0.847	0.015
	Sex	male	226 (67.3)	237 (69.5)	0.586	0.048
		female	110 (32.7)	104 (30.5)		
	Disease duration	mean (SD)	3.35 (2.56)	3.22 (2.60)	0.526	0.049
	Education level	high	204 (60.7)	213 (62.5)	0.974	0.036
		low	2 (0.6)	2 (0.6)		
		medium	129 (38.4)	125 (36.7)		
		unknown	1 (0.3)	1 (0.3)		
	Family history	no	193 (57.4)	202 (59.2)	0.727	0.061
		yes	100 (29.8)	102 (29.9)		
		unknown	43 (12.8)	37 (10.9)		
	Smoking	no	211 (62.8)	225 (66.0)	0.396	0.105

	yes	120 (35.7)	114 (33.4)		
	unknown	5 (1.5)	2 (0.6)		
Coffee	yes	261 (77.7)	262 (76.8)	0.864	0.020
	no	75 (22.3)	79 (23.2)		
Race	asian	3 (0.9)	5 (1.5)	0.869	0.105
	black	1 (0.3)	2 (0.6)		
	multiracial	5 (1.5)	3 (0.9)		
	other	2 (0.6)	1 (0.3)		
	unknown	2 (0.6)	3 (0.9)		
	white	323 (96.1)	327 (95.9)		
Ethnicity	hispanic	18 (5.4)	14 (4.1)	0.558	0.059
	other	318 (94.6)	327 (95.9)		

Baseline characteristics of the pesticide-exposed (+pesticide) and unexposed (−pesticide) groups within the analysis subset corresponding to each outcome scale, assessed after propensity score matching. For each scale, the analysis subset was defined as the intersection of the matched cohort and participants with a valid baseline total score for that scale. Covariate balance was re-evaluated within each subset to confirm that the balance achieved in the overall matched cohort was maintained. Continuous variables are shown as mean (SD); categorical variables are shown as n (%). p-values are from two-sample t-tests (continuous variables) and χ^2 tests (categorical variables). SMD, absolute standardized mean difference; values < 0.10 indicate adequate covariate balance. Covariates included baseline age, sex, disease duration at baseline, education years, family history of PD, smoking history, coffee consumption history, race, and ethnicity. For subsets that did not meet the balance criterion (SMD ≥ 0.10 for any covariate), propensity score matching was repeated and sensitivity analyses were conducted in the re-matched cohort (see Methods). UPDRS II, Movement Disorder Society–Unified Parkinson's Disease Rating Scale Part II; GDS, Geriatric Depression Scale; PDAQ-15, Penn Parkinson's Daily Activities Questionnaire-15; ESS, Epworth Sleepiness Scale; PDSS-2, Parkinson's Disease Sleep Scale-2; PAS, Parkinson Anxiety Scale.

Supplementary Table 2. Linear Mixed-Effects Model Estimates for Overall Symptom Progression Across Scales in the PPMI Online and Fox Insight Cohorts.

Cohort	Scale	Term	Estimate	SE	*p* value
**PPMI**	UPDRS II	(Intercept)	5.918	1.218	1.26e-06
		Baseline age	0.051	0.017	0.003
		Sex (male)	0.612	0.373	0.101
		Disease duration	0.791	0.063	3.80e-35
		**Time**	**1.157**	**0.053**	**5.46e-88**
	GDS	(Intercept)	7.292	0.272	6.05e-136
		Baseline age	-0.018	0.004	2.40e-06
		Sex (male)	-0.119	0.083	0.152
		Disease duration	0.031	0.014	0.029
		**Time**	**0.039**	**0.016**	**0.014**
	PDAQ-15	(Intercept)	54.938	1.659	6.95e-194
		Baseline age	-0.062	0.024	0.009
		Sex (male)	-0.100	0.510	0.845
		Disease duration	-0.429	0.086	5.95e-07
		**Time**	**-0.973**	**0.078**	**4.25e-33**
	ESS	(Intercept)	7.006	0.814	1.76e-17
		Baseline age	-0.011	0.012	0.368
		Sex (male)	0.925	0.246	1.74e-04
		Disease duration	0.205	0.042	1.04e-06
		**Time**	**0.250**	**0.047**	**1.40e-07**
	PDSS-2	(Intercept)	25.575	1.501	5.54e-61
		Baseline age	-0.123	0.022	1.33e-08
		Sex (male)	-1.117	0.461	0.016
		Disease duration	0.451	0.077	6.65e-09
		**Time**	**0.235**	**0.085**	**0.006**
	PAS	(Intercept)	22.230	1.299	1.78e-61
		Baseline age	-0.142	0.019	4.37e-14
		Sex (male)	-2.892	0.399	6.34e-13
		Disease duration	0.128	0.067	0.055
		**Time**	**0.067**	**0.054**	**0.214**
**FoxInsight**	UPDRS II	(Intercept)	10.550	1.864	2.21e-08
		Baseline age	-0.003	0.028	0.917
		Sex (male)	1.274	0.576	0.027
		Disease duration	0.774	0.103	2.37e-13
		**Time**	**1.029**	**0.065**	**1.95e-43**
	PDAQ-15	(Intercept)	50.271	2.330	2.63e-79
		Baseline age	0.010	0.035	0.784
		Sex (male)	-1.018	0.724	0.160
		Disease duration	-0.460	0.131	4.57e-04
		**Time**	**-1.035**	**0.070**	**2.07e-38**

GDS	(Intercept)	7.973	0.502	9.10e-49
	Baseline age	-0.023	0.008	0.003
	Sex (male)	0.128	0.153	0.402
	Disease duration	0.005	0.028	0.845
	**Time**	**0.231**	**0.032**	**1.77e-12**

Fixed-effect coefficients from linear mixed-effects models fitted to each outcome scale within the propensity score–matched cohorts. Each model specified the scale total score as the dependent variable, with time (years since baseline assessment), baseline age, sex (male), and disease duration at baseline as fixed effects. Patient ID was included as a random effect with both random intercepts and random slopes for time. Models were estimated using restricted maximum likelihood (REML). Time coefficients (bolded) represent the estimated annual rate of change in scale scores across the full matched cohort, without stratification by exposure status. SE, standard error. UPDRS II, Movement Disorder Society–Unified Parkinson's Disease Rating Scale Part II; GDS, Geriatric Depression Scale; PDAQ-15, Penn Parkinson's Daily Activities Questionnaire-15; ESS, Epworth Sleepiness Scale; PDSS-2, Parkinson's Disease Sleep Scale-2; PAS, Parkinson Anxiety Scale. ESS, PAS, and PDSS-2 were assessed in PPMI Online only; Fox Insight included UPDRS II, PDAQ-15, and GDS.

Supplementary Table 3. Linear Mixed-Effects Model Estimates for the Association of Pesticide Exposure With Symptom Severity and Progression Rate Across Scales in the PPMI Online and Fox Insight Cohorts.

Cohort	Scale	Term	Estimate	SE	*p* value
**PPMI**	UPDRS II	Baseline age	0.051	0.017	0.003
		Sex (male)	0.612	0.369	0.098
		Disease duration	0.795	0.062	4.47e-36
		**Time**	**1.140**	**0.075**	**3.26e-47**
		**Exposure**	**1.968**	**0.323**	**1.35e-09**
		**Time:Exposure**	**0.033**	**0.107**	**0.757**
	GDS	Baseline age	-0.019	0.004	1.86e-06
		Sex (male)	-0.125	0.083	0.131
		Disease duration	0.031	0.014	0.025
		**Time**	**0.005**	**0.022**	**0.812**
		**Exposure**	**0.331**	**0.069**	**1.90e-06**
		**Time:Exposure**	**0.068**	**0.032**	**0.032**
	PDAQ-15	Baseline age	-0.062	0.024	0.009
		Sex (male)	-0.098	0.506	0.846
		Disease duration	-0.434	0.085	3.58e-07
		**Time**	**-0.971**	**0.109**	**2.62e-18**
		**Exposure**	**-2.400**	**0.425**	**1.86e-08**
		**Time:Exposure**	**-0.002**	**0.157**	**0.991**
	ESS	Baseline age	-0.010	0.012	0.377
		Sex (male)	0.910	0.245	2.12e-04
		Disease duration	0.204	0.042	1.08e-06
		**Time**	**0.257**	**0.065**	**8.25e-05**
		**Exposure**	**0.634**	**0.202**	**0.002**
		**Time:Exposure**	**-0.015**	**0.094**	**0.872**
	PDSS-2	Baseline age	-0.124	0.021	7.67e-09
		Sex (male)	-1.129	0.457	0.014
		Disease duration	0.456	0.077	3.26e-09
		**Time**	**0.320**	**0.118**	**0.007**
		**Exposure**	**2.236**	**0.374**	**2.79e-09**
		**Time:Exposure**	**-0.172**	**0.170**	**0.312**
	PAS	Baseline age	-0.142	0.018	2.38e-14
		Sex (male)	-2.906	0.396	3.14e-13
		Disease duration	0.134	0.066	0.043
		**Time**	**-0.026**	**0.076**	**0.731**
		**Exposure**	**1.995**	**0.327**	**1.20e-09**
		**Time:Exposure**	**0.184**	**0.108**	**0.089**
**FoxInsight**	UPDRS II	Baseline age	-0.005	0.028	0.865
		Sex (male)	1.303	0.571	0.023
		Disease duration	0.767	0.103	2.59e-13
		**Time**	**1.164**	**0.091**	**1.87e-31**

	**Exposure**	**1.023**	**0.626**	**0.102**
	**Time:Exposure**	**-0.267**	**0.129**	**0.039**
PDAQ-15	Baseline age	0.011	0.035	0.763
	Sex (male)	-1.061	0.719	0.141
	Disease duration	-0.450	0.130	5.64e-04
	**Time**	**-1.103**	**0.098**	**3.12e-25**
	**Exposure**	**-1.585**	**0.780**	**0.043**
	**Time:Exposure**	**0.135**	**0.140**	**0.335**
GDS	Baseline age	-0.023	0.008	0.003
	Sex (male)	0.129	0.153	0.401
	Disease duration	0.005	0.028	0.851
	**Time**	**0.260**	**0.045**	**1.47e-08**
	**Exposure**	**-0.100**	**0.191**	**0.601**
	**Time:Exposure**	**-0.057**	**0.064**	**0.366**

Fixed-effect coefficients from linear mixed-effects models fitted to each outcome scale within the propensity score–matched cohorts. Each model specified the scale total score as the dependent variable, with baseline age, sex (male), disease duration at baseline, time (years since baseline assessment), exposure (occupational pesticide exposure status), and the time × exposure interaction as fixed effects. Patient ID was included as a random effect with both random intercepts and random slopes for time. Models were estimated using restricted maximum likelihood (REML). The exposure coefficient (bolded) represents the estimated between-group difference in mean scale score (i.e., the main effect of exposure on symptom severity); the time × exposure interaction coefficient (bolded, underlined) represents the estimated between-group difference in annual rate of change (i.e., the differential progression rate associated with exposure). Intercept terms were omitted for clarity. SE, standard error. UPDRS II, Movement Disorder Society–Unified Parkinson's Disease Rating Scale Part II; GDS, Geriatric Depression Scale; PDAQ-15, Penn Parkinson's Daily Activities Questionnaire-15; ESS, Epworth Sleepiness Scale; PDSS-2, Parkinson's Disease Sleep Scale-2; PAS, Parkinson Anxiety Scale. ESS, PAS, and PDSS-2 were assessed in PPMI Online only; Fox Insight included UPDRS II, PDAQ-15, and GDS.

Supplementary Table 4. Cox proportional hazards model results for pesticide exposure and PD-related endpoints (PPMI and FoxInsight).

Cohort	Endpoint	Covariate	HR (95% CI)	*p* value
**FoxInsight**	UPDRS2 > 12	Baseline age	1.023 (1.006, 1.040)	0.00835
		Disease duration	1.074 (1.018, 1.133)	0.00927
		Sex (male)	0.756 (0.560, 1.021)	0.0685
		**Exposure**	**1.332 (1.014, 1.750)**	**0.0396**
	PDAQ-15 < 37	Baseline age	1.018 (0.994, 1.042)	0.134
		Disease duration	1.020 (0.941, 1.107)	0.625
		Sex (male)	0.489 (0.288, 0.830)	0.00809
		**Exposure**	**1.264 (0.835, 1.915)**	**0.268**
	GDS > 5	Baseline age	1.002 (0.984, 1.020)	0.841
		Disease duration	0.925 (0.866, 0.987)	0.0195
		Sex (male)	0.844 (0.613, 1.161)	0.298
		**Exposure**	**0.870 (0.644, 1.174)**	**0.362**
**PPMI**	UPDRS2 > 12	Baseline age	1.018 (1.006, 1.031)	0.00352
		Disease duration	1.079 (1.035, 1.125)	0.000331
		Sex (male)	1.329 (1.033, 1.709)	0.0267
		**Exposure**	**1.220 (1.005, 1.482)**	**0.0449**
	PDAQ-15 < 37	Baseline age	1.018 (0.994, 1.043)	0.137
		Disease duration	1.035 (0.959, 1.118)	0.378
		Sex (male)	1.450 (0.850, 2.473)	0.173
		**Exposure**	**1.275 (0.869, 1.870)**	**0.214**
	GDS > 5	Baseline age	1.005 (0.993, 1.017)	0.403
		Disease duration	1.024 (0.986, 1.064)	0.214
		Sex (male)	1.012 (0.808, 1.269)	0.915
		**Exposure**	**1.017 (0.850, 1.215)**	**0.857**
	ESS > 10	Baseline age	1.010 (0.992, 1.028)	0.29
		Disease duration	1.041 (0.982, 1.104)	0.177
		Sex (male)	1.272 (0.876, 1.846)	0.206
		**Exposure**	**1.594 (1.195, 2.127)**	**0.00154**
	PAS total > 13	Baseline age	0.986 (0.974, 0.998)	0.0215
		Disease duration	1.015 (0.975, 1.057)	0.456
		Sex (male)	0.588 (0.468, 0.739)	0.00000514
		**Exposure**	**1.279 (1.052, 1.555)**	**0.0137**

Hazard ratios from multivariable Cox proportional hazards models evaluating the association between occupational pesticide exposure and the risk of reaching pre-specified clinically meaningful milestone events during follow-up. Models were fitted within the propensity score–matched cohorts, restricted to participants who had not reached the corresponding threshold at baseline. Time zero was defined as the date of the baseline assessment; event time was defined as the time to the first visit at which the threshold was reached. Participants who did not experience the event were censored at their last valid assessment. Each model included pesticide exposure status (bolded) as the primary predictor, with baseline age, disease duration at baseline, and sex (male) as covariates. Milestone thresholds were defined as: UPDRS2 > 12, moderate motor impairment; PDAQ-15 < 37, dementia-level cognitive impairment; GDS >

5, clinically significant depression; ESS > 10, excessive daytime sleepiness; PAS total > 13, anxiety disorder. ESS and PAS were assessed in PPMI Online only; Fox Insight included UPDRS II, PDAQ-15, and GDS. HR, hazard ratio; CI, confidence interval. UPDRS II, Movement Disorder Society–Unified Parkinson's Disease Rating Scale Part II; GDS, Geriatric Depression Scale; PDAQ-15, Penn Parkinson's Daily Activities Questionnaire-15; ESS, Epworth Sleepiness Scale; PAS, Parkinson Anxiety Scale.

Supplementary Table 5. Linear Mixed-Effects Model Estimates for the Dose–Response Association Between Number of Co-Exposed Pesticide Functional Classes and Symptom Severity and Progression Rate in the PPMI Online and Fox Insight Cohorts.

Cohort	Scale	Term	Estimate	SE	p value
**PPMI**	UPDRS II	Baseline age	0.054	0.027	0.047
		Sex (male)	0.135	0.604	0.823
		Disease duration	0.666	0.097	9.93e-12
		**Time**	**1.201**	**0.117**	**1.10e-22**
		**n_type_categories2**	**0.083**	**0.581**	**0.886**
		**n_type_categories3**	**0.121**	**0.720**	**0.867**
		**n_type_categories4**	**4.716**	**1.179**	**6.88e-05**
		**Time:n_type_categories2**	**-0.284**	**0.185**	**0.125**
		**Time:n_type_categories3**	**0.040**	**0.235**	**0.864**
		**Time:n_type_categories4**	**-0.158**	**0.403**	**0.694**
	PDSS-2	Baseline age	-0.156	0.033	3.22e-06
		Sex (male)	-2.306	0.748	0.002
		Disease duration	0.441	0.118	2.05e-04
		**Time**	**0.278**	**0.190**	**0.145**
		**n_type_categories2**	**1.377**	**0.679**	**0.043**
		**n_type_categories3**	**2.054**	**0.834**	**0.014**
		**n_type_categories4**	**4.312**	**1.374**	**0.002**
		**Time:n_type_categories2**	**-0.658**	**0.297**	**0.027**
		**Time:n_type_categories3**	**0.365**	**0.368**	**0.322**
		**Time:n_type_categories4**	**0.049**	**0.664**	**0.941**
	PDAQ-15	Baseline age	-0.041	0.038	0.278
		Sex (male)	0.982	0.858	0.253
		Disease duration	-0.307	0.136	0.025
		**Time**	**-1.019**	**0.190**	**1.33e-07**
		**n_type_categories2**	**-0.594**	**0.793**	**0.454**
		**n_type_categories3**	**-1.710**	**0.975**	**0.080**
		**n_type_categories4**	**-3.778**	**1.602**	**0.019**
		**Time:n_type_categories2**	**-0.058**	**0.299**	**0.846**
		**Time:n_type_categories3**	**0.145**	**0.370**	**0.696**
		**Time:n_type_categories4**	**1.160**	**0.659**	**0.079**
	GDS	Baseline age	-0.028	0.006	6.43e-06
		Sex (male)	-0.206	0.137	0.134
		Disease duration	0.026	0.022	0.224
		**Time**	**0.122**	**0.033**	**2.11e-04**
		**n_type_categories2**	**-0.137**	**0.127**	**0.282**
		**n_type_categories3**	**-0.084**	**0.157**	**0.594**
		**n_type_categories4**	**0.564**	**0.258**	**0.029**
		**Time:n_type_categories2**	**-0.113**	**0.051**	**0.027**
		**Time:n_type_categories3**	**0.007**	**0.066**	**0.911**

		**Time:n_type_categories4**	**-0.127**	**0.113**	**0.259**
	PAS	Baseline age	-0.163	0.030	7.15e-08
		Sex (male)	-3.728	0.670	3.45e-08
		Disease duration	0.121	0.107	0.256
		**Time**	**0.396**	**0.124**	**0.001**
		**n_type_categories2**	**0.178**	**0.619**	**0.773**
		**n_type_categories3**	**0.726**	**0.764**	**0.342**
		**n_type_categories4**	**2.644**	**1.248**	**0.034**
		**Time:n_type_categories2**	**-0.577**	**0.196**	**0.003**
		**Time:n_type_categories3**	**-0.122**	**0.249**	**0.625**
		**Time:n_type_categories4**	**-0.388**	**0.425**	**0.361**
	ESS	Baseline age	-0.025	0.017	0.145
		Sex (male)	0.989	0.380	0.009
		Disease duration	0.189	0.060	0.002
		**Time**	**0.186**	**0.106**	**0.080**
		**n_type_categories2**	**0.183**	**0.344**	**0.595**
		**n_type_categories3**	**0.512**	**0.427**	**0.230**
		**n_type_categories4**	**1.247**	**0.730**	**0.088**
		**Time:n_type_categories2**	**0.101**	**0.163**	**0.536**
		**Time:n_type_categories3**	**0.031**	**0.201**	**0.878**
		**Time:n_type_categories4**	**-0.163**	**0.367**	**0.657**
**FoxInsight**	UPDRS II	Baseline age	-0.006	0.046	0.900
		Sex (male)	1.511	0.931	0.106
		Disease duration	0.793	0.161	1.52e-06
		**Time**	**0.873**	**0.152**	**9.98e-08**
		**n_type_categories2**	**0.017**	**1.305**	**0.990**
		**n_type_categories3**	**1.829**	**1.452**	**0.209**
		**n_type_categories4**	**3.847**	**1.562**	**0.015**
		**n_type_categories5**	**4.711**	**1.909**	**0.014**
		**Time:n_type_categories2**	**-0.313**	**0.257**	**0.226**
		**Time:n_type_categories3**	**-0.056**	**0.288**	**0.847**
		**Time:n_type_categories4**	**0.570**	**0.330**	**0.087**
		**Time:n_type_categories5**	**-0.213**	**0.379**	**0.576**
	PDAQ-15	Baseline age	0.075	0.064	0.241
		Sex (male)	-1.224	1.283	0.341
		Disease duration	-0.643	0.224	0.004
		**Time**	**-1.176**	**0.165**	**5.34e-11**
		**n_type_categories2**	**-1.092**	**1.756**	**0.535**
		**n_type_categories3**	**0.846**	**1.944**	**0.664**
		**n_type_categories4**	**-2.343**	**2.071**	**0.259**
		**n_type_categories5**	**-1.826**	**2.553**	**0.475**
		**Time:n_type_categories2**	**0.359**	**0.278**	**0.199**
		**Time:n_type_categories3**	**0.325**	**0.306**	**0.290**
		**Time:n_type_categories4**	**0.018**	**0.347**	**0.959**

	**Time:n_type_categories5**	**1.069**	**0.391**	**0.007**
GDS	Baseline age	-0.022	0.011	0.044
	Sex (male)	0.363	0.218	0.097
	Disease duration	0.018	0.038	0.647
	**Time**	**0.124**	**0.049**	**0.012**
	**n_type_categories2**	**-0.033**	**0.276**	**0.905**
	**n_type_categories3**	**0.234**	**0.305**	**0.444**
	**n_type_categories4**	**-0.013**	**0.342**	**0.969**
	**n_type_categories5**	**1.461**	**0.404**	**3.79e-04**
	**Time:n_type_categories2**	**-0.068**	**0.083**	**0.410**
	**Time:n_type_categories3**	**0.157**	**0.091**	**0.087**
	**Time:n_type_categories4**	**-0.013**	**0.105**	**0.903**
	**Time:n_type_categories5**	**-0.032**	**0.127**	**0.798**

Fixed-effect coefficients from linear mixed-effects models fitted to each outcome scale within the exposed subgroup of the propensity score–matched cohorts. Each model specified the scale total score as the dependent variable, with baseline age, sex (male), disease duration at baseline, time (years since baseline assessment), number of co-exposed pesticide functional classes (categorical), and the time × number-of-classes interaction as fixed effects. Patient ID was included as a random effect with both random intercepts and random slopes for time. Models were estimated using restricted maximum likelihood (REML). The number of pesticide functional classes was coded as a categorical variable with exposure to a single class as the reference category: n_type_categories2, n_type_categories3, and n_type_categories4 denote exposure to 2, 3, and 4 functional classes, respectively (bolded). In the Fox Insight cohort, an additional level (n_type_categories5, exposure to 5 classes) was available owing to the inclusion of rodenticides in the questionnaire. The corresponding time × number-of-classes interaction terms (bolded, underlined) represent the estimated difference in annual rate of change relative to the single-class reference group. Intercept terms were omitted for clarity. SE, standard error. UPDRS II, Movement Disorder Society–Unified Parkinson's Disease Rating Scale Part II; GDS, Geriatric Depression Scale; PDAQ-15, Penn Parkinson's Daily Activities Questionnaire-15; ESS, Epworth Sleepiness Scale; PDSS-2, Parkinson's Disease Sleep Scale-2; PAS, Parkinson Anxiety Scale. ESS, PAS, and PDSS-2 were assessed in PPMI Online only; Fox Insight included UPDRS II, PDAQ-15, and GDS.

Supplementary Table 6. Linear Mixed-Effects Model Estimates for the Association of Cumulative Exposure Duration and Personal Protective Equipment Use With Symptom Severity and Progression Rate in the PPMI Online and Fox Insight Cohorts.

Analysis	Cohort	Scale	Term	Estimate	SE	*p* value
**totexp**	**PPMI**	UPDRS II	Baseline age	0.046	0.027	0.090
			Sex (male)	0.142	0.611	0.816
			Disease duration	0.682	0.098	5.52e-12
			**Time**	**1.192**	**0.106**	**1.88e-26**
			**total_exposure_years**	**0.031**	**0.022**	**0.162**
			**Time:total_exposure_years**	**-0.009**	**0.007**	**0.230**
		PDSS-2	Baseline age	-0.166	0.033	7.81e-07
			Sex (male)	-2.337	0.755	0.002
			Disease duration	0.449	0.119	1.81e-04
			**Time**	**0.196**	**0.171**	**0.251**
			**total_exposure_years**	**0.017**	**0.028**	**0.542**
			**Time:total_exposure_years**	**-0.007**	**0.012**	**0.551**
		PDAQ-15	Baseline age	-0.031	0.038	0.409
			Sex (male)	0.899	0.862	0.297
			Disease duration	-0.334	0.137	0.015
			**Time**	**-1.090**	**0.170**	**3.87e-10**
			**total_exposure_years**	**-0.078**	**0.031**	**0.012**
			**Time:total_exposure_years**	**0.013**	**0.011**	**0.241**
		GDS	Baseline age	-0.028	0.006	4.53e-06
			Sex (male)	-0.198	0.138	0.152
			Disease duration	0.029	0.022	0.181
			**Time**	**0.070**	**0.029**	**0.018**
			**total_exposure_years**	**0.006**	**0.005**	**0.276**
			**Time:total_exposure_years**	**0.001**	**0.002**	**0.585**
		PAS	Baseline age	-0.168	0.030	2.26e-08
			Sex (male)	-3.698	0.672	4.95e-08
			Disease duration	0.134	0.107	0.211
			**Time**	**0.241**	**0.113**	**0.034**
			**total_exposure_years**	**0.038**	**0.024**	**0.123**
			**Time:total_exposure_years**	**-0.007**	**0.008**	**0.378**
		ESS	Baseline age	-0.028	0.017	0.108
			Sex (male)	1.013	0.381	0.008
			Disease duration	0.192	0.060	0.002
			**Time**	**0.214**	**0.093**	**0.022**
			**total_exposure_years**	**0.016**	**0.014**	**0.248**
			**Time:total_exposure_years**	**0.001**	**0.006**	**0.936**
	**FoxInsight**	UPDRS II	Baseline age	-0.017	0.047	0.711
			Sex (male)	1.516	0.952	0.113
			Disease duration	0.800	0.165	2.16e-06

			**Time**	**0.849**	**0.146**	**7.48e-08**
			**total_exposure_years**	**0.028**	**0.041**	**0.500**
			**Time:total_exposure_years**	**-0.001**	**0.010**	**0.930**
		PDAQ-15	Baseline age	0.088	0.064	0.170
			Sex (male)	-1.199	1.297	0.356
			Disease duration	-0.676	0.226	0.003
			**Time**	**-1.128**	**0.156**	**2.50e-11**
			**total_exposure_years**	**-0.070**	**0.056**	**0.213**
			**Time:total_exposure_years**	**0.017**	**0.010**	**0.102**
		GDS	Baseline age	-0.026	0.011	0.020
			Sex (male)	0.349	0.225	0.122
			Disease duration	0.015	0.040	0.713
			**Time**	**0.144**	**0.047**	**0.003**
			**total_exposure_years**	**0.002**	**0.011**	**0.840**
			**Time:total_exposure_years**	**-0.001**	**0.003**	**0.740**
**ppe**	**PPMI**	UPDRS II	Baseline age	0.046	0.027	0.089
			Sex (male)	0.093	0.610	0.879
			Disease duration	0.680	0.098	5.93e-12
			**Time**	**1.195**	**0.096**	**3.22e-31**
			**ppe_everTRUE**	**0.489**	**0.551**	**0.375**
			**Time:ppe_everTRUE**	**-0.286**	**0.178**	**0.108**
		PDSS-2	Baseline age	-0.166	0.033	6.85e-07
			Sex (male)	-2.346	0.751	0.002
			Disease duration	0.458	0.119	1.22e-04
			**Time**	**0.195**	**0.156**	**0.212**
			**ppe_everTRUE**	**1.715**	**0.639**	**0.007**
			**Time:ppe_everTRUE**	**-0.207**	**0.289**	**0.474**
		PDAQ-15	Baseline age	-0.032	0.038	0.401
			Sex (male)	1.027	0.861	0.233
			Disease duration	-0.325	0.137	0.018
			**Time**	**-1.108**	**0.155**	**3.40e-12**
			**ppe_everTRUE**	**-0.979**	**0.746**	**0.190**
			**Time:ppe_everTRUE**	**0.496**	**0.287**	**0.084**
		GDS	Baseline age	-0.028	0.006	4.60e-06
			Sex (male)	-0.211	0.138	0.125
			Disease duration	0.027	0.022	0.208
			**Time**	**0.100**	**0.027**	**2.46e-04**
			**ppe_everTRUE**	**-0.070**	**0.119**	**0.556**
			**Time:ppe_everTRUE**	**-0.067**	**0.050**	**0.180**
		PAS	Baseline age	-0.168	0.030	2.51e-08
			Sex (male)	-3.764	0.672	2.79e-08
			Disease duration	0.127	0.107	0.235
			**Time**	**0.283**	**0.103**	**0.006**
			**ppe_everTRUE**	**-0.013**	**0.581**	**0.982**

		**Time:ppe_everTRUE**	**-0.358**	**0.190**	**0.060**
	ESS	Baseline age	-0.029	0.017	0.100
		Sex (male)	0.974	0.381	0.011
		Disease duration	0.187	0.060	0.002
		**Time**	**0.249**	**0.084**	**0.003**
		**ppe_everTRUE**	**-0.283**	**0.326**	**0.386**
		**Time:ppe_everTRUE**	**-0.107**	**0.158**	**0.499**
**FoxInsight**	UPDRS II	Baseline age	-0.016	0.047	0.727
		Sex (male)	1.459	0.958	0.129
		Disease duration	0.801	0.165	2.27e-06
		**Time**	**0.830**	**0.122**	**7.02e-10**
		**ppe_everTRUE**	**0.306**	**1.068**	**0.774**
		**Time:ppe_everTRUE**	**0.032**	**0.213**	**0.880**
	PDAQ-15	Baseline age	0.084	0.064	0.190
		Sex (male)	-1.305	1.304	0.318
		Disease duration	-0.652	0.227	0.004
		**Time**	**-0.978**	**0.130**	**6.88e-12**
		**ppe_everTRUE**	**1.798**	**1.403**	**0.202**
		**Time:ppe_everTRUE**	**0.112**	**0.230**	**0.628**
	GDS	Baseline age	-0.026	0.011	0.018
		Sex (male)	0.323	0.226	0.154
		Disease duration	0.015	0.039	0.708
		**Time**	**0.096**	**0.039**	**0.015**
		**ppe_everTRUE**	**0.292**	**0.230**	**0.206**
		**Time:ppe_everTRUE**	**0.110**	**0.070**	**0.118**

Fixed-effect coefficients from linear mixed-effects models fitted to each outcome scale within the exposed subgroup of the propensity score–matched cohorts. Each model specified the scale total score as the dependent variable, with baseline age, sex (male), disease duration at baseline, time (years since baseline assessment), the exposure-detail variable, and the time × exposure-detail interaction as fixed effects. Patient ID was included as a random effect with both random intercepts and random slopes for time. Models were estimated using restricted maximum likelihood (REML). Two exposure-detail variables were analyzed separately (indicated in the "Analysis" column): (1) total_exposure_years — cumulative exposure duration in years, defined as the sum of exposure years across all reported age periods, entered as a continuous variable (bolded); (2) ppe — ever use of personal protective equipment during occupational pesticide exposure, entered as a binary variable (bolded). The corresponding time × exposure-detail interaction terms (bolded, underlined) represent the estimated modification of the annual rate of change by each exposure-detail variable. Intercept terms were omitted for clarity. SE, standard error. UPDRS II, Movement Disorder Society–Unified Parkinson's Disease Rating Scale Part II; GDS, Geriatric Depression Scale; PDAQ-15, Penn Parkinson's Daily Activities Questionnaire-15; ESS, Epworth Sleepiness Scale; PDSS-2, Parkinson's Disease Sleep Scale-2; PAS, Parkinson Anxiety Scale. ESS, PAS, and PDSS-2 were assessed in PPMI Online only; Fox Insight included UPDRS II, PDAQ-15, and GDS.

Supplementary Table 7. Linear Mixed-Effects Model Estimates for the Association of Specific Pesticide Functional Classes With Symptom Severity and Progression Rate in the PPMI Online and Fox Insight Cohorts.

Analysis	Cohort	Scale	Term	Estimate	SE	*p* value
**sptype**	**PPMI**	UPDRS II	Baseline age	0.053	0.027	0.053
			Sex (male)	0.060	0.621	0.923
			Disease duration	0.666	0.097	1.56e-11
			**Time**	**1.240**	**0.255**	**1.51e-06**
			**ever_herbicideTRUE**	**0.706**	**0.697**	**0.311**
			**ever_fungicideTRUE**	**0.504**	**0.627**	**0.421**
			**ever_insecticideTRUE**	**0.447**	**0.564**	**0.428**
			**ever_fumigantTRUE**	**1.555**	**0.837**	**0.064**
			**Time:ever_herbicideTRUE**	**-0.125**	**0.230**	**0.587**
			**Time:ever_fungicideTRUE**	**-0.229**	**0.204**	**0.263**
			**Time:ever_insecticideTRUE**	**-0.009**	**0.183**	**0.959**
			**Time:ever_fumigantTRUE**	**0.339**	**0.280**	**0.227**
		PDSS-2	Baseline age	-0.159	0.033	2.17e-06
			Sex (male)	-2.287	0.765	0.003
			Disease duration	0.448	0.119	1.71e-04
			**Time**	**0.496**	**0.408**	**0.225**
			**ever_herbicideTRUE**	**1.473**	**0.814**	**0.071**
			**ever_fungicideTRUE**	**1.699**	**0.731**	**0.020**
			**ever_insecticideTRUE**	**1.137**	**0.655**	**0.083**
			**ever_fumigantTRUE**	**0.423**	**0.969**	**0.662**
			**Time:ever_herbicideTRUE**	**-0.112**	**0.365**	**0.759**
			**Time:ever_fungicideTRUE**	**0.714**	**0.321**	**0.027**
			**Time:ever_insecticideTRUE**	**-0.833**	**0.295**	**0.005**
			**Time:ever_fumigantTRUE**	**0.631**	**0.448**	**0.160**
		PDAQ-15	Baseline age	-0.044	0.038	0.253
			Sex (male)	0.793	0.877	0.366
			Disease duration	-0.318	0.137	0.020
			**Time**	**-0.967**	**0.411**	**0.019**
			**ever_herbicideTRUE**	**-0.247**	**0.948**	**0.794**
			**ever_fungicideTRUE**	**-1.247**	**0.849**	**0.142**
			**ever_insecticideTRUE**	**-1.042**	**0.763**	**0.173**
			**ever_fumigantTRUE**	**-1.094**	**1.130**	**0.333**
			**Time:ever_herbicideTRUE**	**-0.206**	**0.371**	**0.578**
			**Time:ever_fungicideTRUE**	**0.593**	**0.324**	**0.068**
			**Time:ever_insecticideTRUE**	**0.099**	**0.295**	**0.737**
			**Time:ever_fumigantTRUE**	**-0.276**	**0.448**	**0.539**
		GDS	Baseline age	-0.027	0.006	1.20e-05
			Sex (male)	-0.197	0.141	0.162
			Disease duration	0.027	0.022	0.217

		**Time**	**0.141**	**0.072**	**0.050**
		**ever_herbicideTRUE**	**-0.021**	**0.152**	**0.890**
		**ever_fungicideTRUE**	**-0.023**	**0.137**	**0.867**
		**ever_insecticideTRUE**	**0.029**	**0.123**	**0.812**
		**ever_fumigantTRUE**	**0.263**	**0.182**	**0.150**
		**Time:ever_herbicideTRUE**	**-0.015**	**0.064**	**0.817**
		**Time:ever_fungicideTRUE**	**0.016**	**0.057**	**0.785**
		**Time:ever_insecticideTRUE**	**-0.088**	**0.052**	**0.090**
		**Time:ever_fumigantTRUE**	**0.010**	**0.079**	**0.901**
	PAS	Baseline age	-0.162	0.030	7.73e-08
		Sex (male)	-3.673	0.686	1.07e-07
		Disease duration	0.125	0.107	0.242
		**Time**	**0.737**	**0.270**	**0.007**
		**ever_herbicideTRUE**	**0.049**	**0.739**	**0.947**
		**ever_fungicideTRUE**	**0.963**	**0.666**	**0.149**
		**ever_insecticideTRUE**	**0.329**	**0.596**	**0.581**
		**ever_fumigantTRUE**	**0.577**	**0.883**	**0.514**
		**Time:ever_herbicideTRUE**	**-0.481**	**0.244**	**0.049**
		**Time:ever_fungicideTRUE**	**-0.057**	**0.218**	**0.792**
		**Time:ever_insecticideTRUE**	**-0.272**	**0.195**	**0.164**
		**Time:ever_fumigantTRUE**	**0.167**	**0.296**	**0.573**
	ESS	Baseline age	-0.024	0.017	0.164
		Sex (male)	0.980	0.388	0.012
		Disease duration	0.192	0.061	0.002
		**Time**	**0.144**	**0.224**	**0.520**
		**ever_herbicideTRUE**	**0.394**	**0.415**	**0.343**
		**ever_fungicideTRUE**	**0.044**	**0.364**	**0.903**
		**ever_insecticideTRUE**	**0.481**	**0.333**	**0.148**
		**ever_fumigantTRUE**	**0.483**	**0.513**	**0.347**
		**Time:ever_herbicideTRUE**	**0.086**	**0.199**	**0.666**
		**Time:ever_fungicideTRUE**	**-0.068**	**0.174**	**0.695**
		**Time:ever_insecticideTRUE**	**0.030**	**0.163**	**0.855**
		**Time:ever_fumigantTRUE**	**0.015**	**0.253**	**0.953**
**FoxInsight**	UPDRS II	Baseline age	-0.008	0.046	0.861
		Sex (male)	1.507	0.945	0.112
		Disease duration	0.822	0.162	7.61e-07
		**Time**	**0.868**	**0.241**	**4.77e-04**
		**ever_herbicideTRUE**	**1.948**	**1.057**	**0.067**
		**ever_fungicideTRUE**	**1.297**	**1.232**	**0.294**
		**ever_insecticideTRUE**	**-0.467**	**1.123**	**0.678**
		**ever_fumigantTRUE**	**0.653**	**1.217**	**0.592**
		**ever_rodenticideTRUE**	**2.249**	**1.151**	**0.052**
		**Time:ever_herbicideTRUE**	**0.321**	**0.208**	**0.125**
		**Time:ever_fungicideTRUE**	**-0.083**	**0.244**	**0.733**

	**Time:ever_insecticideTRUE**	**-0.414**	**0.227**	**0.071**
	**Time:ever_fumigantTRUE**	**0.404**	**0.249**	**0.108**
	**Time:ever_rodenticideTRUE**	**-0.073**	**0.233**	**0.754**
PDAQ-15	Baseline age	0.088	0.064	0.173
	Sex (male)	-0.714	1.309	0.586
	Disease duration	-0.666	0.227	0.004
	**Time**	**-1.284**	**0.262**	**2.55e-06**
	**ever_herbicideTRUE**	**-2.507**	**1.424**	**0.080**
	**ever_fungicideTRUE**	**0.057**	**1.651**	**0.972**
	**ever_insecticideTRUE**	**1.137**	**1.511**	**0.453**
	**ever_fumigantTRUE**	**-1.621**	**1.632**	**0.322**
	**ever_rodenticideTRUE**	**0.682**	**1.540**	**0.658**
	**Time:ever_herbicideTRUE**	**-0.047**	**0.228**	**0.838**
	**Time:ever_fungicideTRUE**	**0.297**	**0.263**	**0.260**
	**Time:ever_insecticideTRUE**	**0.291**	**0.247**	**0.241**
	**Time:ever_fumigantTRUE**	**-0.102**	**0.271**	**0.706**
	**Time:ever_rodenticideTRUE**	**0.326**	**0.250**	**0.195**
GDS	Baseline age	-0.022	0.011	0.048
	Sex (male)	0.391	0.226	0.086
	Disease duration	0.013	0.040	0.743
	**Time**	**0.114**	**0.079**	**0.151**
	**ever_herbicideTRUE**	**-0.080**	**0.234**	**0.732**
	**ever_fungicideTRUE**	**0.359**	**0.269**	**0.183**
	**ever_insecticideTRUE**	**0.135**	**0.250**	**0.589**
	**ever_fumigantTRUE**	**0.127**	**0.276**	**0.645**
	**ever_rodenticideTRUE**	**0.365**	**0.254**	**0.153**
	**Time:ever_herbicideTRUE**	**0.015**	**0.068**	**0.819**
	**Time:ever_fungicideTRUE**	**0.078**	**0.079**	**0.322**
	**Time:ever_insecticideTRUE**	**0.011**	**0.074**	**0.879**
	**Time:ever_fumigantTRUE**	**0.059**	**0.081**	**0.473**
	**Time:ever_rodenticideTRUE**	**-0.131**	**0.075**	**0.083**

Fixed-effect coefficients from linear mixed-effects models fitted to each outcome scale within the exposed subgroup of the propensity score–matched cohorts. Each model specified the scale total score as the dependent variable, with baseline age, sex (male), disease duration at baseline, time (years since baseline assessment), binary indicators for ever-exposure to each specific pesticide functional class, and the corresponding time × class interaction terms as fixed effects. Patient ID was included as a random effect with both random intercepts and random slopes for time. Models were estimated using restricted maximum likelihood (REML). Four pesticide functional classes were entered simultaneously in PPMI Online: ever_herbicideTRUE, ever_fungicideTRUE, ever_insecticideTRUE, and ever_fumigantTRUE (bolded), each coded as a binary variable indicating whether the participant reported ever-exposure to that class. In Fox Insight, an additional class (ever_rodenticideTRUE) was available. All class indicators were included in the same model; coefficients therefore represent the independent association of each class adjusted for co-exposure to the others. The corresponding time × class interaction terms (bolded, underlined) represent the estimated modification of the annual rate of change associated with each specific class. Intercept terms were omitted for clarity. SE, standard error. UPDRS II, Movement Disorder Society–Unified Parkinson's Disease Rating Scale

Part II; GDS, Geriatric Depression Scale; PDAQ-15, Penn Parkinson's Daily Activities Questionnaire-15; ESS, Epworth Sleepiness Scale; PDSS-2, Parkinson's Disease Sleep Scale-2; PAS, Parkinson Anxiety Scale. ESS, PAS, and PDSS-2 were assessed in PPMI Online only; Fox Insight included UPDRS II, PDAQ-15, and GDS.

Outcome	ICD-10 code	Description
**Motor function**	Z74.0	Reduced mobility
	Z74.1	Need for assistance with personal care
	Z74.2	Need for assistance at home and no other household member able to render care
	Z74.3	Need for continuous supervision
**Dementia**	F02.80	Dementia in other diseases classified elsewhere, unspecified severity, without behavioral disturbance, psychotic disturbance, mood disturbance, and anxiety
	F02.81	Dementia in other diseases classified elsewhere, unspecified severity, with behavioral disturbance
**Depression**	F06.31	Mood disorder due to known physiological condition with depressive features
	F06.32	Mood disorder due to known physiological condition with major depressive-like episode
	F32	Depressive episode
	F33	Major depressive disorder, recurrent
**Anxiety**	F06.4	Anxiety disorder due to known physiological condition
	F41.0	Panic disorder [episodic paroxysmal anxiety]
	F41.1	Generalized anxiety disorder
	F41.9	Anxiety disorder, unspecified
**Sleep disorder**	G47.1	Hypersomnia
	R40.0	Somnolence

Supplementary Table 8. ICD-10 Outcome Definitions for the TriNetX Triangulation Analysis. ICD-10 diagnostic codes used to define five symptom domain outcomes in the TriNetX analysis. Motor function codes (Z74.0–Z74.3) capture severe functional dependence requiring personal care or continuous supervision, corresponding to a later disease stage than the moderate motor impairment threshold (MDS-UPDRS Part II > 12) used in the primary analysis. Depression codes include F06.31 and F06.32 (mood disorder due to known physiological condition), which in PD patients may partly reflect non-motor manifestations of the disease itself rather than an independent comorbidity. Sleep disorder codes capture hypersomnia-related diagnoses only and do not cover nocturnal sleep quality dimensions; they therefore partially correspond to the ESS (daytime sleepiness) but not to the PDSS-2 (nocturnal sleep disturbance) used in the primary analysis. ESS, Epworth Sleepiness Scale; MDS-UPDRS, Movement Disorder Society–Unified Parkinson's Disease Rating Scale; PD, Parkinson's disease; PDSS-2, Parkinson's Disease Sleep Scale-2.

	Before matching				After matching			
**Characteristic**	PD-pesticide	PD+ pesticide	*p*	SMD	PD- pesticide	PD+ pesticide	*p*	SMD
Sample size	511,921	776	-	-	755	755	-	-
Baseline age	72.6 (10.8)	71.7 (9.2)	0.024	0.088	71.8 (9.0)	71.7 (9.2)	0.953	0.009
Sex							0.958	0.005
Male	285,016(57.8)	596 (78.9)	<0.001	0.467	596 (78.9)	596 (78.9)	1	<0.001
Female	207,706(42.1)	158 (20.9)	<0.001	0.468	158 (20.9)	158 (20.9)	1	<0.001
Nicotine dependence	34,839 (7.1)	183 (24.2)	<0.001	0.486	184 (24.4)	183 (24.2)	0.952	0.003
Alcohol dependence	4,567 (0.9)	23 (3.0)	<0.001	0.152	23 (3.0)	23 (3.0)	1	<0.001
Race/Ethnicity								
Asian	34,625 (7.0)	69 (9.1)	0.023	0.078	69 (9.1)	69 (9.1)	1	<0.001
Black/AA	21,754 (4.4)	35 (4.6)	0.764	0.011	35 (4.6)	35 (4.6)	1	<0.001
White	309,034(62.7)	559 (74.0)	<0.001	0.246	558 (73.9)	559 (74.0)	0.953	0.003
AI/AN	1,216 (0.2)	10 (1.3)	<0.001	0.122	10 (1.3)	10 (1.3)	1	<0.001
NHOPI	1,075 (0.2)	10 (1.3)	<0.001	0.127	10 (1.3)	10 (1.3)	1	<0.001
H/L	18,984 (3.8)	35 (4.6)	0.262	0.039	35 (4.6)	35 (4.6)	1	<0.001
NHL	299,390(60.7)	598 (79.2)	<0.001	0.412	598 (79.2)	598 (79.2)	1	<0.001
Other	14,145 (2.9)	11 (1.5)	0.020	0.097	12 (1.6)	11 (1.5)	0.834	0.011

Supplementary Table 9. Baseline characteristics of PD patients with and without ICD-coded pesticide exposure before and after propensity score matching in TriNetX analysis. Continuous variables are presented as mean (standard deviation); categorical variables are presented as n (%). In the TriNetX analysis, propensity scores were estimated by logistic regression and 1:1 nearest-neighbour matching was performed on age, sex, race, ethnicity, history of nicotine dependence (Z87.891), and history of alcohol dependence (F10.2). Matching was performed using 1:1 nearest-neighbour matching without replacement, with a caliper of 0.2 standard deviations of the logit of the propensity score. Balance was assessed using the standardized mean difference (SMD), with |SMD| < 0.1 considered acceptable. p-values for continuous variables were derived from two-sample t-tests; p-values for categorical variables were derived from chi-squared tests. SMD, standardized mean difference; PD−pesticide, patients without occupational pesticide exposure; PD+pesticide, patients with ICD-coded pesticide exposure. *Abbreviations: PD, Parkinson's disease; AA, African American; AI/AN: American Indian or Alaska Native; NHOPI, Native Hawaiian or Other Pacific Islander; H/L, Hispanic or Latino; NHL, Not Hispanic or Latino.*

Event	Patients with outcome, N (%)					
	+pesticide	-pesticide	RD (95%CI)	RR (95%CI)	OR (95%CI)	*p*-value
Motor function	43 (5.7)	23 (3.0)	0.026(0.006,0.047)	1.870(1.138, 3.071)	1.922(1.146, 3.222)	0.012
Dementia	153 (20.3)	106 (14.0)	0.062(0.024,0.100)	1.443(1.151, 1.810)	1.556(1.186, 2.041)	0.001
Depression	274 (36.3)	173 (22.9)	0.134(0.088,0.179)	1.584(1.348, 1.861)	1.916(1.530, 2.401)	<0.001
Anxiety	248 (32.8)	158 (20.9)	0.119(0.075,0.164)	1.570(1.321, 1.864)	1.848(1.466, 2.331)	<0.001
Sleep disorder	59 (7.8)	39 (5.2)	0.026(0.002,0.051)	1.513(1.023, 2.238)	1.556(1.025, 2.363)	0.037

Supplementary Table 10. Measure of Association Analysis for Five Symptom Domains After Propensity Score Matching. Risk of five PD-related symptom domains in the pesticide-exposed (+pesticide) versus unexposed (−pesticide) cohorts after 1:1 propensity score matching. "+pesticide" denotes PD patients with a documented pesticide toxicity event or exposure (T60 or Z77.098); "−pesticide" denotes PD patients without these codes. Outcome events were defined by ICD-10 codes: motor function (Z74.0–Z74.3), dementia (F02.80, F02.81), depression (F06.31, F06.32, F32, F33), anxiety (F06.4, F41.0, F41.1, F41.9), and sleep disorder (G47.1, R40.0). *Abbreviations: RD, risk difference; RR, risk ratio; OR, odds ratio; CI, confidence interval.*

Covariate	Coef.	SE	HR (95%CI)	*p* value
**Pesticide exposure**	0.524	0.093	1.689 (1.407, 2.027)	<0.001
Male	0.079	0.011	1.082 (1.059, 1.106)	<0.001
Age at Index	0.031	0.001	1.031 (1.030, 1.033)	<0.001
Dementia (F02.80)	0.222	0.031	1.249 (1.175, 1.327)	<0.001
Mood disorder (F06.3)	0.692	0.077	1.999 (1.719, 2.323)	<0.001
Depression (F32)	0.441	0.017	1.554 (1.502, 1.607)	<0.001
Anxiety (F41)	0.327	0.017	1.386 (1.340, 1.434)	<0.001
Nicotine dependence (F17)	0.415	0.024	1.514 (1.444, 1.587)	<0.001

Supplementary Table 11. Cox Proportional Hazards Model for the Motor Function Milestone Event. Cox regression analysis evaluating the association between pesticide exposure and the risk of reaching a motor function milestone (first occurrence of ICD-10: Z74.0-Z74.3) after PD diagnosis. The model was fitted in the full cohort (+pesticide, n = 755; −pesticide, n = 493,163) without propensity score matching. Pesticide exposure (bolded) was entered as the main predictor. Covariates included sex, age at index, and five comorbidity diagnoses: dementia (F02.80), mood disorder (F06.3), depressive episode (F32), anxiety disorder (F41), and nicotine dependence (F17). *Abbreviations: Coef., regression coefficient; SE, standard error; HR, hazard ratio; CI, confidence interval.*

Supplementary Table 12. Pesticide-exposure associations before and after adjustment for anti-parkinsonian medication.

Cohort	Scale	Effect	Primary	+ baseline med	+ time-varying med
PPMI	UPDRS-II	Severity	1.97 (1.33, 2.60)	1.99 (1.35, 2.62)	1.97 (1.34, 2.61)
		Progression	0.03 (-0.18, 0.24)	0.04 (-0.17, 0.25)	0.05 (-0.16, 0.26)
PPMI	PDAQ-15	Severity	-2.40 (-3.23, -1.57)	-2.37 (-3.21, -1.53)	-2.36 (-3.20, -1.53)
		Progression	-0.00 (-0.31, 0.31)	0.02 (-0.29, 0.33)	0.00 (-0.31, 0.32)
PPMI	GDS	Severity	0.33 (0.20, 0.47)	0.32 (0.19, 0.46)	0.34 (0.20, 0.47)
		Progression	0.07 (0.01, 0.13)	0.07 (0.00, 0.13)	0.07 (0.01, 0.13)
PPMI	PAS	Severity	2.00 (1.35, 2.64)	1.97 (1.32, 2.61)	1.99 (1.35, 2.63)
		Progression	0.18 (-0.03, 0.40)	0.18 (-0.03, 0.39)	0.20 (-0.01, 0.41)
PPMI	ESS	Severity	0.63 (0.24, 1.03)	0.65 (0.25, 1.05)	0.59 (0.19, 0.98)
		Progression	-0.02 (-0.20, 0.17)	-0.02 (-0.20, 0.17)	-0.04 (-0.23, 0.14)
PPMI	PDSS-2	Severity	2.24 (1.50, 2.97)	2.26 (1.52, 2.99)	2.20 (1.47, 2.94)
		Progression	-0.17 (-0.51, 0.16)	-0.16 (-0.50, 0.17)	-0.15 (-0.49, 0.18)
FoxInsight	UPDRS-II	Severity	1.02 (-0.21, 2.25)	1.05 (-0.18, 2.27)	1.02 (-0.21, 2.25)
		Progression	-0.27 (-0.52, -0.01)	-0.27 (-0.52, -0.01)	-0.27 (-0.52, -0.02)
FoxInsight	PDAQ-15	Severity	-1.58 (-3.12, -0.05)	-1.61 (-3.14, -0.08)	-1.58 (-3.12, -0.05)
		Progression	0.14 (-0.14, 0.41)	0.14 (-0.14, 0.41)	0.15 (-0.13, 0.42)
FoxInsight	GDS	Severity	-0.10 (-0.47, 0.27)	-0.10 (-0.47, 0.28)	-0.09 (-0.46, 0.29)
		Progression	-0.06 (-0.18, 0.07)	-0.06 (-0.18, 0.07)	-0.05 (-0.18, 0.07)

Panel A. Fixed-effect estimates from linear mixed-effects models evaluating whether anti-parkinsonian medication accounts for the association between occupational pesticide exposure and symptom burden. Because anti-parkinsonian medication lies on the hypothesized causal pathway (exposure → greater symptom severity → more treatment) and is therefore a mediator in the directed acyclic graph, it was excluded from the primary model; here it is added only as a sensitivity analysis. Models used the primary LMM specification (scale total score as the dependent variable; fixed effects for baseline age, sex, disease duration at baseline, time, exposure, and time × exposure; random intercepts and slopes for time per participant), with one additional fixed-

effect covariate: either baseline use of any anti-parkinsonian medication or time-varying (visit-level) use of any anti-parkinsonian medication. Visit-level medication status was derived from the overall current-medication indicator and the brand-level medication checkboxes, joined to each symptom visit by visit identifier in PPMI Online and to the nearest prior medication record within one year in Fox Insight (no future medication information was used); medication coverage was 97–99% of PPMI Online and 99.7% of Fox Insight symptom visits, and 84% of PPMI Online participants were taking medication at baseline. A levodopa-equivalent daily dose could not be derived because neither cohort recorded medication dose, frequency, or units. For each cohort and scale, the table reports the exposure main effect ("Severity") and the time × exposure interaction ("Progression"), each as β with 95% CI in raw scale points, under the primary (unadjusted), baseline-medication-adjusted, and time-varying-medication-adjusted models. The exposure estimates are essentially unchanged by medication adjustment (for example, PPMI Online UPDRS-II 1.97 unadjusted versus 1.97 with time-varying medication; PDAQ-15 −2.40 versus −2.36), indicating that the association with symptom burden is not explained by differences in treatment status..

Cohort	Treatment (baseline)	Exposed	Unexposed	SMD
PPMI	Any anti-PD medication	83.3%	82.9%	0.01
PPMI	Levodopa	75.9%	74.4%	0.036
PPMI	Dopamine agonist	13.8%	16.1%	-0.065
PPMI	MAO-B inhibitor	13.1%	12.9%	0.005
PPMI	DBS (ever)	6.0%	5.3%	0.031
FoxInsight	Any anti-PD medication	93.2%	93.9%	-0.027
FoxInsight	Levodopa	77.0%	73.5%	0.082
FoxInsight	Dopamine agonist	32.4%	26.5%	0.13
FoxInsight	MAO-B inhibitor	26.5%	28.6%	-0.045
FoxInsight	DBS (ever)	16.2%	17.2%	-0.026

Panel B. Baseline treatment status by exposure group within the propensity score–matched cohort, summarising the prevalence of any anti-parkinsonian medication and of the major drug classes (levodopa, dopamine agonist, MAO-B inhibitor) and of DBS, as the percentage of participants in each exposure group, with the standardized mean difference between groups. Available treatment indicators (any anti-Parkinsonian medication, major drug classes, and DBS) were broadly similar between exposure groups after matching, with the largest difference for Fox Insight dopamine-agonist use (32.4% vs 26.5%, SMD 0.13); adjustment for these indicators produced little change in the estimates, although residual differences in medication intensity, adherence, and therapeutic response could not be excluded.

Supplementary Table 13. Inverse-probability-of-treatment-weighting (IPTW) analysis of the full cohorts versus the matched analysis.

Cohort	Scale	Effect	Primary (matched)	IPTW (full sample)
PPMI	UPDRS-II	Severity	1.97 (1.33, 2.60)	2.41 (1.94, 2.88)
		Progression	0.03 (-0.18, 0.24)	0.03 (-0.13, 0.20)
PPMI	PDAQ-15	Severity	-2.40 (-3.23, -1.57)	-2.93 (-3.53, -2.33)
		Progression	-0.00 (-0.31, 0.31)	-0.07 (-0.30, 0.15)
PPMI	GDS	Severity	0.33 (0.20, 0.47)	0.36 (0.26, 0.46)
		Progression	0.07 (0.01, 0.13)	0.04 (-0.01, 0.09)
PPMI	PAS	Severity	2.00 (1.35, 2.64)	2.26 (1.78, 2.74)
		Progression	0.18 (-0.03, 0.40)	0.16 (-0.01, 0.32)
PPMI	ESS	Severity	0.63 (0.24, 1.03)	0.83 (0.56, 1.11)
		Progression	-0.01 (-0.20, 0.17)	-0.03 (-0.17, 0.11)
PPMI	PDSS-2	Severity	2.24 (1.50, 2.97)	2.81 (2.27, 3.35)
		Progression	-0.17 (-0.51, 0.16)	-0.10 (-0.36, 0.15)
FoxInsight	UPDRS-II	Severity	1.02 (-0.20, 2.25)	1.55 (0.70, 2.40)
		Progression	-0.27 (-0.52, -0.01)	-0.10 (-0.26, 0.06)
FoxInsight	PDAQ-15	Severity	-1.58 (-3.12, -0.05)	-2.60 (-3.64, -1.57)
		Progression	0.14 (-0.14, 0.41)	-0.03 (-0.20, 0.14)
FoxInsight	GDS	Severity	-0.10 (-0.47, 0.28)	0.17 (-0.07, 0.42)
		Progression	-0.06 (-0.18, 0.07)	-0.01 (-0.08, 0.07)

Fixed-effect estimates from linear mixed-effects models re-fitted in the full eligible cohorts (PPMI Online, n = 7,190; Fox Insight, n = 2,298) using inverse-probability-of-treatment weighting, conducted to address whether discarding unmatched participants in 1:1 matching affected the results and to use all available data. Stabilized weights targeting the average treatment effect were computed from the same propensity-score (logistic-regression) model used for matching—covariates: baseline age, sex, race, ethnicity, education, disease duration at baseline, family history of PD, regular smoking history, and regular coffee-consumption history—and were trimmed at the 1st and 99th percentiles. The weighted models used the primary LMM specification (exposure main effect and time × exposure interaction; baseline age, sex, and disease duration as fixed-effect covariates; random intercepts and slopes for time). For each cohort and scale the table reports

the exposure main effect (“Severity”) and the time × exposure interaction (“Progression”) as β with 95% CI, alongside the corresponding primary (matched) estimate. IPTW reproduced the matched estimates and was, if anything, slightly stronger (for example, PPMI Online UPDRS-II 2.41, 95% CI 1.94 to 2.88; the Fox Insight UPDRS-II association strengthened from 1.02 in the matched analysis to 1.55, 95% CI 0.70 to 2.40). Weighting diagnostics: stabilized weights ranged 0.55–2.16 (mean 1.0) in PPMI Online and 0.37–1.94 (mean 1.0) in Fox Insight; after weighting all covariates were balanced (maximum weighted standardized mean difference 0.044 in PPMI Online and 0.055 in Fox Insight, reduced from 0.54 and 0.41 before weighting); and the effective sample sizes (exposed/unexposed) were 843/6,073 in PPMI Online and 359/1,859 in Fox Insight, indicating little loss of precision. Together these results indicate that the associations are not an artefact of the matched sample.

Supplementary Table 14. Missing-data sensitivity analyses.

Covariate	PPMI Online, n (%)	Fox Insight, n (%)
Baseline age	0 (0%)	0 (0%)
Disease duration	0 (0%)	0 (0%)
Education	1116 (15.5%)	0 (0%)
Family history of PD	1030 (14.3%)	270 (11.7%)
Regular smoking	1593 (22.2%)	36 (1.6%)
Regular coffee	2561 (35.6%)	573 (24.9%)
Race	144 (2%)	17 (0.7%)
Ethnicity	220 (3.1%)	0 (0%)

Panel A. The number and percentage of participants with a missing or "prefer not to answer / do not know" value for each propensity-score covariate, computed in the full eligible cohorts (PPMI Online, n = 7,190; Fox Insight, n = 2,298), reported to justify the missing-data approach used in the primary analysis. The largest proportions were for regular caffeinated-coffee history (35.6% in PPMI Online and 24.9% in Fox Insight) and regular smoking history (22.2% in PPMI Online); baseline age and disease duration were complete. Because these items include explicit non-response options and are therefore unlikely to be missing at random, the primary propensity-score model retained participants by using an explicit missing-indicator category rather than excluding them.

Cohort	Scale	Effect	Primary	Complete-case	MICE (pooled)
PPMI	UPDRS-II	Severity	1.97 (1.33, 2.60)	1.92 (0.92, 2.92)	2.19 (1.48, 2.90)
		Progression	0.03 (-0.18, 0.24)	-0.28 (-0.69, 0.13)	-0.00 (-0.22, 0.21)
PPMI	PDAQ-15	Severity	-2.40 (-3.23, -1.57)	-2.33 (-3.77, -0.90)	-2.54 (-3.55, -1.53)
		Progression	-0.00 (-0.31, 0.31)	0.02 (-0.63, 0.67)	-0.09 (-0.45, 0.27)
PPMI	GDS	Severity	0.33 (0.20, 0.47)	0.24 (0.02, 0.46)	0.32 (0.17, 0.47)
		Progression	0.07 (0.01, 0.13)	0.17 (0.03, 0.29)	0.04 (-0.03, 0.11)

PPMI	PAS	Severity	2.00 (1.35, 2.64)	2.63 (1.56, 3.69)	2.12 (1.37, 2.87)
		Progression	0.18 (-0.03, 0.40)	0.34 (-0.08, 0.76)	0.22 (-0.04, 0.47)
PPMI	ESS	Severity	0.63 (0.24, 1.03)	0.35 (-0.25, 0.95)	0.81 (0.40, 1.23)
		Progression	-0.01 (-0.20, 0.17)	-0.26 (-0.68, 0.16)	0.01 (-0.18, 0.19)
PPMI	PDSS-2	Severity	2.24 (1.50, 2.97)	2.93 (1.65, 4.21)	2.64 (1.79, 3.49)
		Progression	-0.17 (-0.51, 0.16)	-0.48 (-1.18, 0.21)	-0.14 (-0.53, 0.25)
FoxInsight	UPDRS-II	Severity	1.02 (-0.20, 2.25)	1.16 (-0.19, 2.52)	1.25 (0.02, 2.49)
		Progression	-0.27 (-0.52, -0.01)	-0.12 (-0.36, 0.11)	-0.17 (-0.39, 0.06)
FoxInsight	PDAQ-15	Severity	-1.58 (-3.12, -0.05)	-1.90 (-3.56, -0.25)	-1.98 (-3.54, -0.42)
		Progression	0.14 (-0.14, 0.41)	-0.07 (-0.33, 0.18)	0.09 (-0.17, 0.35)
FoxInsight	GDS	Severity	-0.10 (-0.47, 0.28)	0.14 (-0.19, 0.46)	0.11 (-0.25, 0.48)
		Progression	-0.06 (-0.18, 0.07)	0.07 (-0.02, 0.16)	-0.04 (-0.15, 0.08)

Panel B. Robustness of the exposure estimates to the missing-data approach, from two re-analyses that each repeated propensity-score estimation, matching, and outcome modelling under a different missing-data assumption: (i) a complete-case analysis restricted to participants with non-missing smoking, coffee, and family-history information (re-matched); and (ii) multiple imputation by chained equations with 20 imputed datasets, in which matching and outcome modelling were performed within each imputed dataset and estimates were pooled by Rubin's rules. Outcome models used the primary LMM specification. For each cohort and scale the table reports the exposure main effect (“Severity”) and the time × exposure interaction (“Progression”) as β with 95% CI under the primary, complete-case, and multiple-imputation analyses. Both approaches gave estimates materially identical to the primary analysis (for example, PPMI Online UPDRS-II 1.97 primary versus 1.92 complete-case and 2.19 multiple imputation), indicating that the findings are not driven by the handling of missing covariate data.

Supplementary Table 15. Representativeness of the matched cohort (matched vs. excluded participants).

Cohort	Exposure group	N eligible	N retained	% retained
PPMI	Exposed	1065	1059	99.4%
PPMI	Unexposed	6125	1059	17.3%
FoxInsight	Exposed	419	400	95.5%
FoxInsight	Unexposed	1879	400	21.3%

Panel A. The number of eligible participants and the number and percentage retained after 1:1 nearest-neighbour matching, separately by cohort and exposure group, reported to characterise which participants were excluded by matching. Matching retained almost all exposed participants (99.4% in PPMI Online and 95.5% in Fox Insight) and down-sampled only the surplus unexposed controls (17.3% and 21.3% retained, respectively), so inference about the exposed group—the central comparison of the study—was essentially unaffected by the reduction in sample size.

Cohort	Variable	Retained controls	Excluded controls	SMD
PPMI	N	1059	5066	
PPMI	Age, mean (SD)	67.2 (8.9)	67.6 (8.8)	-0.044
PPMI	Disease duration, mean (SD)	2.86 (2.46)	2.73 (2.43)	0.052
PPMI	Education, mean (SD)	15.8 (3.5)	16.5 (3.7)	-0.208
PPMI	Male, %	78.7	49	0.649
PPMI	Smoking, %	39.5	31	0.179
PPMI	Coffee, %	81.3	78.1	0.082
PPMI	Family history, %	23.5	27.4	-0.089
PPMI	White, %	94.8	94.5	0.013
PPMI	Baseline UPDRS-II, mean (SD)	9.89 (7.10)	9.17 (6.72)	0.104
FoxInsight	N	400	1479	
FoxInsight	Age, mean (SD)	63.8 (9.7)	64.2 (8.8)	-0.038

FoxInsight	Disease duration, mean (SD)	3.19 (2.54)	3.16 (2.58)	0.014
FoxInsight	Education (low), %	0.5	0.7	-0.031
FoxInsight	Education (medium), %	35.8	30.4	0.113
FoxInsight	Education (high), %	63.5	68.6	-0.107
FoxInsight	Male, %	68.5	42.7	0.538
FoxInsight	Smoking, %	34.3	29.5	0.102
FoxInsight	Coffee, %	77.6	75.2	0.057
FoxInsight	Family history, %	30.8	27	0.084
FoxInsight	White, %	96.2	96.9	-0.035
FoxInsight	Baseline UPDRS-II, mean (SD)	10.23 (6.89)	10.03 (6.96)	0.028

Panel B. A comparison of the unexposed controls retained after matching with those excluded by matching, to assess whether matching produced a prognostically selected control subset. Continuous variables are shown as mean (SD) and categorical variables as percentages, with the standardized mean difference between retained and excluded controls; the comparison includes age, baseline disease duration, education, sex, smoking, coffee consumption, family history of PD, race, and the baseline MDS-UPDRS Part II score (a proxy for baseline motor severity). The excluded and retained controls were similar in age, baseline disease duration, and baseline motor severity and differed mainly on the variables used for matching (sex and smoking history), indicating that matching did not preferentially retain a more or less severely affected control subgroup. Read together with the full-cohort IPTW analysis (Supplementary Table 13), which discards no participants and reproduces the matched estimates, these results provide reassurance about the representativeness and external validity of the matched analyses.

Supplementary Table 16. Sensitivity to left truncation: patients with less than five years' baseline disease duration.

Cohort	Scale	Effect	Primary	Disease duration <5y
PPMI	UPDRS-II	Severity	1.97 (1.33, 2.60)	2.19 (1.51, 2.87)
		Progression	0.03 (-0.18, 0.24)	-0.00 (-0.25, 0.24)
PPMI	PDAQ-15	Severity	-2.40 (-3.23, -1.57)	-2.86 (-3.76, -1.96)
		Progression	-0.00 (-0.31, 0.31)	0.15 (-0.19, 0.50)
PPMI	GDS	Severity	0.33 (0.20, 0.47)	0.38 (0.23, 0.53)
		Progression	0.07 (0.01, 0.13)	0.07 (0.00, 0.14)
PPMI	PAS	Severity	2.00 (1.35, 2.64)	2.13 (1.42, 2.84)
		Progression	0.18 (-0.03, 0.40)	0.18 (-0.07, 0.43)
PPMI	ESS	Severity	0.63 (0.24, 1.03)	0.67 (0.23, 1.11)
		Progression	-0.01 (-0.20, 0.17)	-0.02 (-0.23, 0.19)
PPMI	PDSS-2	Severity	2.24 (1.50, 2.97)	2.57 (1.75, 3.39)
		Progression	-0.17 (-0.51, 0.16)	-0.11 (-0.49, 0.28)
FoxInsight	UPDRS-II	Severity	1.02 (-0.20, 2.25)	0.76 (-0.59, 2.11)
		Progression	-0.27 (-0.52, -0.01)	-0.34 (-0.63, -0.06)
FoxInsight	PDAQ-15	Severity	-1.58 (-3.12, -0.05)	-1.21 (-2.82, 0.40)
		Progression	0.14 (-0.14, 0.41)	0.07 (-0.23, 0.38)
FoxInsight	GDS	Severity	-0.10 (-0.47, 0.28)	-0.03 (-0.46, 0.41)
		Progression	-0.06 (-0.18, 0.07)	-0.05 (-0.20, 0.10)

Fixed-effect estimates from linear mixed-effects models re-fitted after restricting each cohort to participants with less than five years of disease duration at baseline, conducted to assess whether the baseline-severity associations were an artefact of variable disease duration at cohort entry (left truncation in a prevalent-case design). Models used the primary LMM specification (exposure main effect and time × exposure interaction; baseline age, sex, and disease duration as covariates; random intercepts and slopes for time). For each cohort and scale the table reports the exposure main effect ("Severity") and the time × exposure interaction ("Progression") as β with 95% CI, alongside the corresponding primary estimate from the full matched cohort. Restricting to early disease duration left the severity associations unchanged or slightly stronger (for example, PPMI Online UPDRS-II 1.97 → 2.19; PDAQ-15 −2.40 → −2.86; PDSS-2 2.24 → 2.57), indicating

that the baseline-severity findings are not explained by differences in disease duration among enrolled participants; this analysis cannot, however, address selection of patients who never entered the cohort.

Supplementary Table 17. Milestone (Cox) sensitivity: adjustment for baseline severity and delayed-entry models.

Cohort	Endpoint	Events	N	Primary HR (95% CI)	+ Baseline score	Delayed-entry (duration)
PPMI	UPDRS2 > 12	408	1378	1.22 (1.00, 1.48)	1.09 (0.89, 1.32)	1.21 (0.99, 1.47)
PPMI	PDAQ-15 < 37	106	1909	1.28 (0.87, 1.87)	0.81 (0.55, 1.19)	1.30 (0.88, 1.90)
PPMI	GDS > 5	483	961	1.02 (0.85, 1.22)	1.02 (0.85, 1.22)	1.03 (0.86, 1.23)
PPMI	ESS > 10	190	1288	1.59 (1.19, 2.13)	1.40 (1.05, 1.87)	1.61 (1.20, 2.14)
PPMI	PAS total > 13	405	1429	1.28 (1.05, 1.55)	1.17 (0.96, 1.42)	1.30 (1.07, 1.58)
FoxInsight	UPDRS2 > 12	208	435	1.33 (1.01, 1.75)	1.11 (0.84, 1.47)	1.36 (1.04, 1.79)
FoxInsight	PDAQ-15 < 37	90	629	1.26 (0.83, 1.91)	1.22 (0.80, 1.85)	1.28 (0.84, 1.93)
FoxInsight	GDS > 5	176	302	0.87 (0.64, 1.17)	0.87 (0.65, 1.18)	0.88 (0.66, 1.19)

Hazard ratios from multivariable Cox proportional hazards models for the clinically meaningful milestone events, conducted to evaluate whether the milestone associations reflect greater baseline symptom burden in the exposed group rather than a faster post-baseline progression rate, and whether they are sensitive to left truncation. As in the primary milestone analysis (Supplementary Table 4), models were fitted within the propensity score–matched cohorts, restricted to participants who had not reached the corresponding threshold at baseline, with pesticide exposure as the primary predictor and baseline age, sex, and disease duration as covariates; milestone thresholds were MDS-UPDRS Part II > 12 (motor), PDAQ-15 < 37 (cognitive), GDS > 5 (depression), ESS > 10 (daytime sleepiness), and PAS > 13 (anxiety). The table reports, for each endpoint, the number of events and participants and the exposure hazard ratio (95% CI) under three models: the primary model; a model additionally adjusted for the baseline score of the corresponding scale (equivalently, the baseline distance to the clinical threshold; because the baseline score may itself lie on the causal pathway, this is an explanatory sensitivity analysis, not the primary model); and a delayed-entry (left-truncation–aware) Cox model using disease duration as the time scale, in which participants entered the risk set at their baseline disease duration. Additional adjustment for baseline severity attenuated the hazard ratios toward the null (PPMI Online motor 1.22 to 1.09; anxiety 1.28 to 1.17; Fox Insight motor 1.33 to 1.11), consistent with a substantial contribution of greater baseline symptom burden to earlier milestone attainment in the exposed group rather than clearly faster progression; the daytime-sleepiness milestone remained elevated (1.59 to 1.40), suggesting a component not fully explained by baseline level. The delayed-entry models yielded materially similar hazard ratios among enrolled participants (for example, motor milestone 1.21 in PPMI Online and 1.36 in Fox Insight; daytime sleepiness 1.61), suggesting that the milestone associations were not driven by the choice of follow-up time scale.

Supplementary Table 18. Follow-up and attrition by exposure group (matched UPDRS-II cohort).

Cohort	Exposure	N	Mean visits	Mean follow-up (y)	≥ 2 y, %	Baseline only, %
PPMI	Unexposed	1057	5.32	1.67	45.3	14
PPMI	Exposed	1054	5.23	1.67	42.6	12.6
FoxInsight	Unexposed	343	7.51	4.13	73.2	7.3
FoxInsight	Exposed	339	7.63	4.16	70.8	12.1

Panel A. A comparison of follow-up and study attrition between exposed and unexposed participants in the propensity score–matched MDS-UPDRS Part II cohort, conducted to assess whether differential loss to follow-up could bias the longitudinal (slope) estimates. For each cohort and exposure group the table reports the number of participants, the mean number of completed visits, the mean follow-up duration (years), the percentage with at least two years of follow-up, and the percentage completing only a baseline assessment. Differences between exposure groups were tested in models adjusted for age, sex, baseline disease duration, and baseline UPDRS-II score: a linear model for follow-up duration and a logistic model for completing only a baseline assessment. Follow-up was similar between groups in both cohorts (PPMI Online: mean 1.67 versus 1.67 years; adjusted difference 0.05 years, p = 0.29; Fox Insight: 4.16 versus 4.13 years), as was the proportion completing only a baseline assessment (PPMI Online 12.6% versus 14.0%; adjusted odds ratio 0.80, p = 0.087), with a single exception—a higher proportion of single-assessment participants among exposed Fox Insight participants (12.1% versus 7.3%; adjusted odds ratio 1.70, p = 0.049). Overall there was little evidence of differential attrition that would materially bias the progression estimates.

Cohort	Follow-up difference, y (95% CI)	p	Baseline-only OR (95% CI)	p
PPMI	0.05 (-0.04, 0.15)	0.286	0.80 (0.62, 1.03)	0.087
FoxInsight	0.17 (-0.24, 0.58)	0.42	1.70 (1.00, 2.88)	0.049

Panel B. Exposed-vs-unexposed comparison adjusted for age, sex, baseline disease duration, and baseline UPDRS-II score — a linear model for follow-up duration and a logistic model for completing only a baseline assessment. Follow-up duration did not differ by exposure; the proportion completing only a baseline assessment was similar in PPMI Online but nominally higher among exposed Fox Insight participants (odds ratio 1.70, 95% CI 1.00–2.88, p=0.049).

Supplementary Table 19. Exposure×time (progression) term across all sensitivity analyses.

Cohort	Scale	Primary	+Med(base)	+Med(TV)	IPTW	Compl.-case	MICE	Dur<5y
PPMI	UPDRS-II	0.03 (-0.18, 0.24)	0.04 (-0.17, 0.25)	0.05 (-0.16, 0.26)	0.03 (-0.13, 0.20)	-0.28 (-0.69, 0.13)	-0.00 (-0.22, 0.21)	-0.00 (-0.25, 0.24)
PPMI	PDAQ-15	-0.00 (-0.31, 0.31)	0.02 (-0.29, 0.33)	0.00 (-0.31, 0.32)	-0.07 (-0.30, 0.16)	0.02 (-0.63, 0.67)	-0.09 (-0.45, 0.27)	0.16 (-0.19, 0.50)
PPMI	GDS	0.07 (0.01, 0.13)	0.07 (0.00, 0.13)	0.07 (0.01, 0.13)	0.04 (-0.01, 0.09)	0.16 (0.03, 0.30)	0.04 (-0.03, 0.11)	0.07 (0.00, 0.14)
PPMI	PAS	0.18 (-0.03, 0.40)	0.18 (-0.03, 0.39)	0.20 (-0.01, 0.41)	0.16 (-0.01, 0.32)	0.34 (-0.08, 0.76)	0.22 (-0.04, 0.47)	0.18 (-0.07, 0.43)
PPMI	ESS	-0.02 (-0.20, 0.17)	-0.02 (-0.20, 0.17)	-0.04 (-0.23, 0.14)	-0.03 (-0.16, 0.11)	-0.26 (-0.68, 0.16)	0.00 (-0.18, 0.19)	-0.02 (-0.23, 0.19)
PPMI	PDSS-2	-0.17 (-0.51, 0.16)	-0.16 (-0.50, 0.17)	-0.15 (-0.49, 0.18)	-0.10 (-0.36, 0.16)	-0.48 (-1.18, 0.21)	-0.14 (-0.53, 0.25)	-0.11 (-0.49, 0.28)
FoxInsight	UPDRS-II	-0.27 (-0.52, -0.01)	-0.27 (-0.52, -0.01)	-0.27 (-0.52, -0.02)	-0.10 (-0.26, 0.06)	-0.12 (-0.36, 0.11)	-0.17 (-0.39, 0.06)	-0.34 (-0.63, -0.06)
FoxInsight	PDAQ-15	0.14 (-0.14, 0.41)	0.14 (-0.14, 0.41)	0.15 (-0.13, 0.42)	-0.03 (-0.20, 0.14)	-0.07 (-0.33, 0.18)	0.09 (-0.16, 0.35)	0.07 (-0.22, 0.38)
FoxInsight	GDS	-0.06 (-0.18, 0.07)	-0.06 (-0.18, 0.07)	-0.05 (-0.18, 0.07)	-0.01 (-0.08, 0.07)	0.07 (-0.02, 0.16)	-0.04 (-0.15, 0.08)	-0.05 (-0.20, 0.10)

The time × exposure interaction coefficient (the difference in annual progression rate between exposed and unexposed participants) for each cohort and scale, assembled across the primary analysis and all sensitivity analyses (medication-adjusted, full-cohort IPTW, complete-case, multiple imputation, and the less-than-five-year disease-duration subset), to show whether any evidence of accelerated progression was consistent across analytic choices. Each cell shows β per year with 95% CI from the corresponding linear mixed-effects model (primary LMM specification). Across analyses there was no consistent evidence that exposure accelerated progression. Two interaction terms were nominally significant in the primary analysis but isolated and inconsistent in direction and domain: a small positive PPMI Online GDS slope (0.07 points/year, 95% CI 0.01 to 0.13), which crossed zero under inverse-probability weighting and multiple imputation and was not accompanied by a depression milestone association; and a negative Fox Insight UPDRS-II slope (−0.27 points/year, 95% CI −0.52 to −0.01), indicating slower rather than faster worsening among exposed participants and not reproduced under inverse-probability weighting, complete-case analysis, or multiple imputation. These findings are interpreted cautiously in the context of multiple testing.

Supplementary Table 20. Drug-class and deep-brain-stimulation (DBS) sensitivity analyses.

Cohort	Scale	Effect	Primary	+ drug classes	+ DBS	Excl. DBS
PPMI	UPDRS-II	Severity	1.97 (1.33, 2.60)	2.00 (1.37, 2.63)	1.97 (1.33, 2.60)	1.91 (1.26, 2.57)
		Progression	0.03 (-0.18, 0.24)	0.04 (-0.16, 0.25)	0.03 (-0.18, 0.24)	0.00 (-0.21, 0.22)
PPMI	PDAQ-15	Severity	-2.40 (-3.23, -1.57)	-2.39 (-3.22, -1.55)	-2.42 (-3.25, -1.59)	-2.58 (-3.44, -1.71)
		Progression	-0.00 (-0.31, 0.31)	0.01 (-0.31, 0.32)	-0.00 (-0.31, 0.31)	-0.01 (-0.34, 0.32)
PPMI	GDS	Severity	0.33 (0.20, 0.47)	0.34 (0.21, 0.48)	0.33 (0.20, 0.47)	0.34 (0.20, 0.48)
		Progression	0.07 (0.01, 0.13)	0.07 (0.01, 0.13)	0.07 (0.01, 0.13)	0.08 (0.01, 0.14)
PPMI	PAS	Severity	2.00 (1.35, 2.64)	2.02 (1.38, 2.66)	2.00 (1.36, 2.65)	2.05 (1.39, 2.71)
		Progression	0.18 (-0.03, 0.40)	0.20 (-0.01, 0.42)	0.18 (-0.03, 0.40)	0.15 (-0.07, 0.37)
PPMI	ESS	Severity	0.63 (0.24, 1.03)	0.59 (0.20, 0.98)	0.64 (0.24, 1.03)	0.61 (0.20, 1.02)
		Progression	-0.01 (-0.20, 0.17)	-0.06 (-0.24, 0.12)	-0.01 (-0.20, 0.17)	0.00 (-0.19, 0.20)
PPMI	PDSS-2	Severity	2.24 (1.50, 2.97)	2.26 (1.53, 2.99)	2.24 (1.50, 2.97)	2.21 (1.45, 2.97)
		Progression	-0.17 (-0.51, 0.16)	-0.16 (-0.50, 0.18)	-0.17 (-0.51, 0.16)	-0.17 (-0.53, 0.18)
FoxInsight	UPDRS-II	Severity	1.02 (-0.20, 2.25)	1.02 (-0.20, 2.25)	1.01 (-0.22, 2.23)	0.91 (-0.50, 2.32)
		Progression	-0.27 (-0.52, -0.01)	-0.27 (-0.52, -0.01)	-0.27 (-0.52, -0.01)	-0.27 (-0.57, 0.03)
FoxInsight	PDAQ-15	Severity	-1.58 (-3.12, -0.05)	-1.57 (-3.11, -0.04)	-1.54 (-3.07, -0.03)	-1.99 (-3.73, -0.25)
		Progression	0.14 (-0.14, 0.41)	0.14 (-0.13, 0.42)	0.14 (-0.14, 0.41)	0.13 (-0.19, 0.44)
FoxInsight	GDS	Severity	-0.10 (-0.47, 0.28)	-0.09 (-0.46, 0.28)	-0.11 (-0.48, 0.27)	-0.14 (-0.58, 0.30)
		Progression	-0.06 (-0.18, 0.07)	-0.06 (-0.18, 0.07)	-0.06 (-0.18, 0.07)	-0.06 (-0.21, 0.09)

Fixed-effect estimates from linear mixed-effects models testing whether the exposure associations are robust to adjustment for the specific type of anti-parkinsonian treatment and for neurosurgical treatment. Because anti-parkinsonian drug class and DBS are downstream consequences of disease severity (and therefore mediators), these analyses are deliberately conservative over-adjustment checks rather than confounding adjustments. Models used the primary LMM specification with additional fixed effects, fitted in three ways: (i) adjustment for time-varying use of each anti-parkinsonian drug class (levodopa, dopamine agonist, MAO-B inhibitor, COMT inhibitor, amantadine, and anticholinergic; visit-level prevalence at medication visits: levodopa 84–86%, dopamine agonist 18–27%, MAO-B inhibitor 16–25%, amantadine 13–14%, COMT inhibitor 5–6%, anticholinergic 2%); (ii) adjustment for ever having undergone DBS; and (iii)

exclusion of participants with any DBS record (117 in PPMI Online and 114 in Fox Insight). DBS status was obtained from the PPMI Online treatment items and from the Fox Insight procedure questionnaire. For each cohort and scale the table reports the exposure main effect (“Severity”) and the time × exposure interaction (“Progression”) as β with 95% CI, alongside the primary estimate. The exposure associations were essentially unchanged throughout (for example, PPMI Online UPDRS-II 1.97 primary versus 2.00 with drug-class adjustment, 1.97 with DBS adjustment, and 1.91 excluding DBS participants), indicating that the findings are not explained by the type of anti-parkinsonian therapy or by surgical treatment.

Supplementary Table 21. Annual progression slope within each exposure group and the slope difference (exposure×time interaction).

Cohort	Scale	Unexposed slope	Exposed slope	Slope difference (exposed − unexposed)
PPMI	UPDRS-II	1.14 (0.99, 1.29)	1.17 (1.02, 1.32)	0.03 (-0.18, 0.24)
PPMI	GDS	0.01 (-0.04, 0.05)	0.07 (0.03, 0.12)	0.07 (0.01, 0.13)
PPMI	PDAQ-15	-0.97 (-1.19, -0.76)	-0.97 (-1.19, -0.75)	-0.00 (-0.31, 0.31)
PPMI	ESS	0.26 (0.13, 0.38)	0.24 (0.11, 0.38)	-0.02 (-0.20, 0.17)
PPMI	PDSS-2	0.32 (0.09, 0.55)	0.15 (-0.09, 0.39)	-0.17 (-0.51, 0.16)
PPMI	PAS	-0.03 (-0.18, 0.12)	0.16 (0.01, 0.31)	0.18 (-0.03, 0.40)
FoxInsight	UPDRS-II	1.16 (0.99, 1.34)	0.90 (0.72, 1.08)	-0.27 (-0.52, -0.01)
FoxInsight	GDS	0.26 (0.17, 0.35)	0.20 (0.11, 0.29)	-0.06 (-0.18, 0.07)
FoxInsight	PDAQ-15	-1.10 (-1.29, -0.91)	-0.97 (-1.16, -0.77)	0.14 (-0.14, 0.41)

The estimated annual rate of change in each scale score within the unexposed and exposed groups, together with their difference, derived from the primary linear mixed-effects models to display the progression rates underlying the exposure × time interaction reported in Supplementary Tables 3 and 19. In the primary LMM specification the unexposed slope equals the time coefficient, the exposed slope equals the time coefficient plus the time × exposure interaction, and the slope difference equals the time × exposure interaction; confidence intervals for the exposed slope were obtained from the model covariance matrix. The table reports, for each cohort and scale, the unexposed slope, the exposed slope, and their difference, each as β per year with 95% CI (for PDAQ-15, lower scores indicate worse function). The two group-specific slopes were nearly parallel for most outcomes (for example, PPMI Online UPDRS-II 1.14 in unexposed versus 1.17 in exposed participants, difference 0.03, 95% CI −0.18 to 0.24), and only two slope differences were nominally significant and inconsistent in direction and domain (a small positive PPMI Online GDS slope, 0.01 versus 0.07, difference 0.07; and a negative Fox Insight UPDRS-II slope, 1.16 versus 0.90, difference −0.27). This presentation directly addresses the request to report the slope difference between exposure groups and supports the absence of a consistent pattern of accelerated progression.

Supplementary Table 22. Milestone (Cox) hazard ratios for pesticide exposure across all sensitivity analyses.

Cohort	Endpoint	Primary	+ baseline med	IPTW	Complete-case	MICE	+ drug class	+ DBS	Excl. DBS	Duration <5y
PPMI	UPDRS2 > 12	1.22 (1.00, 1.48)	1.20 (0.99, 1.46)	1.22 (1.03, 1.44)	1.19 (0.84, 1.67)	1.20 (0.97, 1.49)	1.21 (1.00, 1.47)	1.20 (0.99, 1.46)	1.21 (0.98, 1.48)	1.23 (0.99, 1.52)
PPMI	PDAQ-15 < 37	1.28 (0.87, 1.87)	1.27 (0.86, 1.87)	1.37 (1.00, 1.89)	1.14 (0.57, 2.28)	1.36 (0.87, 2.13)	1.28 (0.87, 1.88)	1.28 (0.87, 1.88)	1.33 (0.89, 1.99)	1.30 (0.84, 2.00)
PPMI	GDS > 5	1.02 (0.85, 1.22)	1.01 (0.85, 1.21)	1.07 (0.92, 1.24)	1.36 (1.00, 1.86)	1.06 (0.86, 1.29)	1.02 (0.85, 1.22)	1.02 (0.85, 1.21)	1.06 (0.88, 1.27)	1.04 (0.85, 1.27)
PPMI	ESS > 10	1.59 (1.19, 2.13)	1.62 (1.21, 2.16)	1.53 (1.22, 1.93)	1.06 (0.65, 1.73)	1.53 (1.13, 2.09)	1.63 (1.22, 2.19)	1.61 (1.21, 2.15)	1.63 (1.21, 2.19)	1.56 (1.12, 2.15)
PPMI	PAS total > 13	1.28 (1.05, 1.55)	1.27 (1.05, 1.55)	1.34 (1.14, 1.57)	1.40 (1.00, 1.95)	1.26 (1.00, 1.58)	1.31 (1.07, 1.59)	1.28 (1.05, 1.55)	1.28 (1.05, 1.57)	1.24 (0.99, 1.54)
FoxInsight	UPDRS2 > 12	1.33 (1.01, 1.75)	1.33 (1.01, 1.75)	1.26 (1.01, 1.56)	1.29 (0.96, 1.74)	1.26 (0.95, 1.65)	1.33 (1.01, 1.75)	1.33 (1.01, 1.75)	1.32 (0.98, 1.79)	1.32 (0.97, 1.80)
FoxInsight	PDAQ-15 < 37	1.26 (0.83, 1.91)	1.27 (0.84, 1.92)	1.28 (0.94, 1.72)	1.45 (0.91, 2.31)	1.28 (0.84, 1.95)	1.23 (0.81, 1.87)	1.25 (0.83, 1.90)	1.45 (0.92, 2.30)	1.57 (0.96, 2.57)
FoxInsight	GDS > 5	0.87 (0.64, 1.17)	0.87 (0.64, 1.17)	0.87 (0.69, 1.10)	0.93 (0.66, 1.30)	0.85 (0.63, 1.14)	0.91 (0.67, 1.23)	0.87 (0.65, 1.18)	0.85 (0.61, 1.18)	0.95 (0.68, 1.32)

Exposure hazard ratios from Cox proportional hazards models for each clinically meaningful milestone event, repeated under the same sensitivity analyses applied to the continuous outcomes, so that the milestone (time-to-event) results have a robustness battery parallel to that for symptom severity. As in the primary milestone analysis (Supplementary Table 4), models were fitted within the propensity score–matched cohorts (except the IPTW model, fitted in the full eligible cohort), restricted to participants who had not reached the corresponding threshold at baseline, with pesticide exposure as the primary predictor and baseline age, sex, and disease duration as covariates; medication and drug class enter as baseline status because the outcome is time-to-threshold measured from baseline. For each cohort and endpoint the table reports the exposure hazard ratio (95% CI) under the primary model and under: adjustment for baseline anti-parkinsonian medication; full-cohort inverse-probability weighting (weighted Cox with robust standard errors); complete-case and multiple-imputation (20 imputed datasets, log-hazard ratios pooled by Rubin's rules) re-matched analyses; adjustment for baseline drug classes (levodopa, dopamine agonist, MAO-B inhibitor); exclusion of participants with any DBS record; and restriction to less than five years of baseline disease duration. The hazard ratios were stable across analyses (for example, the PPMI Online motor milestone 1.22 in the primary model versus 1.19–1.23 across analyses, and the daytime-sleepiness milestone 1.59 versus 1.53–1.63; the wider complete-case ESS interval reflects the smaller complete-case sample), indicating that the milestone associations are not explained by differential treatment, the matched-cohort design, the handling of missing data, or prevalent-cohort entry. These analyses complement the baseline-severity-adjusted and delayed-entry models in Supplementary Table 17.

Supplementary Note 1. Additional Results for Other Exposure Characteristics

In addition to the number of pesticide classes involved in co-exposure reported in the main text, we conducted exploratory analyses within the exposed group to examine the associations of cumulative exposure duration, personal protective equipment (PPE) use, and specific pesticide types with the severity and progression rate of each symptom scale. All models were adjusted for baseline age, sex, and baseline disease duration, and included time, the exposure characteristic of interest, and their interaction term. The results are presented below by exposure variable, and the full data are provided in Supplementary Tables 6 and 7.

Cumulative Exposure Duration

For most outcomes, cumulative exposure duration showed no significant main effect and no significant interaction with time. The only exception was the PDAQ-15. In the PPMI cohort, each additional year of cumulative exposure was associated with a 0.08-point lower baseline PDAQ-15 score ($\beta = -0.078$, 95% CI: −0.139 to −0.017, $p = 0.012$), suggesting poorer cognition-related daily functioning with longer exposure duration. A similar directional trend was observed in the Fox Insight cohort ($\beta = -0.070$, 95% CI: −0.181 to 0.040), although the association was not statistically significant ($p = 0.211$). In both cohorts, the interaction between cumulative exposure duration and time was not significant (PPMI: $\beta = 0.013$, $p = 0.245$; Fox Insight: $\beta = 0.017$, $p = 0.098$), indicating that cumulative exposure duration was not associated with the rate of progression in cognition-related daily functioning. For the other scales, including UPDRS-II, GDS, ESS, PAS, and PDSS-2, neither the main effect nor the interaction with time reached statistical significance in either cohort.

Personal Protective Equipment (PPE) Use

The effect of PPE use reached statistical significance only for isolated outcomes in a single cohort. In the PPMI cohort, participants who reported using PPE had a 1.72-point higher baseline PDSS-2 score than those who did not (95% CI: 0.46 to 2.97, $p = 0.007$), indicating poorer sleep quality among PPE users. This apparently paradoxical finding may reflect confounding by exposure intensity, as individuals who use PPE are often those with more intensive exposure, such as workers with long-term occupational exposure. Consequently, despite using protective equipment, their actual exposure burden may still have been greater than that of individuals with only occasional exposure who did not use PPE. The interaction between PPE use and time was not significant ($\beta = -0.207$, $p = 0.475$), indicating that PPE use was not associated with the rate of progression of sleep-related symptoms.

For anxiety (PAS), the PPE use × time interaction showed a borderline trend in the PPMI cohort (β = −0.358, 95% CI: −0.731 to 0.015, p = 0.060), suggesting that anxiety symptoms may have progressed slightly more slowly among PPE users, although the result did not reach conventional statistical significance. For the remaining scales, including UPDRS-II, PDAQ-15, ESS, and GDS, neither the main effect nor the interaction with time for PPE use was significant in either cohort.

Specific Pesticide Types

We further examined the independent effects of fungicides, insecticides, herbicides, fumigants (PPMI only), and rodenticides (Fox Insight only) on symptom outcomes. Because some pesticide types were available in only one cohort, cross-cohort comparison was limited.

3.1 Fungicides

In the PPMI cohort, fungicide exposure showed both a significant main effect and a significant interaction with time for sleep quality, as measured by the PDSS-2. Compared with participants without fungicide exposure, those with fungicide exposure had a 1.70-point higher baseline PDSS-2 score (95% CI: 0.27 to 3.13, p = 0.020), and their score increased by an additional 0.71 points per year (interaction β = 0.714, 95% CI: 0.08 to 1.35, p = 0.026). These findings suggest that fungicide exposure was associated not only with poorer sleep quality at baseline but also with a faster rate of deterioration over time. However, because PDSS-2 was available only in the PPMI cohort, this result could not be validated across cohorts. Fungicide exposure was not significantly associated with the other scales in either cohort.

3.2 Insecticides

In the PPMI cohort, insecticide exposure showed a significant negative interaction with time for PDSS-2 scores (β = −0.833, 95% CI: −1.41 to −0.25, p = 0.005), suggesting a slower rate of worsening in sleep quality among participants with insecticide exposure than among those without such exposure. This pattern was opposite to that observed for fungicides. The main effect of insecticide exposure on PDSS-2 was not statistically significant (β = 1.137, 95% CI: −0.15 to 2.42, p = 0.083). In the Fox Insight cohort, the interaction between insecticide exposure and time for UPDRS-II progression showed a borderline negative trend (β = −0.414, 95% CI: −0.87 to 0.04, p = 0.071), in the same direction. A borderline negative interaction with time was also observed for GDS progression in the PPMI cohort (β = −0.088, 95% CI: −0.19 to 0.01, p = 0.089), although no similar effect was observed in the Fox Insight cohort. No significant associations were found for the remaining scales.

3.3 Herbicides

In the PPMI cohort, herbicide exposure showed a significant negative interaction with time for anxiety symptoms, as measured by the PAS ($\beta = -0.481$, 95% CI: −0.96 to 0.00, $p = 0.049$), suggesting a slower rate of anxiety worsening among herbicide-exposed participants. However, because PAS was assessed only in the PPMI cohort, this finding could not be validated across cohorts. In the Fox Insight cohort, herbicide exposure showed a borderline negative association with baseline PDAQ-15 score ($\beta = -2.507$, 95% CI: −5.31 to 0.30, $p = 0.080$), although this association did not reach statistical significance. No significant effects of herbicide exposure were observed for the remaining scales in either cohort.

3.4 Fumigants and Rodenticides

Fumigant exposure, which was assessable only in the PPMI cohort, showed no significant main effects or interactions with time for any scale. The only suggestive finding was a borderline positive main effect for UPDRS-II ($\beta = 1.555$, 95% CI: −0.09 to 3.20, $p = 0.064$). Rodenticide exposure, which was assessable only in the Fox Insight cohort, likewise showed no significant associations with any scale. However, a borderline positive main effect was observed for UPDRS-II ($\beta = 2.249$, 95% CI: −0.02 to 4.52, $p = 0.052$), and a borderline negative interaction with time was observed for GDS progression ($\beta = -0.131$, 95% CI: −0.28 to 0.02, $p = 0.084$). Because these findings were derived from a single cohort and did not reach conventional statistical significance, they should be interpreted with caution.

Conclusion

Overall, aside from the dose–response relationship for the number of pesticide classes involved in co-exposure reported in the main text, the other exposure characteristics—including cumulative exposure duration, early-life exposure, PPE use, and specific pesticide types—showed limited associations with PD symptom severity or progression rate. The few significant or borderline significant findings were scattered across individual scales or single cohorts and were not consistently replicated across cohorts, suggesting that they may represent chance findings or reflect limited statistical power. Among these exploratory results, the association between fungicide exposure and both baseline PDSS-2 score and PDSS-2 progression in the PPMI cohort deserves further investigation. However, this finding should be validated in independent cohorts with comparable sleep-related measures.
